# Volumetric trajectories of hippocampal subfields and amygdala nuclei influenced by adolescent alcohol use and lifetime trauma

**DOI:** 10.1038/s41398-021-01275-0

**Published:** 2021-03-02

**Authors:** Rachel D. Phillips, Michael D. De Bellis, Ty Brumback, Ashley N. Clausen, Emily K. Clarke-Rubright, Courtney C. Haswell, Rajendra A. Morey

**Affiliations:** 1 Duke-UNC Brain Imaging and Analysis Center, Duke University, Durham, NC USA; 2 Department of Psychiatry and Behavioral Sciences, Duke University, Durham, NC USA; 3 Healthy Childhood Brain Development/Developmental Traumatology Research Program, Department of Psychiatry and Behavioral Sciences, Duke University Medical Center, NC Duke University, Durham, NC USA; 4 Northern Kentucky University, Highland Heights, KY USA; 5 Durham VA Medical Center, Durham, NC USA

**Keywords:** Predictive markers, Psychiatric disorders

## Abstract

Alcohol use and exposure to psychological trauma frequently co-occur in adolescence and share many risk factors. Both exposures have deleterious effects on the brain during this sensitive developmental period, particularly on the hippocampus and amygdala. However, very little is known about the individual and interactive effects of trauma and alcohol exposure and their specific effects on functionally distinct substructures within the adolescent hippocampus and amygdala. Adolescents from a large longitudinal sample (*N* = 803, 2684 scans, 51% female, and 75% White/Caucasian) ranging in age from 12 to 21 years were interviewed about exposure to traumatic events at their baseline evaluation. Assessments for alcohol use and structural magnetic resonance imaging scans were completed at baseline and repeated annually to examine neurodevelopmental trajectories. Hippocampal and amygdala subregions were segmented using Freesurfer v6.0 tools, followed by volumetric analysis with generalized additive mixed models. Longitudinal statistical models examined the effects of cumulative lifetime trauma measured at baseline and alcohol use measured annually on trajectories of hippocampal and amygdala subregions, while controlling for covariates known to impact brain development. Greater alcohol use, quantified using the Cahalan scale and measured annually, was associated with smaller whole hippocampus (*β* = −12.0, *p*_FDR_ = 0.009) and left hippocampus tail volumes (*β* = −1.2, *p*_FDR_ = 0.048), and larger right CA3 head (*β* = 0.4, *p*_FDR_ = 0.027) and left subiculum (*β* = 0.7, *p*_FDR_ = 0.046) volumes of the hippocampus. In the amygdala, greater alcohol use was associated with larger right basal nucleus volume (*β* = 1.3, *p*_FDR_ = 0.040). The effect of traumatic life events measured at baseline was associated with larger right CA3 head volume (*β* = 1.3, *p*_FDR_ = 0.041) in the hippocampus. We observed an interaction between baseline trauma and within-person age change where younger adolescents with greater trauma exposure at baseline had smaller left hippocampal subfield volumes in the subiculum (*β* = 0.3, *p*_FDR_ = 0.029) and molecular layer HP head (*β* = 0.3, *p*_FDR_ = 0.041). The interaction also revealed that older adolescents with greater trauma exposure at baseline had larger right amygdala nucleus volume in the paralaminar nucleus (*β* = 0.1, *p*_FDR_ = 0.045), yet smaller whole amygdala volume overall (*β* = −3.7, *p*_FDR_ = 0.003). Lastly, we observed an interaction between alcohol use and baseline trauma such that adolescents who reported greater alcohol use with greater baseline trauma showed smaller right hippocampal subfield volumes in the CA1 head (*β* = −1.1, *p*_FDR_ = 0.011) and hippocampal head (*β* = −2.6, *p*_FDR_ = 0.025), yet larger whole hippocampus volume overall (*β* = 10.0, *p*_FDR_ = 0.032). Cumulative lifetime trauma measured at baseline and alcohol use measured annually interact to affect the volume and trajectory of hippocampal and amygdala substructures (measured via structural MRI annually), regions that are essential for emotion regulation and memory. Our findings demonstrate the value of examining these substructures and support the hypothesis that the amygdala and hippocampus are not homogeneous brain regions.

## Introduction

Early-life trauma and alcohol use disorders in the adolescent period are often co-morbid^1^. A robust link has been established between exposure to childhood trauma and adolescent binge drinking and alcohol misuse^2^. Thus, experiencing early-life physical trauma is six times more likely, and experiencing sexual trauma is 18 times more likely among adolescents with alcohol use disorder (AUD), constituting a major risk factor^1,3^. Harmful patterns of alcohol use often present in adolescence among at-risk individuals, which offers a valuable window for exploring pathways between childhood trauma and the onset of AUD. Emotional dysregulation such as impulsivity and mood lability that are associated with childhood trauma, are potent risk factors for risky behaviors including alcohol and illicit substance use^4–6^. Consequently, the impact of heavy alcohol use during adolescence on individuals with early-life trauma may lead to a life-course with persistent AUD by impairing neural systems that regulate goal-directed behaviors, inhibition, memory, anxiety, and fear responses^7^. Conversely, alcohol intake impairs executive control and undermines the function of the reward system^8^.

Several cross-sectional studies have shown that experiencing DSM criteria-A trauma in childhood^9–13^ and exposure to alcohol during adolescence both independently influence amygdala and hippocampal structure and function negatively. Evidence from functional neuroimaging shows that early-life trauma exposure impairs top–down prefrontal control of the limbic system^1,14^. The resulting disinhibition of limbic processes poses a risk factor for adolescent alcohol use which itself promotes further behavioral disinhibition and impulsivity^15^ that may manifest as binge drinking. Preclinical studies demonstrate that the hippocampus and amygdala are altered by both early-life stress^16^ and adolescent alcohol use^17,18^, which is associated with disruptions of hippocampal neurogenesis. However, most human studies are focused on adults rather than adolescents and on total hippocampal and amygdala volumes^19,20^. Investigations of subregions within the adolescent hippocampus and amygdala are vastly underperformed^21^. The effects of heavy alcohol use in adolescence and its interaction with early-life trauma on the developmental trajectories of amygdala and hippocampal subregions volume are not known. Furthermore, the amygdala and hippocampus are not unitary structures. Each structure is composed of several subregions representing manifold functions that mediate distinct emotional and behavioral responses.

Each amygdala subregion communicates with other amygdala subregions, subcortical regions, and cortical regions in the setting and aftermath of trauma to elicit distinct behavioral responses, akin to fight or flight, and cognitive responses such as associative fear learning. Each subcortical structure is composed of several subregions representing multifarious functions that mediate responses to trauma exposure. Likewise, these subcortical structures play equally important, but functionally different roles in adolescents with AUD. For instance, the critical amygdala function of fear perception and responding to threatening stimuli is significantly reduced by alcohol intake^22^. Evidence in humans shows that the anxiolytic effects of alcohol are mediated by the amygdala. For instance, nuclei-specific hypertrophic changes in the basolateral complex of the amygdala (BLA) accompany anxiety-like behavior after exposure to chronic, but not acute restraint stress in rodents^23^. Perturbations in the connection between the BLA and nucleus accumbens produce suboptimal decision-making by diverting choices to more risky options^24^. A substantial decrease in the inhibitory synaptic activity of BLA neurons follows long-term alcohol consumption by way of cellular and molecular circuit-level adaptations. This undermining of inhibitory control in the BLA is thought to explain the high incidence of compulsive drinking and anxiety-induced relapse in patients with alcohol use disorders^25^. Similarly, the central and medial nuclei of the amygdala are important for mediating responses to fear^26^. Specifically, the central nucleus of the amygdala is essential to limbic activity required in freezing and flight behaviors, and also a critical component in the mechanism of alcohol self-administration^27,28^. In fact, the ventral tegmental area dopamine system has major reciprocal connections with the central nucleus of the amygdala^29^.

Similarly, hippocampal subfield-specific functions play a critical role following trauma exposure. The dentate gyrus (DG) of the hippocampus is important in distinguishing features that are different from other memories in order to store similar memories as discrete events—a phenomenon called pattern separation^30,31^. Pattern separation deficits may underlie fear generalization^32^, a process that occurs in anxiety and stress-based disorders including posttraumatic stress disorder (PTSD)^33^. By contrast, the entorhinal cortex (EC) and cornu ammonis subfield‐3 (CA3) of the hippocampus are crucial in distinguishing events with overlapping features—a phenomenon called *pattern completion* that has important implications in contextual fear conditioning^34^. Longitudinal studies showed accelerated gray matter decline in the hippocampus and parahippocampus of college students^21^ and a decrease in hippocampal volume more generally among adolescents^35^. Subregion-specific effects of alcohol were found in the dentate gyrus of the hippocampus. Alcohol modulated molecular mediators are thought to be involved in experiencing interoceptive cues that regulate reward-seeking behavior. Moreover, adults with AUD have been shown to have age-dependent atrophy of CA-2 and CA3 hippocampal subfields^36^. However, the investigations of alcohol use on specific subregions have received minimal attention particularly in adolescents^37–40^.

In this longitudinal study, we measured the effects of early-life trauma and alcohol use on the developmental trajectories of the amygdala and hippocampal volume subregions in adolescents age 12–21 years in the National Consortium on Alcohol and Neurodevelopment in Adolescence (NCANDA) study^38^. NCANDA has an accelerated longitudinal design, which enrolls multiple single cohorts, each one starting at a different age. Its main advantage is in its ability to span the age range of interest in a shorter period of time than would be possible with a single cohort longitudinal design. The NCANDA study examined the psychological, environmental, neuro-predictors, and neuro-consequences of adolescent alcohol use in a large diverse community sample. Here, we examined the conditioned main and interactive effects of trauma and alcohol use on specific subregions of the amygdala^41,42^ and hippocampus^43^ as these subregions have different functions and their developmental trajectories may be differentially affected by heavy alcohol use. Although previous studies have examined the association between alcohol use and trauma on total amygdala and hippocampal volumes, to our knowledge, this is the first study to examine the effects of alcohol use and early-life trauma on the developmental trajectories of these subregions. We hypothesize that heavy alcohol use, early trauma at baseline, and their interactions throughout the longitudinal observation period will be associated with an increase or decrease in the rate of subregional volume change during the adolescent period. While the study of subregion-specific impacts is largely exploratory, we expect that the functional specialization of subregions may influence subregion-specific impacts from trauma and alcohol use. Given the lack of literature, it is not possible for us to generate informed subregion-specific hypotheses as each subregion may undergo distinct hypertrophic or atrophic effects under the independent or combined environmental insults of alcohol use and trauma.

## Methods and materials

### Participants

Adolescents (*n* = 803) ages 12–21 at baseline, were recruited across five NCANDA sites: University of California at San Diego (*n* = 210); Duke University Medical Center (*n* = 169); SRI International (*n* = 160); Oregon Health and Science University (*n* = 149); and University of Pittsburgh Medical Center (*n* = 121). The administrative component (UCSD) and the data analysis and informatics component (SRI) facilitated the training, quality assurance, and data integration across sites. The institutional review board at each site approved the study. Adult participants consented to participate, and minors provided written assent along with consent from a parent/legal guardian. Baseline exclusionary criteria included serious medical, mental health, or learning disorders. NCANDA’s primary aim is to determine the neurobiological effects of alcohol use, and exclusion criteria required the majority of participants to meet CDC guidelines for normalized adolescent experimentation with alcohol, meaning limited exposure to alcohol and other drugs such as marijuana or nicotine. The entire NCANDA sample across sites was limited such that only a subset (17%) of enrolled youth could exceed alcohol use thresholds for alcohol only at baseline. Alcohol use thresholds varied by age and sex, and the maximum allowable drinks on any one occasion are detailed in previous NCANDA publications.

Adolescents were also screened for risk status, to sufficiently include at-risk youth who were more likely to initiate heavy alcohol use during the follow-up assessments. Criteria for heavy alcohol use risk were: (1) Initiation of alcohol use before age 15; (2) Positive family history of AUD; (3) One or more externalizing symptoms (e.g., conduct disorder); or (4) Two or more internalizing symptoms (e.g., depression or anxiety). Roughly 50% of the sample met high-risk criteria^38^. The NCANDA sample available through the follow-up-3 data release, which is presented here, includes 831 adolescents at baseline. Twenty-eight participants were removed from the dataset following failure of one or more steps in the FreeSurfer longitudinal stream (*n* = 2), hippocampus/amygdala segmentation (*n* = 17), or missing alcohol or drug use data (*n* = 9). Of the 803 subjects included in the present analyses, 739 returned in 1 year for an annual follow-up, 651 for follow-up 2, and 491 for follow-up 3. A total of 2,684 individual study visits are included in the present study. See Table 1 for sample demographic characteristics stratified by study visit. See Supplementary Table S1 for demographic characteristics of the sample at baseline by the site.

**Table 1:** Demographic characteristics at baseline and follow-ups (FU) 1, 2, and 3.

	Overall^a^ *n* = 2684	Baseline^a^ *n* = 803	FU1^a^ *n* = 739	FU2^a^ *n* = 651	FU3^a^ *n* = 491	*p*-value^b^
Age at scan (years)	17.58 (2.73) [12.02–25.07]	16.21 (2.52) [12.02–21.96]	17.30 (2.49) [12.99–22.93]	18.29 (2.46) [13.98–23.98]	19.32 (2.49) [14.92–25.07]	**<0.001**
Sex						>0.9
F	1380 (51%)	412 (51%)	381 (52%)	332 (51%)	255 (52%)	
M	1304 (49%)	391 (49%)	358 (48%)	319 (49%)	236 (48%)	
Race						
African-American/Black	310 (12%)	96 (12%)	87 (12%)	73 (11%)	54 (11%)	
African-American and Caucasian	29 (1.1%)	9 (1.1%)	8 (1.1%)	8 (1.2%)	4 (0.8%)	
Asian	210 (7.8%)	64 (8.0%)	59 (8.0%)	51 (7.8%)	36 (7.3%)	
Asian Pacific Islander	4 (0.1%)	1 (0.1%)	1 (0.1%)	1 (0.2%)	1 (0.2%)	
Asian and White	74 (2.8%)	21 (2.6%)	20 (2.7%)	19 (2.9%)	14 (2.9%)	
Caucasian/White	2024 (75%)	600 (75%)	555 (75%)	491 (75%)	378 (77%)	
Native American/American Indian	7 (0.3%)	3 (0.4%)	2 (0.3%)	2 (0.3%)	0 (0%)	
Native American and Caucasian	5 (0.2%)	2 (0.2%)	1 (0.1%)	1 (0.2%)	1 (0.2%)	
None	6 (0.2%)	2 (0.2%)	2 (0.3%)	1 (0.2%)	1 (0.2%)	
Pacific Islander	12 (0.4%)	4 (0.5%)	3 (0.4%)	3 (0.5%)	2 (0.4%)	
Pacific Islander and Caucasian	3 (0.1%)	1 (0.1%)	1 (0.1%)	1 (0.2%)	0 (0%)	
SES	16.79 (2.47) [6.00–20.00]	16.79 (2.49) [6.00–20.00]	16.83 (2.45) [6.00–20.00]	16.81 (2.48) [6.00–20.00]	16.71 (2.45) [6.00–20.00]	0.8
Family AUD density						
0	2008 (75%)	601 (75%)	554 (75%)	487 (75%)	366 (75%)	
0.5	392 (15%)	117 (15%)	108 (15%)	96 (15%)	71 (14%)	
1	158 (5.9%)	46 (5.7%)	42 (5.7%)	39 (6.0%)	31 (6.3%)	
1.5	72 (2.7%)	22 (2.7%)	19 (2.6%)	17 (2.6%)	14 (2.9%)	
2	45 (1.7%)	14 (1.7%)	13 (1.8%)	10 (1.5%)	8 (1.6%)	
2.5	4 (0.1%)	1 (0.1%)	1 (0.1%)	1 (0.2%)	1 (0.2%)	
3	3 (0.1%)	1 (0.1%)	1 (0.1%)	1 (0.2%)	0 (0%)	
4	2 (<0.1%)	1 (0.1%)	1 (0.1%)	0 (0%)	0 (0%)	
Drinking class (Cahalan scale)						**<0.001**
0	1800 (67%)	642 (80%)	512 (69%)	385 (59%)	261 (53%)	
1	471 (18%)	110 (14%)	122 (17%)	131 (20%)	108 (22%)	
2	106 (3.9%)	12 (1.5%)	19 (2.6%)	32 (4.9%)	43 (8.8%)	
	307 (11%)	39 (4.9%)	86 (12%)	103 (16%)	79 (16%)	
Baseline trauma (# of events)						>0.9
0	1029 (38%)	305 (38%)	286 (39%)	251 (39%)	187 (38%)	
1	887 (33%)	265 (33%)	239 (32%)	218 (33%)	165 (34%)	
2	508 (19%)	154 (19%)	144 (19%)	121 (19%)	89 (18%)	
3	181 (6.7%)	56 (7.0%)	49 (6.6%)	42 (6.5%)	34 (6.9%)	
4	79 (2.9%)	23 (2.9%)	21 (2.8%)	19 (2.9%)	16 (3.3%)	
Lifetime marijuana use (days)	26.15 (137.2) [0–2309]	8.80 (86.3) [0–1712]	16.78 (103.9) [0–1738]	32.80 (146.5) [0–2119]	59.82 (210.7) [0–2309]	**<0.001**
Lifetime tobacco use (days)	19.37 (247.6) [0–10796]	3.11 (36.4) [0–900]	12.39 (106.4) [0–1827]	19.80 (169.9) [0–3656]	55.92 (525.6) [0–10,796]	**<0.001**

*n* (%).

^a^Statistics presented: mean (SD) [minimum-maximum].

^b^Statistical tests performed: Kruskal–Wallis test; chi-square test of independence.

Bold values indicates statistical significant *p*-values (*p* < 0.05).

### Clinical measures

Drinking class was measured at baseline and subsequent follow-ups to capture alcohol use over time. Categorized by a modified Cahalan inventory^44^ drinking class was used to quantify both the quantity (average and maximum consumption) and frequency of alcohol use within the past year (Supplementary Fig. S1). “No to low” drinkers (e.g., drinking class value of 0) reported no or low quantity and frequency consumption (e.g., <1×/month, <2 drinks on average, and <4 drinks maximum). “Moderate” drinkers (i.e., drinking class value of 1) ranged from low alcohol use frequency (e.g., <1×/month) with moderate quantity consumption (e.g., with 2–3 drinks on average and 4–5 drinks maximum) to moderate frequency (e.g., 1×/week) and low quantity consumption (e.g., with 2 drinks on average and <4 drinks maximum). “Heavy” drinkers (i.e., drinking class value of 2) ranged from moderate frequency (e.g., 2×/month) with high quantity consumption (e.g., with 3–4 drinks on average) to a higher frequency (e.g., 1×/week or more) with moderate quantity consumption (e.g., with 2–3 drinks on average). Lastly, “Heavy binge” drinkers (i.e., drinking class value of 3) reported heavy use, with higher quantity consumption (>4 drinks).

Age at initiation of regular alcohol use was assessed using the Customary Drinking and Drug Use Record (CDDR)^45^. On the CDDR, regular use was defined as consuming alcohol (i.e., beer, wine, or liquor) at least once a week. Cumulative trauma at baseline was quantified as the sum of reported DSM-IV or 5 Criterion A traumatic events on the PTSD section of the Semi-Structured Assessment for the Genetics of Alcoholism^46^. Cumulative (i.e., lifetime) trauma at baseline for an individual was labeled as 0, for no reported traumatic events, 1, for a single reported traumatic event, 2, for two traumatic events, 3, for three traumatic events, or 4, for four or more reported traumatic events. A traumatic event was counted once if either the parent and/or youth reported an event. Further information about the baseline traumas experienced in NCANDA was previously published^39^. See Supplementary Materials for more information on baseline trauma variable collection and Supplementary Table S2 for trauma types reported in the NCANDA sample. SES was quantified using the highest parental years of education of either parent^38^. Family history of Alcohol Use Disorder (AUD) density was calculated based on the presence of AUD in first and second-degree relatives (positive parents + positive grandparents * 0.5; yielding a range of 0–4)^47^.

### MRI acquisition

All five NCANDA sites used comparable anatomical MRI data collection protocol and 3T systems: 3T General Electric (GE) Discovery MR750 and 3T Siemens TIM TRIO. The GE sites (SRI, Duke, and UCSD) used an Array Spatial Sensitivity Encoding Technique (ASSET) for parallel and accelerated imaging with an eight-channel head coil and acquired an Inversion Recovery-SPoiled Gradient Recalled (IR-SPGR) echo sequence (TR = 5.912 ms, TI = 400 ms, TE = 1.932 ms, flip angle= 11°, NEX = 1, matrix= 256 × 256, FOV = 24 cm, voxel dimensions= 1.2 × 0.9375 × 0.9375 mm, 146 slices). The Siemens sites (Pittsburgh and OHSU) used a 12-channel head coil and parallel imaging and temporal acceleration with iPAT and acquired an MPRAGE sequence (TR = 1900 ms, TI = 900 ms, TE = 2.92 ms, flip angle= 9°, NEX = 1, matrix= 256 × 256, FOV = 24 cm, voxel dimensions= 1.2 × 0.9375 × 0.9375 mm, 160 slices).

### Longitudinal segmentation pipeline

To extract reliable volume estimates, all T1-weighted structural scans were processed longitudinally using FreeSurfer v6.0, which includes cross-sectional segmentation with longitudinal initialization. The hippocampus and amygdala for each structural scan with a resolution of 1.2 × 0.9375 × 0.9375 mm were simultaneously auto-segmented (Supplementary Fig. S2) using the FreeSurfer longitudinal segmentation pipeline by Iglesias and colleagues^48^. The longitudinal segmentation pipeline uses a probabilistic atlas built with ultra-high resolution ex vivo MRI data (~0.1 mm isotropic) to segment 19 hippocampal subfields and 9 amygdala nuclei^48,49^. For a list of all hippocampal subfields and amygdala nuclei (Supplementary Table S3). Neither the main longitudinal pipeline nor the longitudinal segmentation pipeline assumes any specific trajectory (i.e., volume increase or decrease over time) for the segmentation or corresponding volumes^48^. The hippocampus and amygdala are jointly segmented to avoid overlap or gaps between structures^49^. See Supplementary Materials for information on the test–retest reliability of the Freesurfer v6.0 longitudinal segmentation approach, and Supplementary Fig. S3 for intra-class correlations (ICC) of subregion volume estimation across NCANDA sites.

### Outlier detection and removal

Quality assurance was achieved through a two-step approach (1) statistically-based outlier detection, followed by (2) visual inspection. Outlier detection removed subregions whose volume was more than 2.69 standard deviations from the mean. All scans were included in calculating the standard deviation of substructure volume regardless of timepoint. Therefore, a specific structure could be excluded but the remaining structures for the same subject’s associated timepoints were retained in our analysis. Automated outlier detection was followed by visual inspection by three trained raters (RP, NB, and MM) to rule out mis-segmentation due to image artifacts. The number of outliers by subfield is included in Supplementary Table S3.

### Statistical modeling

NCANDA’s accelerated longitudinal design (e.g., cohort-sequential design), allows us to consider both the within-subject and within-cohort structural brain changes over the course of the study. Within-person age change represented the difference between a subject’s age at each scan and their mean age across individual timepoints. Cohort age represented the difference between a subject’s mean age across visits and the mean age of the entire sample across timepoints, thus centering cohort age at the sample mean. Each participant’s cohort age remained constant across timepoints.

Following previously implemented approaches for structured multi-cohort longitudinal designs, we modeled the developmental trajectories of hippocampal subfields and amygdala nuclei using a mixed-effects approach^50^. In this design, random effects accounted for the within-subject covariance across time. In all models, NCANDA site and participant identity were included as random intercepts. Within-person age change, cohort age, whole hippocampal or amygdala volume, sex, race, socioeconomic status (SES), drinking class, family history of AUD density^47^, and cumulative lifetime trauma at baseline were included as covariates for conditional likelihood. Covariates of race, sex, SES, trauma, and family history that were assessed at baseline were modeled as stable variables, which did not vary across time. This was achieved by repeating baseline values across time points per subject.

Two hierarchical models were tested across hippocampal subfields and amygdala nuclei for statistical significance. Model 1 was designed to evaluate the effects of traumatic life events as participants mature through an interaction term with age cohort, age change, and trauma, while also considering alcohol consumption (drinking class). Model 2 added an interaction term to evaluate whether alcohol consumption (drinking class) increased or decreased the effects of trauma from model 1.

*Model 1*: Hippo/Amyg Subfield Volume ~ Within-person Age Change*Cohort Age*Number of Traumatic Life Events + Drinking Class + Hippo/Amyg Whole Volume + Sex + Socioeconomic Status + Family History of AUD Density + Race + (1|Site) + (1|Subject)

*Model 2*: Hippo/Amyg Subfield Volume ~ Within-person Age Change*Cohort Age*Number of Traumatic Life Events + Drinking Class*Number of Traumatic Life Events + Hippo/Amyg Whole Volume + Sex + Socioeconomic Status + Family History of AUD Density + Race + (1|Site) + (1|Subject)

A secondary analysis of these models was performed while controlling for lifetime marijuana and tobacco use.

All analyses were conducted in the statistical program, R version 4.0.0 (www.R-project.org), using the gamm4 package for generalized additive mixed models (GAMM). All statistics reported were controlled for multiple comparisons per model and have survived false discovery rate (FDR) correction at *p* < 0.05^37^. Thus, the correction was based on 56 tests (28 subregions × 2 hemispheres) for model 1 and on 56 tests (28 subregions × 2 hemispheres) for model 2.

## Results

### Age at alcohol use initiation

A portion of the sample (*n* = 181) initiated regular alcohol use, characterized by consuming alcohol one or more times a week, during the course of the study. The average age at alcohol use initiation was 18.8 years, with a standard deviation of 1.84 years. Age at initiation ranged from 13.5 to 24.3 years old. Male (*n* = 93) and female (*n* = 88) participants did not differ in average age at initiation (*p* > 0.05). Fifty-two adolescents reported regular alcohol use initiation at baseline, thirty-nine at follow-up 1, fifty-four at follow-up 2, and thirty-six at follow-up 3.

### Hippocampus and amygdala subregion volumes

#### Conditional main effects of alcohol use and family history of alcohol use disorder density

**Fig. 1:**
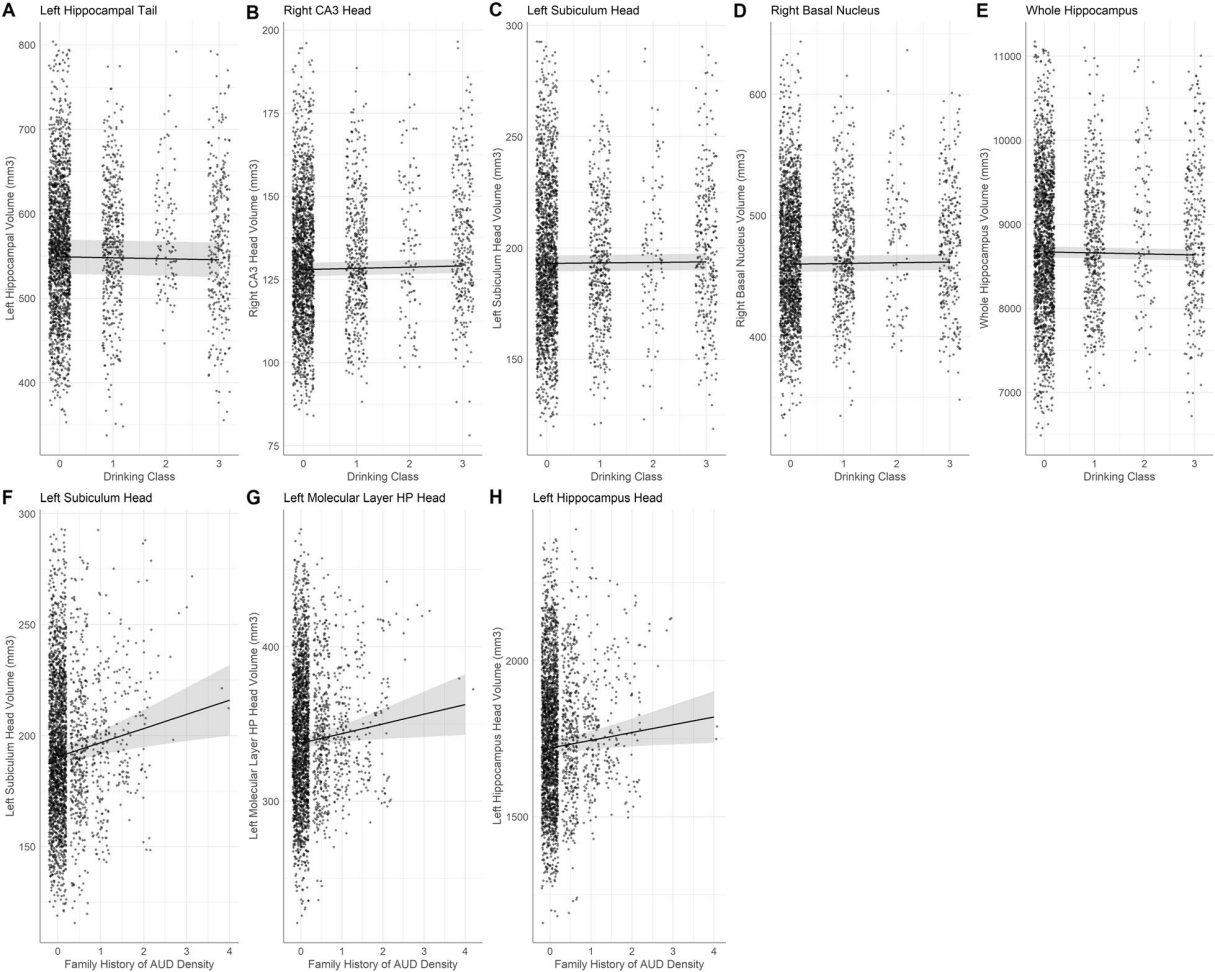
Conditional main effects of drinking class and family history of AUD. Hippocampal subfield and amygdala nuclei volumes predicted by the conditional main effect of drinking class (**A**–**D**) and the conditional main effect of family alcohol use disorder density (**F**–**H**). Whole hippocampus volume predicted by the conditional main effect of drinking class (**E**). Individual data points represent volume at subject scan visits.

Greater alcohol use, indicated by higher drinking class, was associated with smaller hippocampal subfield volume (Fig. 1A–D) in the left hippocampal tail (*β* = −1.2, *p*_FDR_ = 0.048, *R*^2^ adj = 0.24), and larger hippocampal subfield volume in the right CA3 head, (*β* = 0.4, *p*_FDR_ = 0.027, *R*^2^ adj = 0.37), left subiculum head (*β* = 0.7, *p*_FDR_ = 0.046, *R*^2^ adj = 0.27), and right basal nucleus of the amygdala (*β* = 1.3, *p*_FDR_ = 0.040, *R*^2^adj = 0.61). These findings in the right CA3 head and right basal nucleus remained significant even when controlling for cannabis and tobacco drug use. Forty-three percent of the NCANDA sample reported lifetime marijuana use (i.e., one or more times used cannabis).

Greater density scores for family history of AUD were significantly associated with greater left hippocampal subfield volume (Fig. 1F–H) in the subiculum head (*β* = 6.4, *p*_FDR_ = 0.007, *R*^2^ adj = 0.27), molecular layer HP head (*β* = 6.0, *p*_FDR_ = 0.022, *R*^2^ adj = 0.48), whole hippocampus head (*β* = 25.0, *p*_FDR_ = 0.044, *R*^2^ adj = 0.55). These findings in the left subiculum head, left molecular layer HP head, and left whole hippocampus head remained significant even when controlling for cannabis and tobacco drug use.

#### Conditional main effect of early trauma

**Fig. 2:**
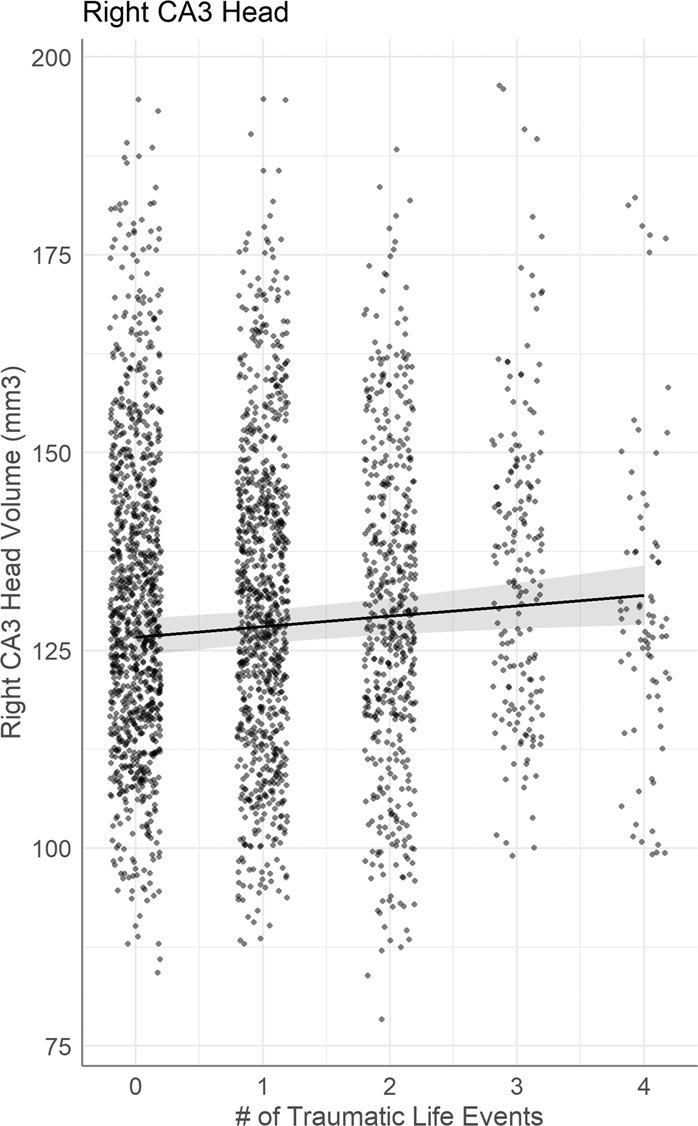
Hippocampal subfield volume predicted by the conditional main effect of lifetime trauma (measured at baseline). Individual data points represent volume measured at subject scan visits.

Higher number of traumatic life events at baseline was significantly associated with larger right hippocampal subfield volume (Fig. 2) in the CA3 head (*β* = 1.3, *p*_FDR_ = 0.041, *R*^2^ adj = 0.37). This finding in the right CA3 head remained significant even when controlling for cannabis and tobacco drug use.

#### Within-person age change by trauma interaction

**Fig. 3:**
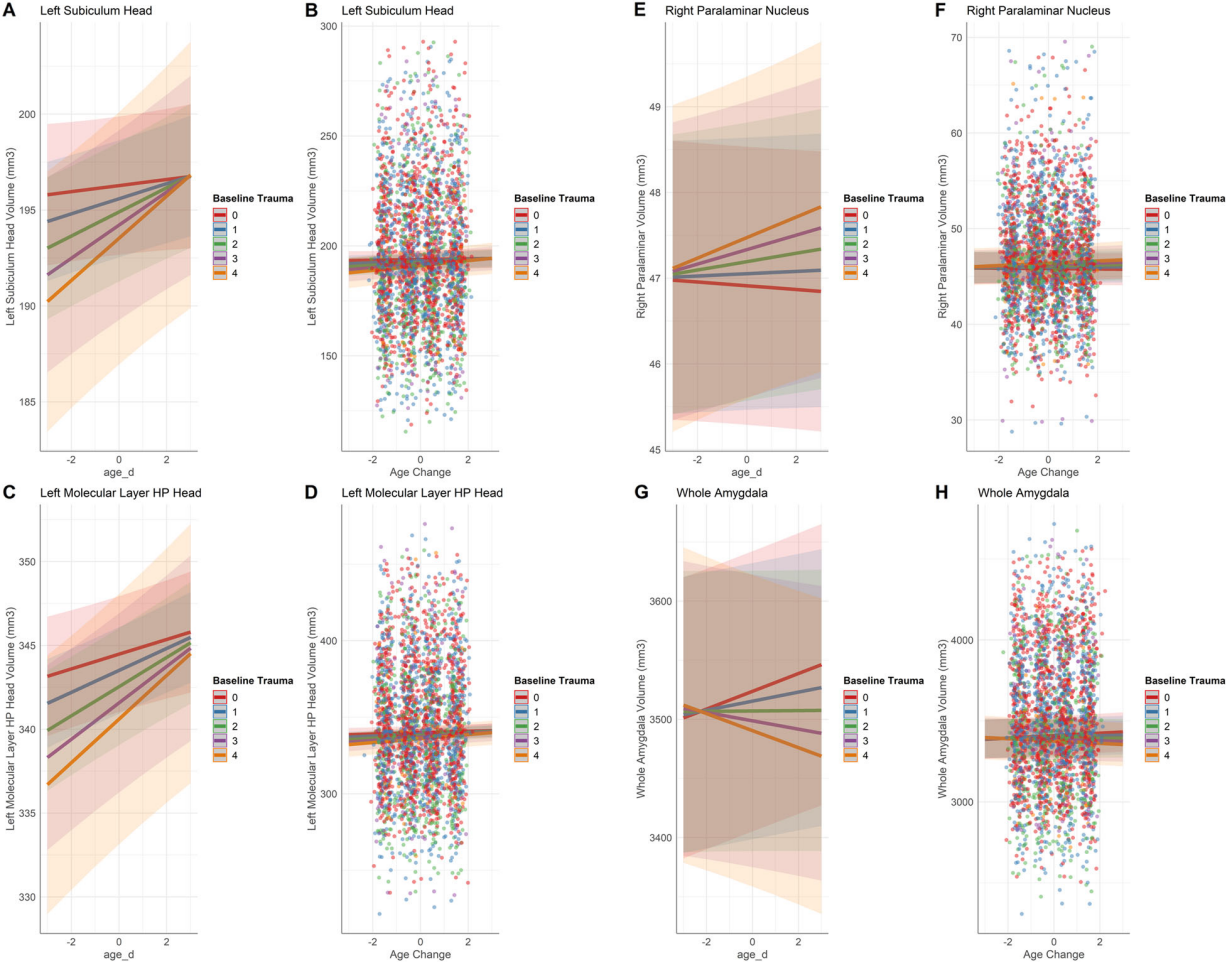
Hippocampal subfield and amygdala nuclei volumes predicted by the interaction between lifetime trauma at baseline and within-person age change (age_d). This interaction is represented for left subiculum head volumes (**A**, **B**), left molecular layer HP head volumes (**C**, **D**), right paralaminar nucleus volumes (**E**, **F**), and whole amygdala volumes (**G**, **H**). Individual data points represent volume measured at subject scan visits. Lines represent the categorical values of the moderator variable, number of lifetime traumatic events at baseline.

The interaction between number of traumatic life events at baseline and within-person change in age was significantly associated with larger left hippocampal subfield volume (Fig. 3A–F) in the subiculum head (*β* = 0.3, *p*_FDR_ = 0.029, *R*^2^ adj = 0.27) and molecular layer HP head (*β* = 0.3, *p*_FDR_ = 0.041, *R*^2^ adj = 0.48), and larger right amygdala nuclei volume in the paralaminar nucleus (*β* = 0.1, *p*_FDR_ = 0.045, *R*^2^ adj = 0.35). That is, regardless of the age cohort, as participants got older, those with more traumatic events showed a steeper decline in these subfield volumes compared to those with fewer traumatic events. These findings in the left subiculum head, left molecular layer HP head, and right paralaminar nucleus remained significant even when controlling for cannabis and tobacco drug use.

#### Drinking class by trauma interaction

**Fig. 4:**
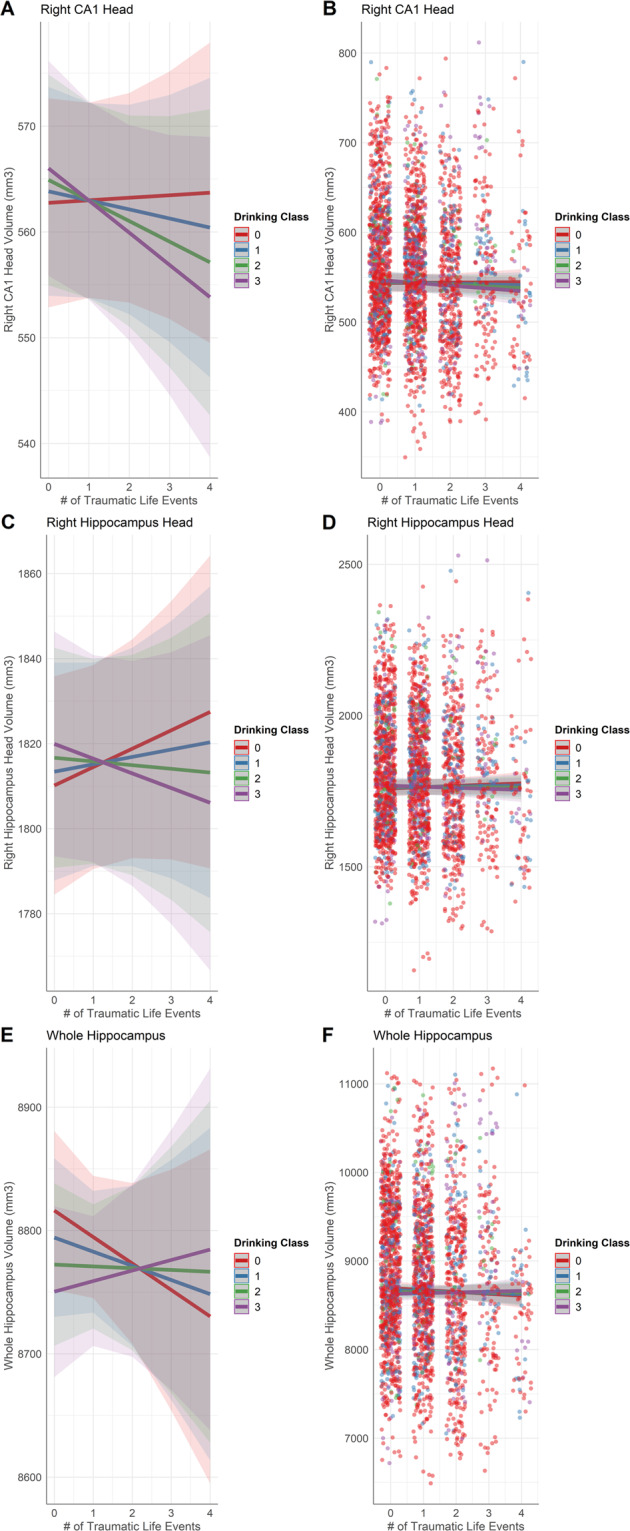
Hippocampal subfield volumes predicted by the interaction between lifetime trauma and drinking class (DrkClass). This interaction is represented for right CA1 head volumes (**A**, **B**), right hippocampus head volumes (**C**, **D**), and whole hippocampus volumes (**E**, **F**). Individual data points represent volume measured at subject scan visits. Lines represent the categorical values of the moderator variable, drinking class.

The interaction between number of traumatic life events at baseline and alcohol use, indicated by drinking class, was significantly associated with smaller right hippocampal subfield volume (Fig. 4A–D) in the CA1 head (*β* = −1.1, *p*_FDR_ = 0.011, *R*^2^ adj = 0.50) and hippocampus head (*β* = −2.6, *p*_FDR_ = 0.025, *R*^2^ adj = 0.57). That is, those with higher drinking classes and more traumatic event exposure exhibited the smallest hippocampal subvolumes. These findings in the right CA1 head and right hippocampus head remained significant even when controlling for cannabis and tobacco drug use.

#### Whole hippocampus and amygdala volumes

Greater alcohol use, indicated by higher drinking class, was associated with slightly smaller whole hippocampus volume (Fig. 1E) (*β* = −12.0, *p*_FDR_ = 0.009, *R*^2^ adj =0.46), and the interaction between number of traumatic life events at baseline and drinking class was significant. This interaction appears to indicate that among those with limited trauma history, higher drinking class was associated with smaller whole hippocampal volume, but this effect diminished with more trauma exposure (Fig. 4E, F), (*β* = 10.0, *p*_FDR_ = 0.032, *R*^2^ adj = 0.46). In the whole amygdala, an interaction between number of traumatic life events at baseline and within-person change in age indicated that older adolescents with greater trauma exposure at baseline had smaller whole amygdala volume (Fig. 3G, F) (*β* = −3.7, *p*_FDR_ = 0.003, *R*^2^ adj = 0.46). These findings in the whole hippocampus and amygdala remained significant even when controlling for cannabis and tobacco drug use. Additionally, higher rates of lifetime marijuana use were significantly associated with smaller whole hippocampus volume (*β* = −0.1, *p*_FDR_ = 0.04, *R*^2^ adj = 0.46).

#### Conditional main effect of lifetime marijuana use

Higher rates of lifetime marijuana use were significantly associated with smaller right hippocampal subfield volume (Supplementary Fig. S4) in the CA1 head (*β* = −0.01, *p*_FDR_ = 0.044, *R*^2^ adj = 0.50), and hippocampal–amygdaloid transition area (HATA) (*β* = −0.002, *p*_FDR_ = 0.048, *R*^2^ adj = 0.29). See Supplementary Tables S4 and S5 for all model statistics reported above.

## Discussion

We investigated the conditional main and interactive effects of alcohol use, and youth trauma reported at baseline, on the structural trajectories of hippocampal subfields and amygdala nuclei across adolescent development in a longitudinal sample. Greater alcohol use was associated with smaller whole hippocampus and left hippocampal tail, but larger right CA3 head and left subiculum volumes. Greater alcohol use was associated with a larger volume of the right basal nucleus of the amygdala. The effect of traumatic life events measured at the baseline visit was associated with larger right CA3 head volume in the hippocampus. Baseline trauma and within-person age change interacted such that younger adolescents with greater trauma exposure at baseline had smaller left hippocampal subfield volumes in the subiculum and molecular layer head. The interaction also revealed that older adolescents with greater trauma exposure at baseline had larger volume in the right paralaminar nucleus of the amygdala. Alcohol use and baseline trauma interacted such that adolescents with greater baseline trauma and higher alcohol use had smaller volumes in the whole amygdala, right CA1 head, and right hippocampal head. These findings provide evidence that adolescent alcohol use and early-life trauma interact to alter growth trajectories of hippocampal and amygdala subregions in complex ways.

### Whole hippocampus and amygdala

Overall, we found that greater alcohol use was associated with reduced whole hippocampus volume. An interaction effect showed that the relationship between whole hippocampal volume and alcohol use depended on trauma exposure at baseline, such that greater alcohol use was associated with smaller hippocampus volume except for adolescents who reported trauma exposure at baseline (Fig. 4E, F). Those with two or more traumatic events at baseline and greater alcohol use showed increased whole hippocampus volume (Fig. 4E, F). In the whole amygdala, an interaction effect showed that as adolescents age, amygdala volume tends to increase for those with no reported exposure to a traumatic event. However, adolescents with greater trauma exposure showed a decrease in whole amygdala volume as they age (Fig. 3G, H).

### Hippocampal subfields

There is recent literature showing reduced right hippocampal CA1 volume is associated with PTSD^51^ and that hippocampal CA1 experiences age-dependent decline of volume in PTSD^52^. An animal model of PTSD shows reduced neurotrophin mRNA levels of BDNF and TrkB are localized to the CA1 subfield of the hippocampus^53^. Interestingly, chronic alcohol exposure in rats produced a similar reduction of mRNA-associated signaling of BDNF and TrkB in the hippocampus^54^. An animal model that used predator stress to model PTSD found similar results marked by BDNF and other neurotrophin changes that produced dramatic neuronal proliferation in the basolateral amygdala, but by contrast showed significant neuronal retraction in the hippocampal CA1, CA3, and dentate gyrus. Opposing patterns with atrophy in the hippocampus and hypertrophy in the basolateral amygdala are well-established responses in animal models of chronic stress^23^. The behavioral disturbances resulting from predator stress were associated with a galaninergic response in hippocampal CA1, but were absent when the behavior was not disrupted^55^. Galanine is a neuropeptide that is linked to anxiety-like behaviors. Interestingly, alcohol exposure during the early neonatal period of development demonstrated reduced glial proliferation in a similar anatomical pattern, which affected CA1, CA3, and dentate gyrus^56^. Thus, extensive converging evidence from exposure to alcohol and to traumatic stress in animal model systems indicates overlapping roles of molecular mediators that affect change in the hippocampus, particularly CA1 and CA3. Unfortunately, a profound lack of evidence in humans is available to inform the impact of early-life trauma, alcohol exposure, and particularly concomitant exposure to early-life trauma and alcohol exposure on hippocampal and amygdala substructures in humans. Our results demonstrate that adolescents with greater baseline trauma and higher alcohol use have smaller volumes in the right CA1 head, and right hippocampal head, which appears to be consistent with the animal literature. However, our results for the amygdala under these conditions are in contrast to the animal literature.

### Clinical relevance of volumetric changes

Longitudinal patterns of subregional volume change during the adolescent period in a significantly larger sample of unaffected controls from NCANDA were concordant with the findings reported in healthy controls by Tamnes and colleagues^57^. Our results demonstrate that early adolescence is a sensitive time period for the neurotoxic effects of alcohol on right amygdala subregions. Functionally connected with the medial prefrontal cortex (mPFC), these substructures are linked with decreased inhibitory control, a key element of addiction, withdrawal, and craving^58^. The smaller volume of the hippocampal tail in adolescents agrees with a cross-sectional study which showed similar findings in adults with AUD who were in several years of remission^59^. While we cannot infer functional change from the present study, the hippocampal tail has connections to the dorsal lateral prefrontal cortex, a brain region implicated in addiction because of its role in inhibition, attention, decision making, learning, and memory^60^.

### Limitations

The present study has several inherent limitations. First, this study did not evaluate the cognitive impact of hippocampus and amygdala volume change due to alcohol use or trauma exposure. An important next step is to explore whether the presence and duration of hippocampal decline are associated with functional cognitive deficits in this sample. Animal studies demonstrate that the upregulation of neuroimmune signaling may link heavy alcohol use to hippocampal decline^18^. Despite significant alterations in the growth trajectories for these substructures, there is reason to believe based on animal studies that the impact of heavy alcohol use may not be long-lasting. Also, studies in adults have shown that abstinence can have salutary effects^40^. Future investigations using the existing NCANDA sample may be designed to understand the effects of dissentients from binge drinking on the young adult brain. See Supplementary Materials for a discussion on the neurodevelopmental trajectories for non-drinkers in this sample (i.e., those who never reported engaging in regular alcohol consumption), and Supplementary Fig. S5 for these subregion trajectories plotted by hemisphere.

Second, our analyses did not control for the presence of developmental psychopathology. Other NCANDA investigators are examining diagnostic information from the sample, and this is beyond the scope of the current manuscript. Criteria for heavy alcohol use risk were: (1) Initiation of alcohol use before age 15; (2) Positive family history of AUD; (3) One or more externalizing symptoms (e.g., conduct disorder); or (4) Two or more internalizing symptoms (e.g., depression or anxiety). Roughly 50% of the sample met high-risk criteria^38^. Third, the hippocampal subfields and amygdala nuclei segmentation used in Freesurfer v6.0 relies on atlas priors. The ultra-high-resolution images (100-μm^3^ isotropic) used in atlas construction have sufficient contrast to demarcate boundaries of nuclei with high confidence^49^. The segmentation of 1-mm isotropic scans depends on this atlas, particularly when the algorithm has insufficient information for labeling from image contrast. Across the cohort, an unintended consequence is that each subject’s volume measurement is more similar to every other subject than if ultra-high-resolution technology was available for in vivo scanning in the present sample. Artificially low variance means that group differences will manifest as smaller effect sizes than the true effect size. However, a lower bound on this reduced variability is imposed by the whole amygdala and whole hippocampal segmentation, which is capable of being segmented with fairly high fidelity at the scanning resolution we used. In other words, the variability in nuclei segmentation will be proportional to the variability in the whole amygdala or whole hippocampal segmentation even in the worst-case scenario that subregion segmentation is 100% atlas driven. Thus, smaller substructures like the GC-DG, CA4, and molecular layer should be interpreted with caution. Fourth, while the NCANDA investigation did collect information from adolescents about memory blackouts resulting from alcohol use, only a small proportion of the sample endorsed blackouts at follow-up 3 visits (13%). The lifetime number of blackouts at the 3-year follow-up ranged from 1 to 20 with a mean of 2.43 (SD 2.99). We did not have enough power to run sub-analyses on the participants who reported blackouts, which were few. Lastly, this study relied on the first 4 years of acquired NCANDA data. Additional timepoints from the ongoing study will expand the number of participants within each developmental cohort and permit the assessment of long-term sequelae in the brain.

## Conclusion

We observed unique effects of trauma, as it interacts with alcohol use and age, in the developing bilateral hippocampus and bilateral amygdala. Our results provide initial evidence that heavy alcohol use alters adolescent hippocampal subfields and amygdala nuclei volumes, and these changes during development may be dependent upon youth trauma exposure.

## Supplementary information

Supplemental Materials

Figure S1

Figure S2

Figure S3

Figure S4

Figure S5

Table S1

Table S2

Table S3

Table S4

Table S5
