# Supplementary material for: Volumetric trajectories of hippocampal subfields and amygdala nuclei influenced by adolescent alcohol use and lifetime trauma
Source: Transl Psychiatry. 2021 Mar 2;11:154. doi: 10.1038/s41398-021-01275-0 (PMC7925562; doi:10.1038/s41398-021-01275-0)
Supplement: Supplementary file 1 — Supplemental Materials [file 41398_2021_1275_MOESM1_ESM.docx]

**Supplementary Materials**

# Materials and Methods

## Assessment of Trauma

Measures of DSM Criterion A traumas and PTSD symptoms were collected at baseline as part of the baseline NCANDA interview assessment from the youth, and parent with the Computerized Semi-Structured Assessment for the Genetics of Alcoholism (Bucholz et al., 1994; Hesselbrock, Easton, Bucholz, Schuckit, & Hesselbrock, 2002). The SSAGA was modified for adolescents and includes an interview assessment of DSM-IV and 5 Axis I disorders, DSM Criterion A traumas and PTSD interview questions (S. A. Brown et al., 2015). Of the 831 NCANDA subjects, 830 parents and/or youth completed the trauma interview. Number of traumatic events reported at baseline were calculated as the sum of positive baseline endorsements by either parent or youth of one or more of the DSM Criterion A traumas. Frequencies of individual DSM Criterion A traumas for the sample are reported in Table S2. Reporting baseline traumatic events in the NCANDA sample was common with 62% of the sample reporting at least one trauma. These data are similar to the National Comorbidity Survey Replication Adolescent Supplement (NCSRAS), which reported that 61.8% of 6483 adolescents age 13 to 17 years reported a traumatic event (McLaughlin et al., 2013). Further information about the baseline traumas experienced in NCANDA was previously published (De Bellis et al., 2020). When NCANDA participants are age 18, we collect more detailed trauma information including the childhood trauma questionnaire for measures of maltreatment.

## Intra-class Correlations (ICC)

The intra-class correlation coefficient (ICC) for each subfield was calculated to measure within-subject variability relative to between-subject variability in volume over time. A portion of the sample (n=488) who completed all four yearly scan sessions were included in ICC calculation to assess agreement in segmentation for individuals across time. ICC calculation was also performed across the four scan sessions, by site. All analyses were modeled by a one-way random effects model, conducted in the statistical program, R version 4.0.0, using the irr package for ICC analysis.

## Statistical Modeling

NCANDA’s accelerated longitudinal design (e.g. cohort-sequential design), allows us to consider both the within-subject and within-cohort structural brain changes over the course of the study. Within-person age change represented the difference between a subject’s age at each scan and their mean age across individual timepoints. Cohort age represented the difference between a subject’s mean age across visits and the mean age of the entire sample across timepoints, thus centering cohort age at the sample mean. Each participant’s cohort age remained constant across timepoints.

Following previously implemented approaches for structured multi-cohort longitudinal designs, we modeled the developmental trajectories of hippocampal subfields and amygdala nuclei using a mixed-effects approach (Thompson, Hallmayer, & O’Hara, 2011). In this design, random effects accounted for the within-subject covariance across time. In all models, NCANDA site and participant identity were included as random intercepts. Within-person age change, cohort age, whole hippocampal or amygdala volume, sex, race, socioeconomic status (SES), drinking class, family history of AUD density (Rice et al., 1995), and cumulative lifetime trauma at baseline were included as covariates for conditional likelihood. Covariates of race, sex, SES, trauma, and family history that were assessed at baseline were modeled as stable variables, which did not vary across time. This was achieved by repeating baseline values across timepoints per subject.

# Results & Discussion

## Intra-class Correlations (ICC)

Reliability was moderate to high among subregions across all sites, ranging from 0.56 to 0.98. A majority of subregions (87.5%) showed ICC values in the good-excellent range, above 0.75, while only 12.5% of the subregions had moderate reliability, below 0.75 and above 0.5. There was more variability in ICC values when separated by site, but a majority of subregions showed ICC values in the good-excellent range at the Pittsburgh (79.7%), Duke (82.8%), SRI (81.3%), OHSU (75%), and UCSD (84.4%) sites. Problematic regions with poor reliability, ICC below 0.5, were the left basal and right central nuclei for the Pittsburgh site (n=69), right hippocampal tail and right AAA for the SRI site (n=63), and left AAA and right central nucleus for the OHSU site (n=106). Small sample size may have contributed to lower reliability for these regions by site. The number of subjects who completed all four scan sessions at each site are reported alongside the ICC values for the entire sample (n=488) in Figure S3. Brown and colleagues (E. M. Brown et al., 2020) assessed the test-retest reliability of the Freesurfer v6.0 hippocampal segmentation approach, and this method was found to have high reliability across scan sessions that were acquired seven to fifty days apart. Reduced reliability in the current NCANDA sample may be due to greater variation within-subject during neurodevelopment, given that scans were performed annually.

## Sex Effects

Males in the NCANDA sample (n=391) were characterized by increased whole hippocampal volume (*β*=244,*p_FDR_*<.001, *R*^2^ adj =0.46) and whole amygdala volume (*β*=229,*p_FDR_*<.001, *R*^2^ adj =0.46), as well as increased hippocampal subfield and amygdala nuclei volumes (Table S4 and S5) in the left and right hippocampal tail, left and right subiculum body and head, right CA1 body, left and right CA1 head, left and right CA3 head, right CA4 body, left CA4 head, left and right presubiculum body and head, left and right parasubiculum, left and right molecular layer HP body and head, left GCMLDG body and head, left and right fimbria, left and right HATA, left and right whole hippocampus body and head, left and right whole hippocampus, left and right lateral nucleus, left and right basal nucleus, left and right accessory basal nucleus, left and right anterior amygdaloid area (AAA), left and right central nucleus, left and right cortical nucleus, left and right corticoamygdaloid transition area, left and right medial nucleus, left and right paralaminar nucleus, and left and right whole amygdala.

## Socioeconomic Status

Higher SES at baseline, measured as parental years of education, was associated with increased whole hippocampal volume (*β*=24,*p_FDR_*=.014, *R*^2^ adj =0.46) and increased hippocampal subfield and amygdala nuclei volumes (Table S4 and S5) in the left and right subiculum head, left CA1 head, left CA3 head, left CA4 head, left presubiculum head, left and right parasubiculum, left molecular layer HP head, left GCMLDG head, left whole hippocampus body, left and right whole hippocampus head, left whole hippocampus, left and right basal nuclei, left accessory basal nucleus, left central nucleus, and left and right paralaminar nuclei. Children from low-income settings experience atypical structural brain maturation and development in total gray matter, prefrontal cortex, temporal lobe structures, and hippocampus (Hair, Hanson, Wolfe, & Pollak, 2015). Conversely, privileged SES appears to be a protective factor that benefits neurodevelopment, with greater cognitive performance even when neural differences are not evident (Hackman & Farah, 2009). The impact of SES, particularly in the context of adolescent drinking is an important avenue of study that should be explored in future studies.

## Developmental trajectories for Non-drinkers

There are a number of subjects (n=408) who never reported engaging in regular alcohol consumption, as measured by the Cahalan drinking class scale (Figure S1). To better understand typical neurodevelopmental trajectories of hippocampal and amygdala subregions, we ran a follow-up analysis in control subjects, adolescents who never engaged in regular drinking during the study. We found that age change (i.e. mean-centered within-subject age), while not associated with change in whole hippocampus or amygdala volume, was associated with increased volume in the right hippocampal tail, left subiculum body, right CA3 head, left CA4 body, right CA4 head, right presubiculum head, right molecular layer HP head, left fimbria, left whole hippocampus body, right whole hippocampus head, right basal nucleus, right AAA, right central nucleus, right cortical nucleus, and right whole amygdala. Age index (i.e. age mean-centered on the sample mean at baseline), while not associated with change in whole hippocampus or amygdala volume, was associated with increased volume in the left fissure and decreased volume in the right parasubiculum. The interaction between age index and age change significantly predicted whole amygdala volume, right whole hippocampal volume, and subregion volume in the right basal nucleus and left medial nucleus. These findings seem to support the conclusions made in the entire NCANDA sample (n=803) of drinkers and non-drinkers, such that neurodevelopmental trajectories of the subregions of the hippocampus and amygdala are distinct from those of their whole structures, and that there are distinct patterns based on laterality (Figure S5).

**References**

1. Bucholz KK, Cadoret R, Cloninger CR, Dinwiddie SH, Hesselbrock VM, Nurnberger JI, et al. A new, semi-structured psychiatric interview for use in genetic linkage studies: a report on the reliability of the SSAGA. J Stud Alcohol. 1994;55:149–158.

2. Hesselbrock M, Easton C, Bucholz KK, Schuckit M, Hesselbrock V. A validity study of the SSAGA-a comparison with the SCAN. Addiction. 2002;94:1361–1370.

3. Brown SA, Brumback T, Tomlinson K, Cummins K, Thompson WK, Nagel BJ, et al. The National Consortium on Alcohol and NeuroDevelopment in Adolescence (NCANDA): A Multisite Study of Adolescent Development and Substance Use. J Stud Alcohol Drugs. 2015;76:895–908.

4. McLaughlin KA, Koenen KC, Hill ED, Petukhova M, Sampson NA, Zaslavsky AM, et al. Trauma exposure and posttraumatic stress disorder in a national sample of adolescents. J Am Acad Child Adolesc Psychiatry. 2013;52:815-830.e14.

5. De Bellis MD, Nooner KB, Brumback T, Clark DB, Tapert SF, Brown SA. Posttraumatic Stress Symptoms Predict Transition to Future Adolescent and Young Adult Moderate to Heavy Drinking in the NCANDA Sample. Curr Addict Reports. 2020;7:99–107.

6. Thompson WK, Hallmayer J, O’Hara R. Design considerations for characterizing psychiatric trajectories across the life span: Application to effects of APOE-ε4 on cerebral cortical thickness in Alzheimer’s disease. Am J Psychiatry. 2011;168:894–903.

7. Rice JP, Reich T, Bucholz KK, Neuman RJ, Fishman R, Rochberg N, et al. Comparison of Direct Interview and Family History Diagnoses of Alcohol Dependence. Alcohol Clin Exp Res. 1995;19:1018–1023.

8. Brown EM, Pierce ME, Clark DC, Fischl BR, Iglesias JE, Milberg WP, et al. Test-retest reliability of FreeSurfer automated hippocampal subfield segmentation within and across scanners. Neuroimage. 2020;210:116563.

9. Hair NL, Hanson JL, Wolfe BL, Pollak SD. Association of child poverty, brain development, and academic achievement. JAMA Pediatr. 2015;169:822–829.

10. Hackman DA, Farah MJ. Socioeconomic status and the developing brain. Trends Cogn Sci. 2009;13:65–73.

**Supplementary Table and Figure Legends**

*Supplementary Table S1*. Demographic Characteristics by Site and Timepoint (Baseline, Follow-Up 1, Follow-Up 2, Follow-Up 3). Pitt = University of Pittsburgh Medical Center; SRI = SRI International; Duke = Duke University Medical Center; OHSU = Oregon Health and Science University; UCSD = University of California at San Diego; ^1^ Statistics presented: mean (SD) [minimum-maximum]; n (%) ^2^ Statistical tests performed: Kruskal-Wallis test; chi-square test of independence. Note – although SES and trauma variables are shown across timepoints, these are fixed and time-invariant variables; differences in descriptive statistics across time reflect dropout of scans from analysis due to missing or unusable data.

*Supplementary Table S2.*DSM-4 & 5 Type A Traumas reported in the baseline NCANDA sample. Table S2 shows the reports of traumas endorsed by the parent and/or youth, on the Computerized Semi-Structured Assessment for the Genetics of Alcoholism (SSAGA) interview in the baseline NCANDA sample (N=831). This table was adopted from De Bellis et al. 2020. The SSAGA is a top down interview (Hesselbrock et al.,1999; Bucholz et al., 1994). The SSAGA was modified for adolescents and includes an interview assessment of DSM-IV and 5 Axis I disorders, DSM type A traumas and PTSD interview questions (Brown et al., 2015). The mean number of traumatic events was 1.15 (+1.21) ranging from 0-6. 62% of the youth sample experienced at least one DSM type A traumatic event. Experiencing a traumatic event in childhood and adolescence is common. The NCANDA sample is similar to other population samples which reported similar rates such as the National Comorbidity Survey Replication Adolescent Supplement (NCSRAS) (McLaughlin  et  al., 2013).

De Bellis MD., Nooner K.B., Brumback, T., Clark, D.B., Brown, S.A. Posttraumatic Stress Symptoms Predicts Transition to Future Adolescent and Young Adult Moderate to Heavy Drinking in the NCANDA Sample. Current Addiction Reports, 2020, 7: 99– 107.

Hesselbrock M, Easton C, Bucholz KK, Schuckit M, Hesselbrock V. A validity study of the SSAGA--a comparison with the SCAN. Addiction. 1999;94(9):1361-70.

Bucholz KK, Cadoret R, Cloninger CR, Dinwiddie SH, Hesselbrock VM, Nurnberger JI, et al. A new, semi-structured psychiatric interview for use in genetic linkage studies. Journal of Studies on Alcohol. 1994;55:149-58.

Brown SA, Brumback T, Tomlinson K, Cummins K, Thompson WK, Nagel BJ, et al. The National Consortium on Alcohol and NeuroDevelopment in Adolescence (NCANDA): Characterizing risk and resilience for alcohol use in adolescents. Journal of Studies on Alcohol and Drugs. 2015;76:895-908

McLaughlin KA, Koenen KC, Hill ED, Petukhova M, Sampson NA, Zaslavsky AM, et al. Trauma Exposure and Posttraumatic Stress Disorder in a National Sample of Adolescents. Journal of the American Academy of Child & Adolescent Psychiatry. 2013;52(8):815-30.e14.

*Supplementary Table S3*. Descriptive statistics for hippocampal subfield and amygdala nuclei volumes.

*Supplementary Table S4*. Effect sizes for model predictors on whole hippocampus and amygdala volumes. Generalized additive mixed models (GAMM) output from gamm4 package in R. ^1^ CI = Confidence Interval; ^2^False discovery rate correction for multiple testing.

*Supplementary Table S5.* Effect sizes for model predictors on hippocampal subfield and amygdala nuclei volumes. Generalized additive mixed models (GAMM) output from gamm4 package in R. ^1^ CI = Confidence Interval; ^2^False discovery rate correction for multiple testing.

*Figure S1.*Modified Cahalan inventory for drinking class.

*Figure S2.*Anterior (left) and sagittal (right) 3D visualization of bilateral hippocampal subfields and amygdala nuclei. Segmented using Freesurfer longitudinal stream V6.0 and rendered using 3D Slicer V10.4.2. Right cortex shown as a wireframe for illustrative purposes. The GCMLDG in the Freesurfer v6.0 hippocampal segmentation is absorbed by the CA4 region and is therefore not visible in the figure. Freesurfer colormap can be found here: <https://surfer.nmr.mgh.harvard.edu/fswiki/FsTutorial/AnatomicalROI/FreeSurferColorLUT>

*Figure S3.*Correlation matrix for hippocampal subfield and amygdala nuclei volumes.

*Figure S4.*Hippocampal subfields predicted by the conditional main effect of lifetime marijuana use (days used). Individual data points represent volume at subject scan visits.

*Figure S5.*Developmental trajectories for bilateral hippocampal subfields and amygdalar nuclei for drinkers and non-drinkers (I.e., adolescents who never endorsed alcohol use during the course of the study).
