# Supplementary figures and images for: Volumetric trajectories of hippocampal subfields and amygdala nuclei influenced by adolescent alcohol use and lifetime trauma
Source: Transl Psychiatry. 2021 Mar 2;11:154. doi: 10.1038/s41398-021-01275-0 (PMC7925562; doi:10.1038/s41398-021-01275-0)

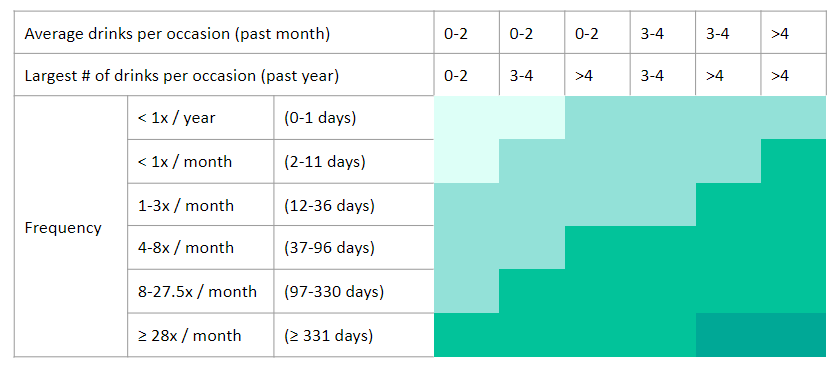

Supplement: Supplementary file 2 — Figure S1 [file 41398_2021_1275_MOESM2_ESM.png]

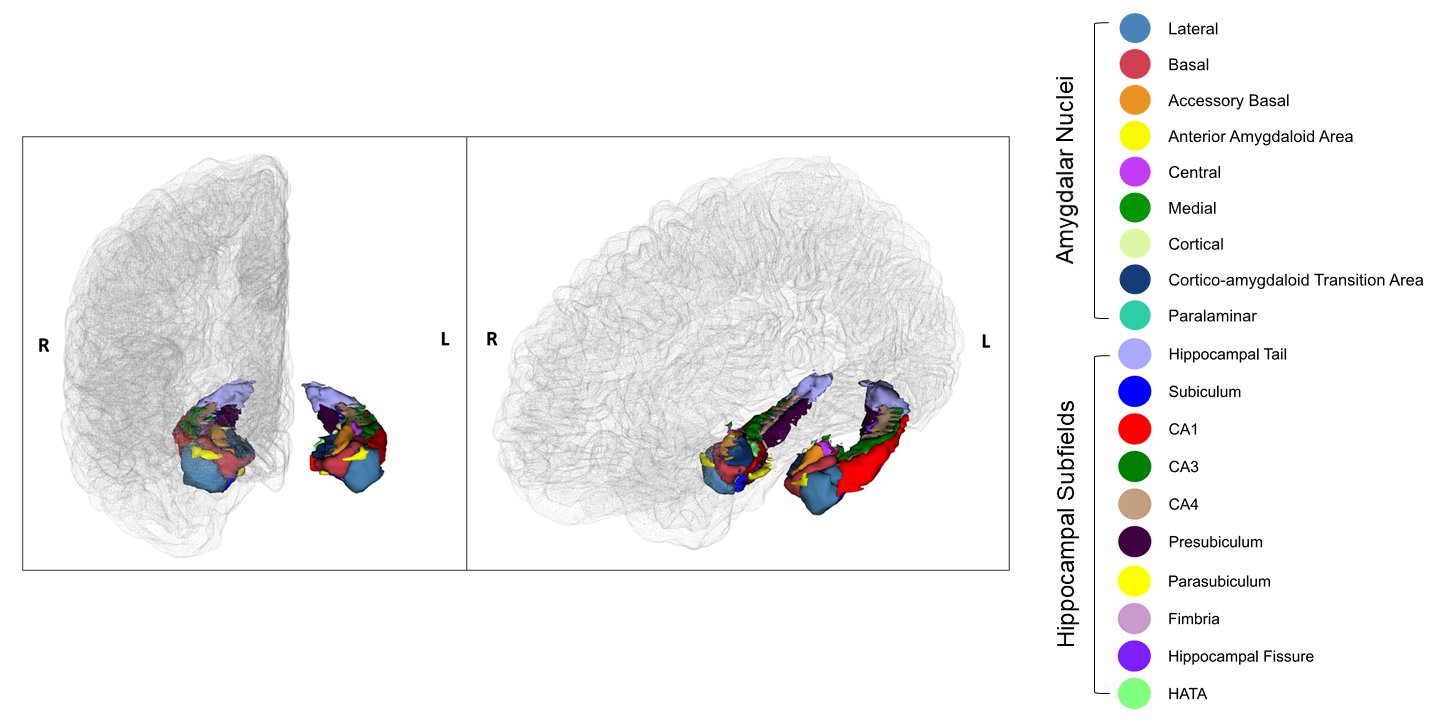

Supplement: Supplementary file 3 — Figure S2 [file 41398_2021_1275_MOESM3_ESM.tif]

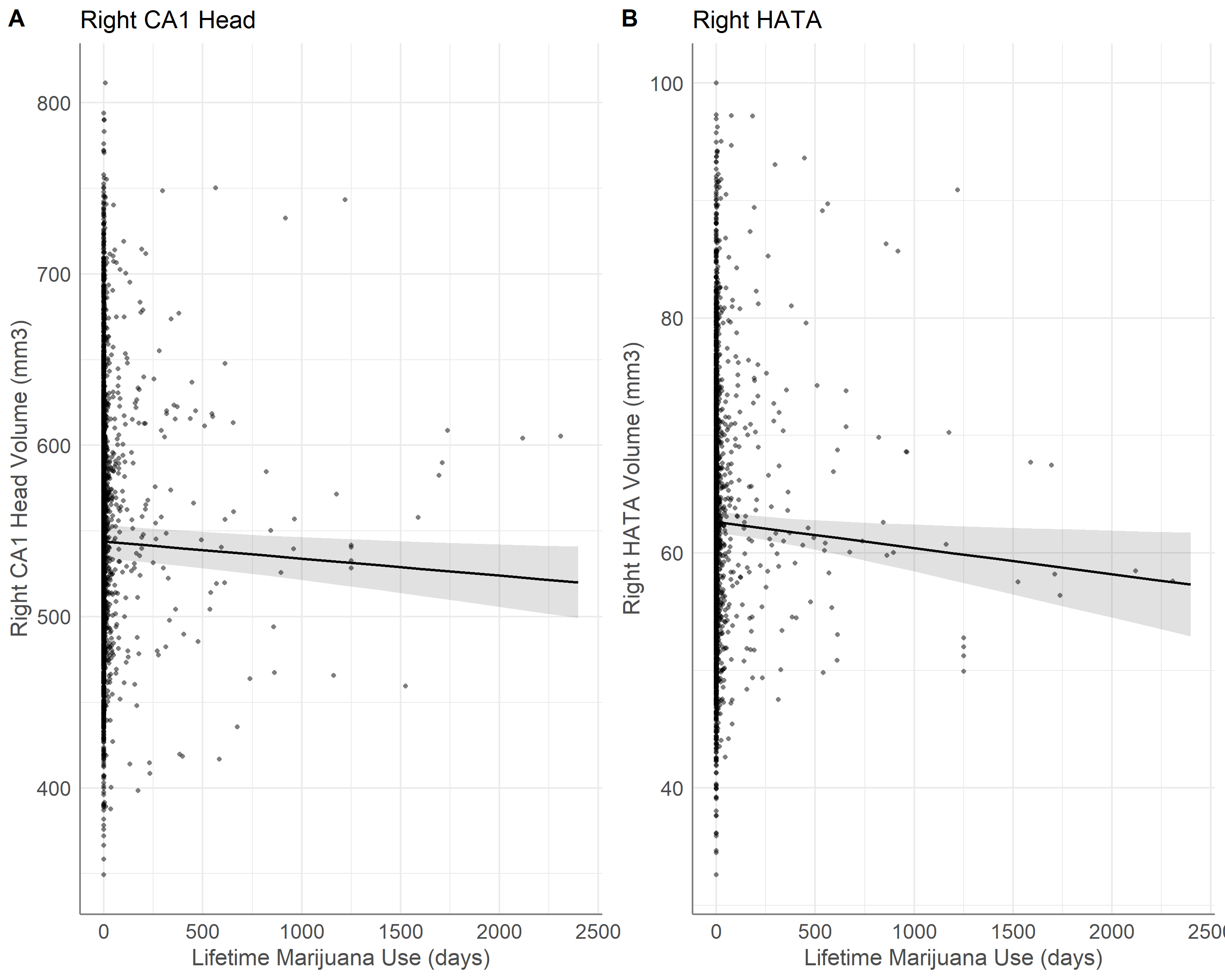

Supplement: Supplementary file 5 — Figure S4 [file 41398_2021_1275_MOESM5_ESM.tif]

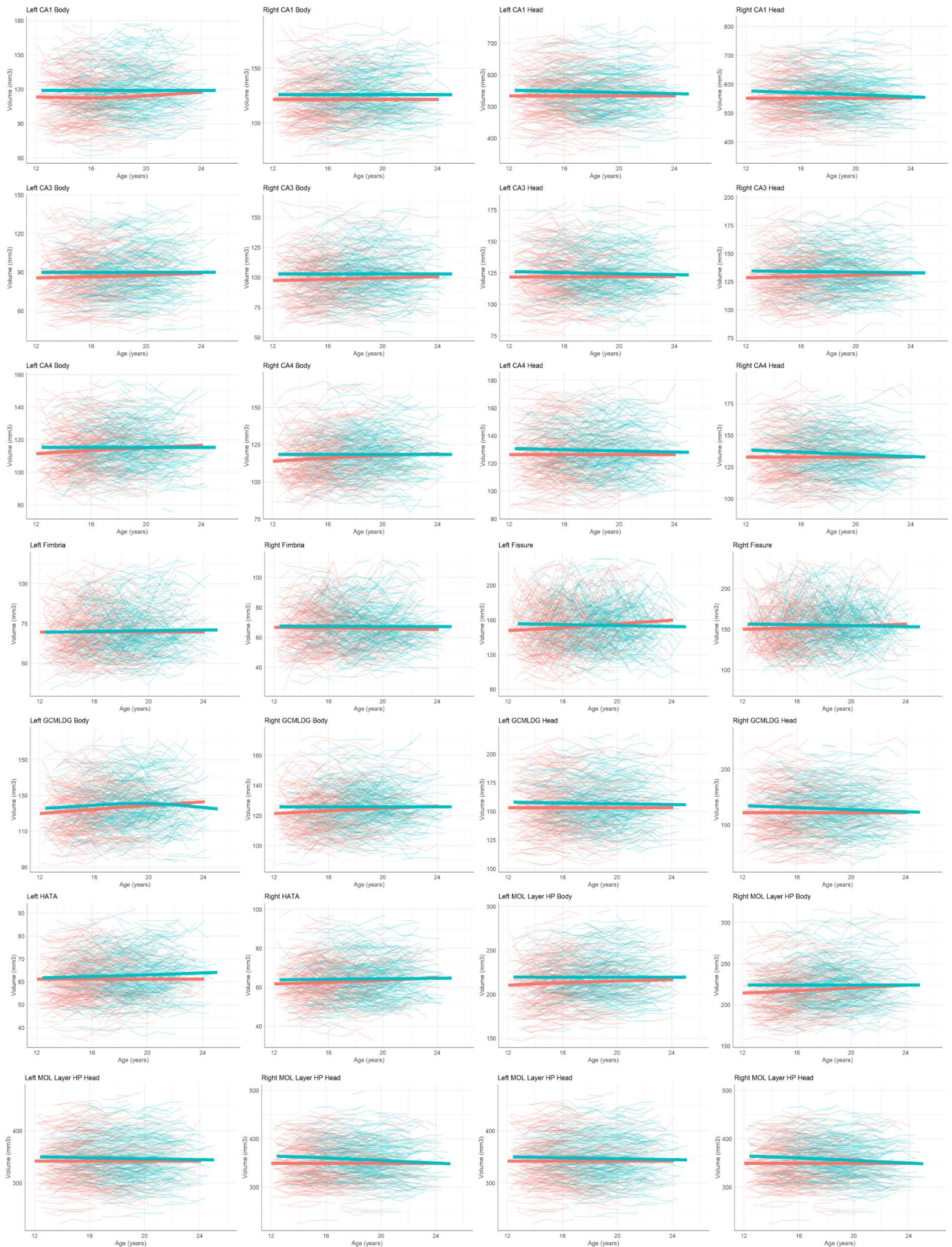

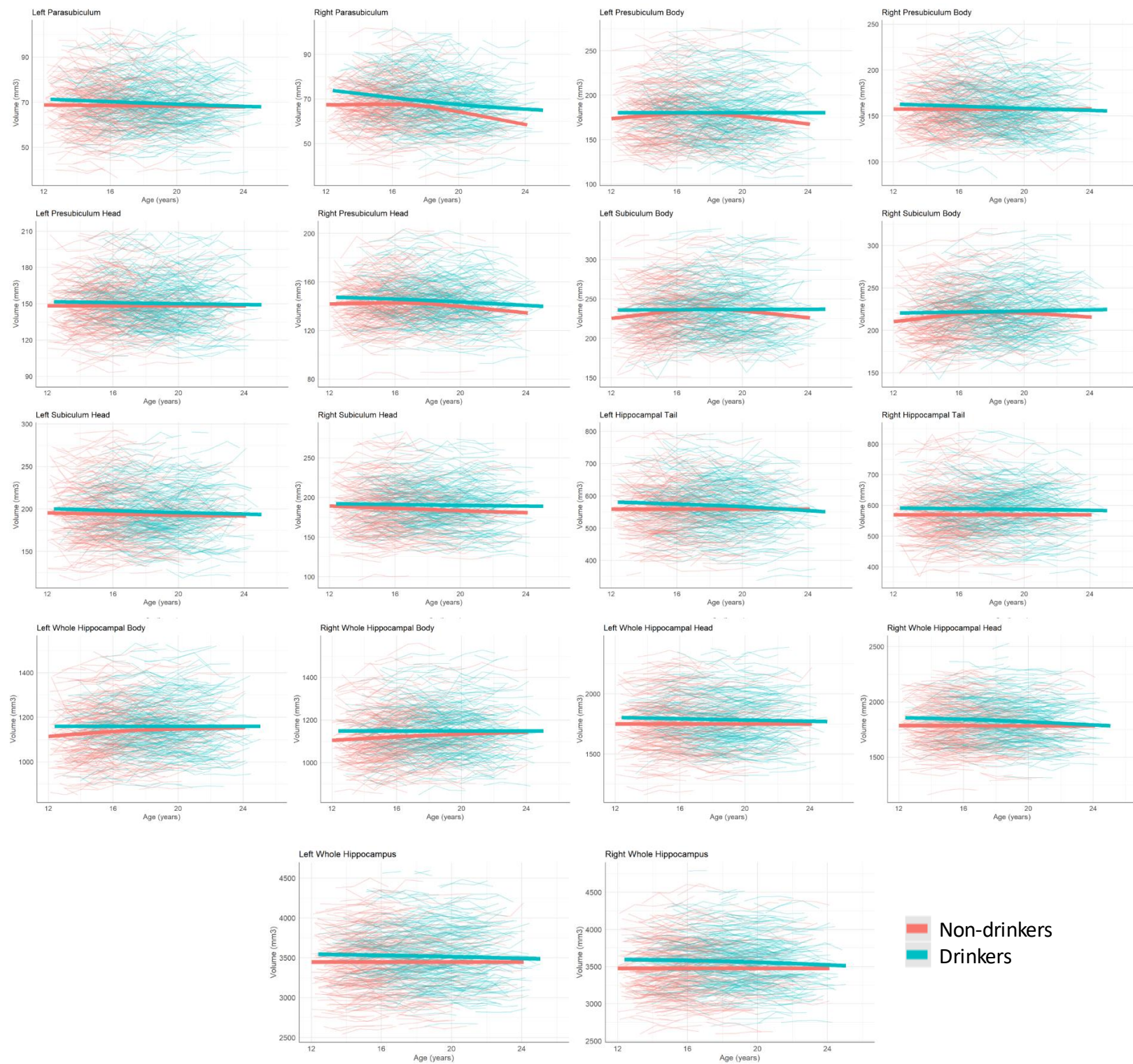

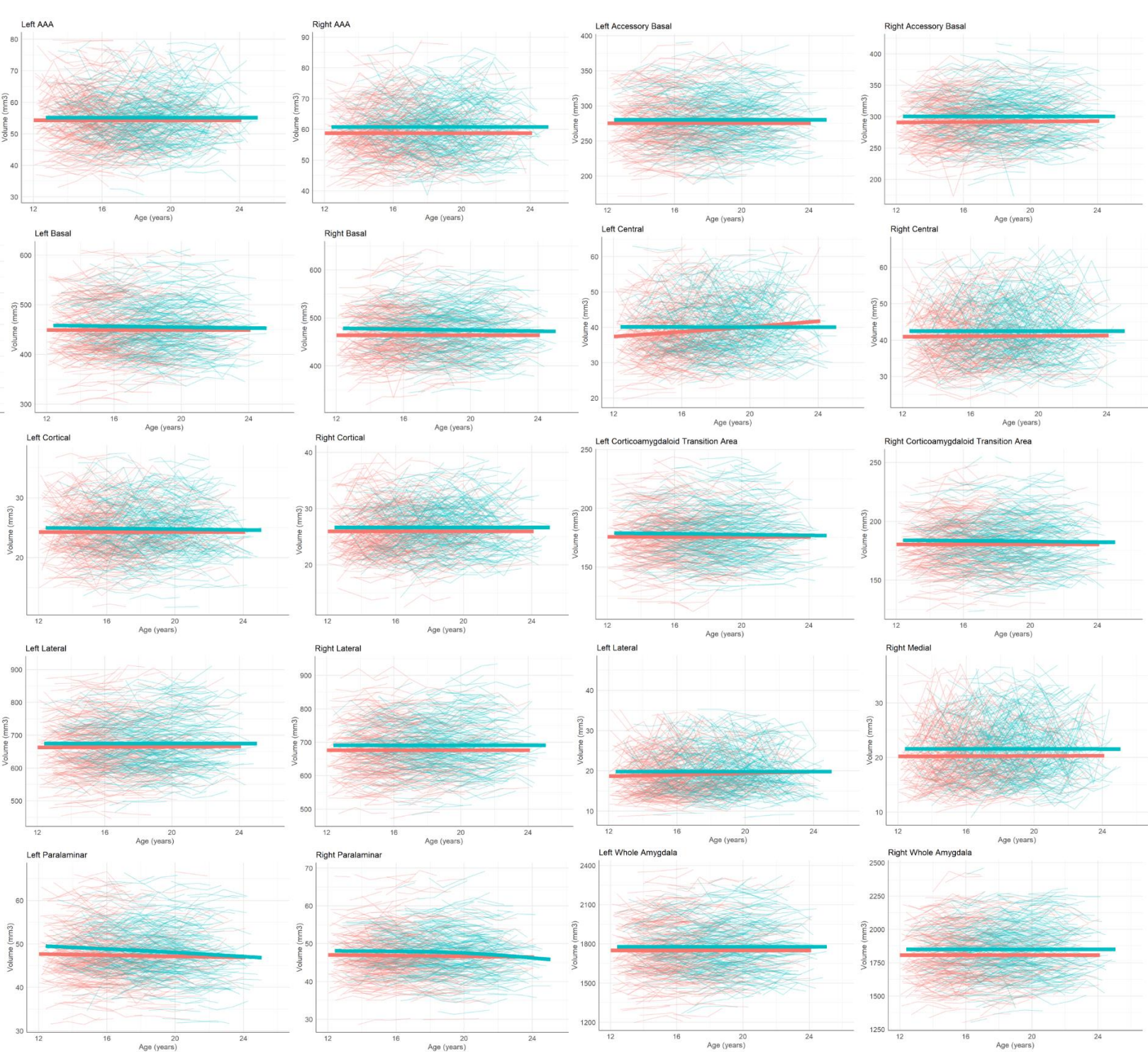

Non-drinkers  
Drinkers

Supplement: Supplementary file 6 — Figure S5 [file 41398_2021_1275_MOESM6_ESM.pdf]
