# Supplementary material for: Volumetric trajectories of hippocampal subfields and amygdala nuclei influenced by adolescent alcohol use and lifetime trauma
Source: Transl Psychiatry. 2021 Mar 2;11:154. doi: 10.1038/s41398-021-01275-0 (PMC7925562; doi:10.1038/s41398-021-01275-0)
Supplement: Supplementary file 7 — Table S1 [file 41398_2021_1275_MOESM7_ESM.pdf]

Baseline	Overall[1] n = 803	Pitt[1] n = 120	SRI[1] n = 157	Duke[1] n = 169	OHSU[1] n = 147	UCSD[1] n = 210	p-value[2]
Age at Scan (years)	16.21 (2.52) [12.02-21.96]	16.93 (2.59) [12.27 -21.96]	15.48 (2.04) [12.02 -21.28]	15.91 (2.46) [12.04 -21.61]	16.40 (2.66) [12.18 -21.42]	16.47 (2.60) [12.04 -21.95]	**<0.001**
Sex							0.6
F	412 (51%)	69 (57%)	78 (50%)	89 (53%)	75 (51%)	101 (48%)	
M	391 (49%)	51 (42%)	79 (50%)	80 (47%)	72 (49%)	109 (52%)	
Race							
African-American/Black	96 (12%)	24 (20%)	3 (1.9%)	57 (34%)	1 (0.7%)	11 (5.2%)	
African-American & Caucasian	9 (1.1%)	1 (0.8%)	0 (0%)	4 (2.4%)	1 (0.7%)	3 (1.4%)	
Asian	64 (8.0%)	0 (0%)	20 (13%)	8 (4.7%)	13 (8.8%)	23 (11%)	
Asian Pacific Islander	1 (0.1%)	0 (0%)	0 (0%)	1 (0.6%)	0 (0%)	0 (0%)	
Asian & White	21 (2.6%)	2 (1.7%)	8 (5.1%)	1 (0.6%)	4 (2.7%)	6 (2.9%)	
Caucasian/White	600 (75%)	93 (78%)	123 (78%)	97 (57%)	124 (84%)	163 (78%)	
Native American/American Indian	3 (0.4%)	0 (0%)	1 (0.6%)	0 (0%)	1 (0.7%)	1 (0.5%)	
Native American & Caucasian	2 (0.2%)	0 (0%)	0 (0%)	0 (0%)	0 (0%)	2 (1.0%)	
None	2 (0.2%)	0 (0%)	2 (1.3%)	0 (0%)	0 (0%)	0 (0%)	
Pacific Islander	4 (0.5%)	0 (0%)	0 (0%)	1 (0.6%)	3 (2.0%)	0 (0%)	
Pacific Islander & Caucasian	1 (0.1%)	0 (0%)	0 (0%)	0 (0%)	0 (0%)	1 (0.5%)	
SES	16.79 (2.49) [6.00-20.00]	16.55 (2.34) [11.00 -20.00]	17.56 (2.30) [6.00 -20.00]	17.00 (2.58) [8.00 -20.00]	16.66 (2.12) [8.00 -20.00]	16.29 (2.73) [6.00 -20.00]	**<0.001**
Family AUD Density							
0	601 (75%)	93 (78%)	119 (76%)	143 (85%)	103 (70%)	143 (68%)	
0.5	117 (15%)	16 (13%)	20 (13%)	19 (11%)	29 (20%)	33 (16%)	
1	46 (5.7%)	8 (6.7%)	7 (4.5%)	4 (2.4%)	10 (6.8%)	17 (8.1%)	
1.5	22 (2.7%)	3 (2.5%)	4 (2.5%)	3 (1.8%)	2 (1.4%)	10 (4.8%)	
2	14 (1.7%)	0 (0%)	5 (3.2%)	0 (0%)	2 (1.4%)	7 (3.3%)	
2.5	1 (0.1%)	0 (0%)	0 (0%)	0 (0%)	1 (0.7%)	0 (0%)	
3	1 (0.1%)	0 (0%)	1 (0.6%)	0 (0%)	0 (0%)	0 (0%)	

	4	1 (0.1%)	0 (0%)	1 (0.6%)	0 (0%)	0 (0%)	0 (0%)	
Drinking Class (Cahalan scale)								
	0	642 (80%)	89 (74%)	123 (78%)	137 (81%)	127 (86%)	166 (79%)	
	1	110 (14%)	18 (15%)	30 (19%)	12 (7.1%)	15 (10%)	35 (17%)	
	2	12 (1.5%)	2 (1.7%)	1 (0.6%)	6 (3.6%)	2 (1.4%)	1 (0.5%)	
	3	39 (4.9%)	11 (9.2%)	3 (1.9%)	14 (8.3%)	3 (2.0%)	8 (3.8%)	
Baseline Trauma (# of events)								
	0	305 (38%)	58 (48%)	92 (59%)	29 (17%)	49 (33%)	77 (37%)	
	1	265 (33%)	36 (30%)	37 (24%)	65 (38%)	48 (33%)	79 (38%)	
	2	154 (19%)	14 (12%)	21 (13%)	51 (30%)	28 (19%)	40 (19%)	
	3	56 (7.0%)	8 (6.7%)	4 (2.5%)	21 (12%)	13 (8.8%)	10 (4.8%)	
	4	23 (2.9%)	4 (3.3%)	3 (1.9%)	3 (1.8%)	9 (6.1%)	4 (1.9%)	
Lifetime Marijuana Use (days)		8.80 (86.3) [0-1712]	27.64 (172.7) [0-1712]	1.52 (5.5) [0-45]	1.88 (7.4) [0-60]	4.82 (29.6) [0-296]	11.83 (102.9) [0-1250]	**0.048**
Lifetime Tobacco Use (days)		3.11 (36.4) [0-900]	3.28 (27.6) [0-300]	0.22 (1.4) [0-15]	0.70 (5.4) [0-60]	1.53 (14.5) [0-175]	8.21 (66.6) [0-900]	**0.006**

Follow-Up 1		Overall[1] n = 739	Pitt[1] n = 108	SRI[1] n = 138	Duke[1] n = 149	OHSU[1] n= 139	UCSD[1] n = 205	p-value[2]
Age at Scan (years)		17.30 (2.49) [12.99-22.93]	18.20 (2.47) [13.30 -22.93]	16.44 (2.03) [12.99 -22.35]	16.93 (2.42) [13.07 -22.57]	17.47 (2.59) [13.17 -22.47]	17.55 (2.56) [13.08 -22.89]	**<0.001**
Sex								0.6
	F	381 (52%)	61 (56%)	67 (49%)	80 (54%)	73 (53%)	100 (49%)	
	M	358 (48%)	47 (44%)	71 (51%)	69 (46%)	66 (47%)	105 (51%)	

Race							
African-American/Black	87 (12%)	21 (19%)	2 (1.4%)	52 (35%)	1 (0.7%)	11 (5.4%)	
African-American & Caucasian	8 (1.1%)	1 (0.9%)	0 (0%)	3 (2.0%)	1 (0.7%)	3 (1.5%)	
Asian	59 (8.0%)	0 (0%)	19 (14%)	6 (4.0%)	11 (7.9%)	23 (11%)	
Asian Pacific Islander	1 (0.1%)	0 (0%)	0 (0%)	1 (0.7%)	0 (0%)	0 (0%)	
Asian & White	20 (2.7%)	2 (1.9%)	7 (5.1%)	1 (0.7%)	4 (2.9%)	6 (2.9%)	
Caucasian/White	555 (75%)	84 (78%)	107 (78%)	85 (57%)	119 (86%)	160 (78%)	
Native American/American Indian	2 (0.3%)	0 (0%)	1 (0.7%)	0 (0%)	1 (0.7%)	0 (0%)	
Native American & Caucasian	1 (0.1%)	0 (0%)	0 (0%)	0 (0%)	0 (0%)	1 (0.5%)	
None	2 (0.3%)	0 (0%)	2 (1.4%)	0 (0%)	0 (0%)	0 (0%)	
Pacific Islander	3 (0.4%)	0 (0%)	0 (0%)	1 (0.7%)	2 (1.4%)	0 (0%)	
Pacific Islander & Caucasian	1 (0.1%)	0 (0%)	0 (0%)	0 (0%)	0 (0%)	1 (0.5%)	
SES	16.83 (2.45) [6.00-20.00]	16.63 (2.31) [12.00 -20.00]	17.78 (1.97) [11.00 -20.00]	16.96 (2.62) [8.00 -20.00]	16.69 (2.13) [8.00 -20.00]	16.30 (2.72) [6.00 -20.00]	**<0.001**
Family AUD Density							
0	554 (75%)	84 (78%)	108 (78%)	126 (85%)	96 (69%)	140 (68%)	
0.5	108 (15%)	14 (13%)	17 (12%)	17 (11%)	28 (20%)	32 (16%)	
1	42 (5.7%)	7 (6.5%)	5 (3.6%)	4 (2.7%)	10 (7.2%)	16 (7.8%)	
1.5	19 (2.6%)	3 (2.8%)	2 (1.4%)	2 (1.3%)	2 (1.4%)	10 (4.9%)	
2	13 (1.8%)	0 (0%)	4 (2.9%)	0 (0%)	2 (1.4%)	7 (3.4%)	
2.5	1 (0.1%)	0 (0%)	0 (0%)	0 (0%)	1 (0.7%)	0 (0%)	
3	1 (0.1%)	0 (0%)	1 (0.7%)	0 (0%)	0 (0%)	0 (0%)	
4	1 (0.1%)	0 (0%)	1 (0.7%)	0 (0%)	0 (0%)	0 (0%)	
Drinking Class (Cahalan scale)							
0	512 (69%)	67 (62%)	93 (67%)	110 (74%)	103 (74%)	139 (68%)	
1	122 (17%)	15 (14%)	27 (20%)	14 (9.4%)	24 (17%)	42 (20%)	
2	19 (2.6%)	5 (4.6%)	3 (2.2%)	4 (2.7%)	2 (1.4%)	5 (2.4%)	

	3	86 (12%)	21 (19%)	15 (11%)	21 (14%)	10 (7.2%)	19 (9.3%)	
Baseline Trauma (# of events)								
	0	286 (39%)	54 (50%)	84 (61%)	25 (17%)	48 (35%)	75 (37%)	
	1	239 (32%)	31 (29%)	30 (22%)	56 (38%)	44 (32%)	78 (38%)	
	2	144 (19%)	13 (12%)	18 (13%)	48 (32%)	27 (19%)	38 (19%)	
	3	49 (6.6%)	7 (6.5%)	3 (2.2%)	18 (12%)	11 (7.9%)	10 (4.9%)	
	4	21 (2.8%)	3 (2.8%)	3 (2.2%)	2 (1.3%)	9 (6.5%)	4 (2.0%)	
Lifetime Marijuana Use (days)		16.78 (103.9) [0-1827]	37.69 (190.9) [0-1738]	12.75 (51.6) [0-380]	4.93 (14.7) [0-106]	11.90 (59.6) [0-564]	20.40 (123.3) [0-1250]	0.2
Lifetime Tobacco Use (days)		[0-1738] 12.39 (106.4)	11.97 (74.6) [0-686]	23.10 (185.5) [0-1827]	2.19 (7.4) [0-40]	5.93 (52.6) [0-615]	17.18 (113.2) [0-1320]	0.4

Follow-Up 2		Overall[1]	Pitt[1]	SRI1	Duke[1]	OHSU[1]	UCSD[1]	p-value[2]
		n = 651	n = 94	n = 114	n = 125	n= 128	n = 190	
Age at Scan (years)		18.29 (2.46) [13.98 -23.98]	19.16 (2.49) [14.32 -23.98]	17.43 (2.03) [13.98 -23.21]	17.84 (2.24) [14.05 -23.48]	18.49 (2.57) [14.23 -23.46]	18.54 (2.54) [14.09 -23.86]	**<0.001**
Sex								0.2
	F	332 (51%)	53 (56%)	50 (44%)	72 (58%)	63 (49%)	94 (49%)	
	M	319 (49%)	41 (44%)	64 (56%)	53 (42%)	65 (51%)	96 (51%)	
Race								
	African-American/Black	73 (11%)	17 (18%)	1 (0.9%)	43 (34%)	1 (0.8%)	11 (5.8%)	
	African-American & Caucasian	8 (1.2%)	1 (1.1%)	0 (0%)	3 (2.4%)	1 (0.8%)	3 (1.6%)	
	Asian	51 (7.8%)	0 (0%)	18 (16%)	3 (2.4%)	9 (7.0%)	21 (11%)	
	Asian Pacific Islander	1 (0.2%)	0 (0%)	0 (0%)	1 (0.8%)	0 (0%)	0 (0%)	
	Asian & White	19 (2.9%)	2 (2.1%)	7 (6.1%)	1 (0.8%)	4 (3.1%)	5 (2.6%)	

Caucasian/White	491 (75%)	74 (79%)	86 (75%)	73 (58%)	110 (86%)	148 (78%)	
Native American/American Indian	2 (0.3%)	0 (0%)	1 (0.9%)	0 (0%)	1 (0.8%)	0 (0%)	
Native American & Caucasian	1 (0.2%)	0 (0%)	0 (0%)	0 (0%)	0 (0%)	1 (0.5%)	
None	1 (0.2%)	0 (0%)	1 (0.9%)	0 (0%)	0 (0%)	0 (0%)	
Pacific Islander	3 (0.5%)	0 (0%)	0 (0%)	1 (0.8%)	2 (1.6%)	0 (0%)	
Pacific Islander & Caucasian	1 (0.2%)	0 (0%)	0 (0%)	0 (0%)	0 (0%)	1 (0.5%)	
SES	16.81 (2.48) [6.00 -20.00]	16.72 (2.32) [12.00 -20.00]	17.82 (1.95) [11.00 -20.00]	16.93 (2.65) [8.00 -20.00]	16.75 (2.14) [8.00 -20.00]	16.21 (2.74) [6.00 -20.00]	**<0.001**
Family AUD Density							
0	487 (75%)	74 (79%)	89 (78%)	105 (84%)	90 (70%)	129 (68%)	
0.5	96 (15%)	12 (13%)	14 (12%)	15 (12%)	26 (20%)	29 (15%)	
1	39 (6.0%)	6 (6.4%)	4 (3.5%)	4 (3.2%)	9 (7.0%)	16 (8.4%)	
1.5	17 (2.6%)	2 (2.1%)	2 (1.8%)	1 (0.8%)	2 (1.6%)	10 (5.3%)	
2	10 (1.5%)	0 (0%)	4 (3.5%)	0 (0%)	0 (0%)	6 (3.2%)	
2.5	1 (0.2%)	0 (0%)	0 (0%)	0 (0%)	1 (0.8%)	0 (0%)	
3	1 (0.2%)	0 (0%)	1 (0.9%)	0 (0%)	0 (0%)	0 (0%)	
4	0 (0%)	0 (0%)	0 (0%)	0 (0%)	0 (0%)	0 (0%)	
Drinking Class (Cahalan scale)							
0	385 (59%)	48 (51%)	61 (54%)	86 (69%)	84 (66%)	106 (56%)	
1	131 (20%)	21 (22%)	27 (24%)	21 (17%)	18 (14%)	44 (23%)	
2	32 (4.9%)	7 (7.4%)	4 (3.5%)	3 (2.4%)	9 (7.0%)	9 (4.7%)	
3	103 (16%)	18 (19%)	22 (19%)	15 (12%)	17 (13%)	31 (16%)	
Baseline Trauma (# of events)							
0	251 (39%)	49 (52%)	67 (59%)	20 (16%)	45 (35%)	70 (37%)	
1	218 (33%)	27 (29%)	28 (25%)	51 (41%)	40 (31%)	72 (38%)	
2	121 (19%)	10 (11%)	14 (12%)	37 (30%)	24 (19%)	36 (19%)	

3	42 (6.5%)	5 (5.3%)	2 (1.8%)	15 (12%)	10 (7.8%)	10 (5.3%)	
4	19 (2.9%)	3 (3.2%)	3 (2.6%)	2 (1.6%)	9 (7.0%)	2 (1.1%)	
Lifetime Marijuana Use (days)	32.80 (146.5) [0-2119]	65.50 (260.4) [0-2119]	25.68 (76.5) [0-655]	9.37 (27.5) [0-184]	26.46 (105.4) [0-918]	40.59 (167.4) [0-1590]	**<0.001**
Lifetime Tobacco Use (days)	19.80 (169.9) [0-3656]	56.22 (386.1) [0-3656]	4.51 (16.9) [0-120]	7.19 (39.9) [0-385]	11.33 (86.5) [0-955]	24.96 (137) [0-1348]	0.4

Follow-Up 3	Overall[1] n = 491	Pitt[1] n = 70	SRI1 n = 64	Duke[1] n = 85	OHSU[1] n= 107	UCSD[1] n = 165	p-value[2]
Age at Scan (years)	19.32 (2.49) [14.92 -25.07]	20.27 (2.44) [15.45 -25.07]	18.42 (2.15) [14.92 -24.09]	18.55 (2.27) [15.16 -24.48]	19.44 (2.56) [15.13 -24.45]	19.60 (2.50) [15.13 -24.85]	**<0.001**
Sex							0.3
F	255 (52%)	41 (59%)	27 (42%)	49 (58%)	55 (51%)	83 (50%)	
M	236 (48%)	29 (41%)	37 (58%)	36 (42%)	52 (49%)	82 (50%)	
Race							
African-American/Black	54 (11%)	14 (20%)	1 (1.6%)	30 (35%)	1 (0.9%)	8 (4.8%)	
African-American & Caucasian	4 (0.8%)	0 (0%)	0 (0%)	1 (1.2%)	0 (0%)	3 (1.8%)	
Asian	36 (7.3%)	0 (0%)	9 (14%)	3 (3.5%)	7 (6.5%)	17 (10%)	
Asian Pacific Islander	1 (0.2%)	0 (0%)	0 (0%)	1 (1.2%)	0 (0%)	0 (0%)	
Asian & White	14 (2.9%)	1 (1.4%)	3 (4.7%)	1 (1.2%)	4 (3.7%)	5 (3.0%)	
Caucasian/White	378 (77%)	55 (79%)	50 (78%)	49 (58%)	93 (87%)	131 (79%)	
Native American/American Indian	0 (0%)	0 (0%)	0 (0%)	0 (0%)	0 (0%)	0 (0%)	
Native American & Caucasian	1 (0.2%)	0 (0%)	0 (0%)	0 (0%)	0 (0%)	1 (0.6%)	
None	1 (0.2%)	0 (0%)	1 (1.6%)	0 (0%)	0 (0%)	0 (0%)	
Pacific Islander	2 (0.4%)	0 (0%)	0 (0%)	0 (0%)	2 (1.9%)	0 (0%)	
Pacific Islander & Caucasian	0 (0%)	0 (0%)	0 (0%)	0 (0%)	0 (0%)	0 (0%)	

SES		16.71 (2.45) [6.00 -20.00]	16.47 (2.30) [12.00 -20.00]	17.66 (1.91) [12.00 -20.00]	16.91 (2.81) [8.00 -20.00]	16.80 (2.03) [12.00 -20.00]	16.28 (2.64) [6.00 -20.00]	**0.004**
Family AUD Density								
	0	366 (75%)	55 (79%)	49 (77%)	70 (82%)	79 (74%)	113 (68%)	
	0.5	71 (14%)	9 (13%)	9 (14%)	12 (14%)	19 (18%)	22 (13%)	
	1	31 (6.3%)	5 (7.1%)	2 (3.1%)	2 (2.4%)	7 (6.5%)	15 (9.1%)	
	1.5	14 (2.9%)	1 (1.4%)	1 (1.6%)	1 (1.2%)	1 (0.9%)	10 (6.1%)	
	2	8 (1.6%)	0 (0%)	3 (4.7%)	0 (0%)	0 (0%)	5 (3.0%)	
	2.5	1 (0.2%)	0 (0%)	0 (0%)	0 (0%)	1 (0.9%)	0 (0%)	
	3	0 (0%)	0 (0%)	0 (0%)	0 (0%)	0 (0%)	0 (0%)	
	4	0 (0%)	0 (0%)	0 (0%)	0 (0%)	0 (0%)	0 (0%)	
Drinking Class (Cahalan scale)								0.077
	0	261 (53%)	39 (56%)	32 (50%)	56 (66%)	56 (52%)	78 (47%)	
	1	108 (22%)	13 (19%)	13 (20%)	18 (21%)	21 (20%)	43 (26%)	
	2	43 (8.8%)	4 (5.7%)	9 (14%)	2 (2.4%)	15 (14%)	13 (7.9%)	
	3	79 (16%)	14 (20%)	10 (16%)	9 (11%)	15 (14%)	31 (19%)	
Baseline Trauma (# of events)								
	0	187 (38%)	39 (56%)	36 (56%)	15 (18%)	38 (36%)	59 (36%)	
	1	165 (34%)	18 (26%)	17 (27%)	31 (36%)	35 (33%)	64 (39%)	
	2	89 (18%)	7 (10%)	7 (11%)	25 (29%)	19 (18%)	31 (19%)	
	3	34 (6.9%)	4 (5.7%)	2 (3.1%)	12 (14%)	7 (6.5%)	9 (5.5%)	
	4	16 (3.3%)	2 (2.9%)	2 (3.1%)	2 (2.4%)	8 (7.5%)	2 (1.2%)	
Lifetime Marijuana Use (days)		59.82 (210.7) [0-2309]	110.01 (355.7) [0-2309]	43.48 (144.3) [0-959]	18.72 (64.6) [0-446]	50.02 (164.2) [0-1220]	72.39 (222.5) [0-1695]	0.051
Lifetime Tobacco Use (days)		55.92 (525.6) [0-10796]	248.54 (1340) [0-10796]	4.95 (21.9) [0-151]	13.58 (67) [0-528]	15.29 (98.1) [0-985]	42.13 (204.1) [0-1828]	0.2

Supplementary Table S1. Demographic Characteristics by Site and Timepoint (Baseline, Follow-Up 1, Follow-Up 2, Follow-Up 3). Pitt = University of Pittsburgh Medical Center; SRI = SRI International; Duke = Duke University Medical Center; OHSU = Oregon Health and Science University; UCSD = University of California at San Diego; [1] Statistics presented: mean (SD) [minimum-maximum]; n (%) [2] Statistical tests performed: Kruskal-Wallis test; chi-square test of independence. Note – although SES and trauma variables are shown across timepoints, these

are fixed and time-invariant variables; differences in descriptive statistics across time reflect dropout of scans from analysis due to missing or unusable data.
