# Supplementary material for: Volumetric trajectories of hippocampal subfields and amygdala nuclei influenced by adolescent alcohol use and lifetime trauma
Source: Transl Psychiatry. 2021 Mar 2;11:154. doi: 10.1038/s41398-021-01275-0 (PMC7925562; doi:10.1038/s41398-021-01275-0)
Supplement: Supplementary file 8 — Table S2 [file 41398_2021_1275_MOESM8_ESM.pdf]

**DSM Criterion A Traumas Experienced (from SSAGA Interview)**	**N (Positive)**
1. Have you ever been in the Military? (This questions relates to involvement in warfare)	0
2. Have you ever been shot (not related to Military combat)?	0
3. Have you ever been stabbed (not related to Military combat)?	1
4. Have you ever been mugged or threatened with a weapon, or experienced a break-in or robbery? (and not related to Military combat)?	52
5. Have you ever been raped or sexually assaulted by a relative?	8
6. Have you ever been raped or sexually assaulted by someone not related to you (and not related to Military combat)?	21
7. Have you ever been in a natural disaster like a fire, flood, earthquake, tornado, mudslide or hurricane?	100
8. Have you ever learned you had been exposed to radiation, dioxin, or any other dangerous materials?	8
9. Have you ever experienced an unexpected, sudden death of a close friend or relative?	268
10. Have you ever been held captive, tortured, or kidnapped (not related to Military combat)?	0
11. Have you ever been diagnosed with a life- threatening illness?	7
12. Have you ever been in a serious accident?	58
13. Have you ever seen someone being seriously injured or killed(not related to Military combat)?	65
14. Have you ever unexpectedly discovered a dead body(not related to Military combat)?	9
15. Have you ever learned that any of these terrible things had happened to a close friend or relative when you were not there?	192
16. Have you ever had any other experiences that were terrible, frightening or horrible, or were repeatedly exposed to situations that were traumatic?	167
17. Do you have persistent and strong negative beliefs or expectations about yourself, others or the world, such as "I am bad," "No one can be trusted," or "The world is completely dangerous"?	76
18. After a very frightening or horrible experience, some people can't get it out of their minds. They may lose interest in people or activities; they may not sleep well; and they may become very jumpy and easily startled or frightened. Did (this/any of these) experience(s) have that effect on you that lasted one month or longer?	76
19. Number where DSM-IV or -5 PTSD criteria was met in full?	0

Table S2. DSM-4 & 5 Type A Traumas reported in the baseline NCANDA sample. Table S2 shows the reports of traumas endorsed by the parent and/or youth, on the Computerized Semi-Structured Assessment for the Genetics of Alcoholism (SSAGA) interview in the baseline NCANDA sample (N=831). This table was adopted from De Bellis et al. 2020. The SSAGA is a top down interview (Hesselbrock et al.,1999; Bucholz et al., 1994). The SSAGA was modified for adolescents and includes an interview assessment of DSM-IV and 5 Axis I disorders, DSM type A traumas and PTSD interview questions (Brown et al., 2015). The mean number of traumatic events was 1.15 ($\pm$1.21), ranging from 0-6. 62% of the youth sample experienced at least one DSM type A traumatic event. Experiencing a traumatic event in childhood and adolescence is common. The NCANDA sample is similar to other population samples which reported similar rates such as the National Comorbidity Survey Replication Adolescent Supplement (NCSRAS) (McLaughlin et al., 2013).

De Bellis MD., Nooner K.B., Brumback, T., Clark, D.B., Brown, S.A. Posttraumatic Stress Symptoms Predicts Transition to Future Adolescent and Young Adult Moderate to Heavy Drinking in the NCANDA Sample. Current Addiction Reports, 2020, 7: 99–107.

Hesselbrock M, Easton C, Bucholz KK, Schuckit M, Hesselbrock V. A validity study of the SSAGA--a comparison with the SCAN. Addiction. 1999;94(9):1361-70.

Bucholz KK, Cadoret R, Cloninger CR, Dinwiddie SH, Hesselbrock VM, Nurnberger JI, et al. A new, semi-structured psychiatric interview for use in genetic linkage studies. Journal of Studies on Alcohol. 1994;55:149-58.

Brown SA, Brumback T, Tomlinson K, Cummins K, Thompson WK, Nagel BJ, et al. The National Consortium on Alcohol and NeuroDevelopment in Adolescence (NCANDA): Characterizing risk and resilience for alcohol use in adolescents. Journal of Studies on Alcohol and Drugs. 2015;76:895-908

McLaughlin KA, Koenen KC, Hill ED, Petukhova M, Sampson NA, Zaslavsky AM, et al. Trauma Exposure and Posttraumatic Stress Disorder in a National Sample of Adolescents. Journal of the American Academy of Child & Adolescent Psychiatry. 2013;52(8):815-30.e14.
