# Supplementary material for: Volumetric trajectories of hippocampal subfields and amygdala nuclei influenced by adolescent alcohol use and lifetime trauma
Source: Transl Psychiatry. 2021 Mar 2;11:154. doi: 10.1038/s41398-021-01275-0 (PMC7925562; doi:10.1038/s41398-021-01275-0)
Supplement: Supplementary file 9 — Table S3 [file 41398_2021_1275_MOESM9_ESM.pdf]

Characteristic<br>Total Scans (n = 2684)	Mean Volume<br>(mm^3)	Volume Range<br>(mm3)	% Volume of Whole<br>Hippocampus/Amygdala	# Scans<br>Omitted
L_tail	561	(512, 615)	6.42	18
L_sub_body	232	(210, 256)	2.65	21
L_CA1body	115	(102, 130)	1.32	14
L_sub_head	192	(172, 217)	2.20	23
L_fissure	151	(133, 171)	1.73	21
L_presub_head	147	(135, 162)	1.68	20
L_CA1head	536	(487, 584)	6.13	9
L_presub_body	176	(156, 197)	2.01	31
L_parasubiculum	68	(61, 76)	0.78	20
L_MOLlayerHPhead	342	(313, 374)	3.91	3
L_MOLlayerHPbody	215	(199, 233)	2.46	20
L_GCMLDGhead	153	(140, 169)	1.75	6
L_CA3body	87	(75, 101)	1.00	31
L_GCMLDGbody	123	(114, 133)	1.41	13
L_CA4head	126	(115, 139)	1.44	7
L_CA4body	113	(104, 123)	1.29	19
L_fimbria	68	(59, 80)	0.78	22
L_CA3head	121	(110, 135)	1.38	25
L_HATA	61	(55, 68)	0.70	12
L_Whole_body	1139	(1060, 1225)	13.03	6
L_Whole_head	1756	(1618, 1907)	20.08	3
L_Whole_hippo	3471	(3220, 3734)	39.70	0
R_tail	576	(524, 628)	6.59	4
R_sub_body	217	(199, 239)	2.48	2
R_CA1body	121	(109, 138)	1.38	14
R_sub_head	187	(167, 207)	2.14	6
R_fissure	151	(134, 171)	1.73	13
R_presub_head	142	(131, 154)	1.62	7
R_CA1head	554	(507, 606)	6.34	4
R_presub_body	155	(139, 175)	1.77	20
R_parasubiculum	67	(60, 74)	0.77	11
R_MOLlayerHPhead	350	(323, 381)	4.00	5
R_MOLlayerHPbody	218	(201, 237)	2.49	9
R_GCMLDGhead	162	(147, 177)	1.85	11
R_CA3body	99	(87, 113)	1.13	22
R_GCMLDGbody	124	(115, 133)	1.42	5
R_CA4head	133	(121, 146)	1.52	9
R_CA4body	116	(106, 127)	1.33	8
R_fimbria	66	(56, 76)	0.75	20
R_CA3head	130	(118, 144)	1.49	11
R_HATA	63	(56, 70)	0.72	11

R_Whole_body	1119	(1051, 1212)	12.80	0
R_Whole_head	1794	(1651, 1943)	20.52	2
R_Whole_hippo	3510	(3259, 3755)	40.15	0
L_lateral	662	(610, 724)	19.01	5
L_basal	447	(413, 490)	12.84	7
L_acc_basal	275	(251, 302)	7.90	6
L_AAA	54	(49, 60)	1.55	21
L_central	38	(34, 45)	1.09	28
L_medial	18.5	(15.5, 22.7)	0.53	39
L_cortical	24.2	(21.5, 27.4)	0.70	14
L_corticoamyg_trans	176	(161, 190)	5.05	5
L_paralaminar	47	(44, 52)	1.35	24
L_Whole_amyg	1744	(1615, 1905)	50.09	0
R_lateral	678	(629, 736)	19.47	1
R_basal	468	(432, 503)	13.44	4
R_acc_basal	295	(268, 321)	8.47	5
R_AAA	59	(53, 66)	1.69	12
R_central	41	(36, 47)	1.18	21
R_medial	20.1	(16.7, 24.4)	0.58	29
R_cortical	26	(23.2, 29.1)	0.75	6
R_corticoamyg_trans	181	(166, 196)	5.20	0
R_paralaminar	46.7	(43.4, 50.7)	1.34	0
R_Whole_amyg	1817	(1683, 1962)	52.18	0
wholeHippo	8743	(8208, 9376)		0
wholeAmyg	3482	(3205, 3818)		0
ICV	1500710	(1397730, 1633910)		0

Supplementary Table S2. Descriptive statistics for hippocampal subfield and amygdala nuclei volumes.
