# Supplementary material for: Volumetric trajectories of hippocampal subfields and amygdala nuclei influenced by adolescent alcohol use and lifetime trauma
Source: Transl Psychiatry. 2021 Mar 2;11:154. doi: 10.1038/s41398-021-01275-0 (PMC7925562; doi:10.1038/s41398-021-01275-0)
Supplement: Supplementary file 10 — Table S4 [file 41398_2021_1275_MOESM10_ESM.pdf]

Characteristic	Whole Hippocampus															
	Model 1				Model 2				Model 1 (controlling for Drug Use)				Model 2 (controlling for Drug Use)			
	Beta	95% CI[1]	p-value	q-value[2]	Beta	95% CI[1]	p-value	q-value[2]	Beta	95% CI[1]	p-value	q-value[2]	Beta	95% CI[1]	p-value	q-value[2]
age_d	-8.6	-14, -3.0	0.002	**0.009**	-6.5	-12, -0.64	0.03	0.069	-7.5	-13, -1.8	0.01	**0.029**	-5.2	-11, 0.71	0.085	0.2
age_m	-12	-35, 11	0.3	0.5	-10	-33, 13	0.4	0.5	-11	-34, 12	0.3	0.5	-9.7	-33, 13	0.4	0.5
baseline trauma	-15	-57, 26	0.5	0.6	-21	-63, 20	0.3	0.5	-15	-56, 27	0.5	0.6	-21	-63, 21	0.3	0.5
DrkClass	-12	-20, -4.2	0.003	**0.009**	-22	-33, -11	<0.001	<0.001	-12	-19, -3.8	0.004	**0.018**	-22	-33, -11	<0.001	**<0.001**
ICV	0	0.00, 0.00	<0.001	**<0.001**	0	0.00, 0.00	<0.001	**<0.001**	0	0.00, 0.00	<0.001	**<0.001**	0	0.00, 0.00	<0.001	**<0.001**
sex																
F	—	—			—	—			—	—			—	—		
M	244	146, 342	<0.001	**<0.001**	244	146, 342	<0.001	**<0.001**	247	148, 345	<0.001	**<0.001**	246	148, 344	<0.001	**<0.001**
ses	24	7.1, 41	0.006	**0.014**	24	6.7, 41	0.006	**0.022**	24	6.8, 41	0.006	**0.023**	23	6.4, 40	0.007	**0.028**
family alcohol density	25	-66, 116	0.6	0.6	25	-66, 116	0.6	0.7	26	-66, 117	0.6	0.6	26	-65, 117	0.6	0.7
race	38	-8.3, 85	0.11	0.2	38	-8.5, 85	0.11	0.2	39	-7.9, 86	0.1	0.2	39	-8.0, 85	0.1	0.2
age_d * age_m	-0.12	-2.2, 2.0	>0.9	>0.9	0.19	-1.9, 2.3	0.9	0.9	0.08	-2.0, 2.2	>0.9	>0.9	0.4	-1.7, 2.5	0.7	0.8
age_d * baseline trauma	-2	-5.6, 1.7	0.3	0.5	-4	-7.9, 0.01	0.05	0.1	-2	-5.6, 1.7	0.3	0.5	-4.1	-8.0, -0.09	0.045	0.1
age_m * baseline trauma	7.5	-8.0, 23	0.3	0.5	5.9	-9.6, 21	0.5	0.6	7.7	-7.8, 23	0.3	0.5	6.1	-9.4, 22	0.4	0.5
age_d * age_m * baseline trauma	0.44	-0.99, 1.9	0.5	0.6	0.12	-1.3, 1.6	0.9	0.9	0.5	-0.92, 1.9	0.5	0.6	0.18	-1.3, 1.6	0.8	0.8
baseline trauma * DrkClass					10	2.2, 18	0.012	**0.032**					10	2.6, 18	0.009	**0.028**
LifeTob									0.01	-0.01, 0.04	0.2	0.4	0.01	-0.01, 0.04	0.2	0.3
LifeMJ									-0.1	-0.18, -0.02	0.02	0.051	-0.1	-0.19, -0.02	0.015	**0.04**
Characteristic	Whole Amygdala															
	Model 1				Model 2				Model 1 (controlling for Drug Use)				Model 2 (controlling for Drug Use)			
	Beta	95% CI[1]	p-value	q-value[2]	Beta	95% CI[1]	p-value	q-value[2]	Beta	95% CI[1]	p-value	q-value[2]	Beta	95% CI[1]	p-value	q-value[2]
age_d	7.5	4.2, 11	<0.001	**<0.001**	7.9	4.4, 11	<0.001	**<0.001**	7.9	4.5, 11	<0.001	**<0.001**	8.3	4.8, 12	<0.001	<0.001
age_m	-14	-25, -2.9	0.014	**0.036**	-14	-25, -2.6	0.016	**0.045**	-14	-25, -2.7	0.015	**0.046**	-14	-25, -2.3	0.018	0.057
baseline trauma	-8.3	-29, 12	0.4	0.5	-9.5	-30, 11	0.4	0.5	-8.2	-28, 12	0.4	0.5	-9.4	-30, 11	0.4	0.4
DrkClass	-1.3	-5.9, 3.4	0.6	0.6	-3.2	-9.8, 3.4	0.3	0.5	-1.3	-5.9, 3.4	0.6	0.6	-3.3	-9.8, 3.3	0.3	0.4
ICV	0	0.00, 0.00	<0.001	**<0.001**	0	0.00, 0.00	<0.001	**<0.001**	0	0.00, 0.00	<0.001	**<0.001**	0	0.00, 0.00	<0.001	<0.001
sex																
F	—	—			—	—			—	—			—	—		
M	229	182, 277	<0.001	**<0.001**	229	182, 277	<0.001	**<0.001**	230	183, 278	<0.001	**<0.001**	230	182, 278	<0.001	<0.001
ses	0.21	-8.1, 8.5	>0.9	>0.9	0.15	-8.1, 8.4	>0.9	>0.9	0.16	-8.1, 8.5	>0.9	>0.9	0.08	-8.2, 8.4	>0.9	>0.9
family alcohol density	41	-2.8, 85	0.067	0.15	41	-2.9, 85	0.067	0.2	41	-2.9, 85	0.068	0.15	41	-2.9, 85	0.068	0.2

race	16	-6.3, 38	0.2	0.3	16	-6.3, 38	0.2	0.3	16	-6.1, 39	0.2	0.2	16	-6.1, 39	0.2	0.3
age_d * age_m	-0.92	-2.2, 0.31	0.14	0.3	-0.87	-2.1, 0.38	0.2	0.3	-0.84	-2.1, 0.40	0.2	0.2	-0.78	-2.0, 0.47	0.2	0.3
age_d * baseline trauma	-3.7	-5.9, -1.5	0.001	**0.003**	-4.1	-6.4, -1.7	<0.001	**0.003**	-3.7	-5.9, -1.5	<0.001	**0.003**	-4.1	-6.5, -1.7	<0.001	**0.003**
age_m * baseline trauma	5	-2.4, 12	0.2	0.3	4.7	-2.8, 12	0.2	0.3	5.1	-2.3, 13	0.2	0.2	4.8	-2.7, 12	0.2	0.3
age_d * age_m * baseline trauma	0.39	-0.47, 1.2	0.4	0.5	0.33	-0.54, 1.2	0.5	0.5	0.41	-0.45, 1.3	0.4	0.4	0.34	-0.52, 1.2	0.4	0.5
baseline trauma * DrkClass					2	-2.7, 6.6	0.4	0.5					2.1	-2.6, 6.7	0.4	0.4
LifeTob									0.01	0.00, 0.03	0.064	0.15	0.01	0.00, 0.03	0.064	0.2
LifeMJ									-0.04	-0.09, 0.01	0.13	0.2	-0.04	-0.09, 0.01	0.12	0.2

Supplementary Table 3 . Effect sizes for model predictors on whole hippocampus and amygdala volumes. Generalized additive mixed models (GAMM) output from gamm4 package in R. [1] CI = Confidence Interval; [2] False discovery rate correction for multiple testing
