# Supplementary material for: Volumetric trajectories of hippocampal subfields and amygdala nuclei influenced by adolescent alcohol use and lifetime trauma
Source: Transl Psychiatry. 2021 Mar 2;11:154. doi: 10.1038/s41398-021-01275-0 (PMC7925562; doi:10.1038/s41398-021-01275-0)
Supplement: Supplementary file 11 — Table S5 [file 41398_2021_1275_MOESM11_ESM.pdf]

Characteristic	Left Hippocampal Tail															
	Model 1				Model 2				Model 1 (controlling for Drug Use)				Model 2 (controlling for Drug Use)			
	Beta	95% CI[1]	p-value	q-value[2]	Beta	95% CI[1]	p-value	q-value[2]	Beta	95% CI[1]	p-value	q-value[2]	Beta	95% CI[1]	p-value	q-value[2]
age_d	1.1	0.37, 1.8	0.003	**0.012**	1.1	0.40, 1.9	0.002	**0.011**	1.1	0.34, 1.8	0.004	**0.019**	1.1	0.38, 1.9	0.003	**0.017**
age_m	2.2	-0.26, 4.7	0.079	0.2	2.3	-0.21, 4.7	0.074	0.2	2.2	-0.26, 4.7	0.08	0.2	2.3	-0.22, 4.7	0.075	0.2
baseline trauma	-1.8	-6.3, 2.8	0.4	0.8	-1.9	-6.5, 2.6	0.4	0.7	-1.8	-6.3, 2.8	0.4	0.9	-1.9	-6.5, 2.6	0.4	0.8
DrkClass	-1.2	-2.2, -0.24	0.015	**0.048**	-1.5	-2.9, -0.15	0.03	0.084	-1.2	-2.2, -0.23	0.015	0.057	-1.5	-2.9, -0.14	0.031	0.1
wholeHippo	0.02	0.02, 0.03	<0.001	**<0.001**	0.02	0.02, 0.03	<0.001	**<0.001**	0.02	0.02, 0.03	<0.001	**<0.001**	0.02	0.02, 0.03	<0.001	**<0.001**
sex																
F	—	—			—	—			—	—			—	—		
M	28	18, 38	<0.001	**<0.001**	28	18, 38	<0.001	**<0.001**	28	18, 37	<0.001	**<0.001**	28	18, 38	<0.001	**<0.001**
ses	2.1	0.23, 3.9	0.028	0.072	2.1	0.22, 3.9	0.028	0.084	2.1	0.23, 3.9	0.028	0.083	2.1	0.22, 3.9	0.028	0.1
family alcohol density	1.3	-8.6, 11	0.8	0.9	1.3	-8.6, 11	0.8	0.9	1.3	-8.6, 11	0.8	0.9	1.3	-8.6, 11	0.8	0.8
race	1.5	-3.4, 6.4	0.5	0.8	1.5	-3.4, 6.4	0.5	0.7	1.5	-3.4, 6.4	0.6	0.9	1.5	-3.4, 6.4	0.6	0.8
LifeTob									0	0.00, 0.00	0.7	0.9	0	0.00, 0.00	0.7	0.8
LifeMJ									0	-0.01, 0.01	0.8	0.9	0	-0.01, 0.01	0.8	0.8
age_d * age_m	0.08	-0.18, 0.34	0.6	0.8	0.09	-0.17, 0.35	0.5	0.7	0.07	-0.19, 0.33	0.6	0.9	0.08	-0.18, 0.35	0.5	0.8
age_d * baseline trauma	0.03	-0.43, 0.49	0.9	0.9	-0.03	-0.53, 0.47	0.9	0.9	0.03	-0.43, 0.49	0.9	0.9	-0.03	-0.53, 0.47	>0.9	>0.9
age_m * baseline trauma	-0.47	-2.1, 1.2	0.6	0.8	-0.52	-2.2, 1.2	0.5	0.7	-0.47	-2.1, 1.2	0.6	0.9	-0.52	-2.2, 1.2	0.5	0.8
age_d * age_m * baseline trauma	-0.03	-0.21, 0.15	0.8	0.9	-0.04	-0.22, 0.14	0.7	0.8	-0.03	-0.21, 0.15	0.7	0.9	-0.04	-0.22, 0.14	0.7	0.8
baseline trauma * DrkClass					0.31	-0.66, 1.3	0.5	0.7					0.3	-0.67, 1.3	0.5	0.8
Characteristic	Right Hippocampal Tail															
	Model 1				Model 2				Model 1 (controlling for Drug Use)				Model 2 (controlling for Drug Use)			
	Beta	95% CI[1]	p-value	q-value[2]	Beta	95% CI[1]	p-value	q-value[2]	Beta	95% CI[1]	p-value	q-value[2]	Beta	95% CI[1]	p-value	q-value[2]
age_d	0.75	0.04, 1.5	0.04	0.11	0.74	-0.01, 1.5	0.052	0.14	0.73	0.00, 1.5	0.049	0.15	0.72	-0.04, 1.5	0.063	0.2
age_m	2.9	0.37, 5.4	0.025	0.11	2.9	0.36, 5.4	0.026	0.12	2.9	0.36, 5.4	0.025	0.13	2.9	0.35, 5.4	0.026	0.14
baseline trauma	-4.8	-9.4, -0.21	0.041	0.11	-4.8	-9.4, -0.14	0.044	0.14	-4.8	-9.4, -0.22	0.041	0.15	-4.8	-9.4, -0.14	0.044	0.2
DrkClass	-0.79	-1.8, 0.21	0.12	0.2	-0.75	-2.2, 0.66	0.3	0.5	-0.79	-1.8, 0.21	0.12	0.3	-0.74	-2.1, 0.67	0.3	0.6
wholeHippo	0.02	0.02, 0.03	<0.001	**<0.001**	0.02	0.02, 0.03	<0.001	**<0.001**	0.02	0.02, 0.03	<0.001	**<0.001**	0.02	0.02, 0.03	<0.001	**<0.001**
sex																
F	—	—			—	—			—	—			—	—		
M	31	21, 41	<0.001	**<0.001**	31	21, 41	<0.001	**<0.001**	31	21, 41	<0.001	**<0.001**	31	21, 41	<0.001	**<0.001**
ses	1.6	-0.32, 3.4	0.1	0.2	1.6	-0.32, 3.4	0.1	0.2	1.6	-0.32, 3.4	0.1	0.3	1.6	-0.31, 3.4	0.1	0.3
family alcohol density	0.51	-9.6, 11	>0.9	>0.9	0.51	-9.6, 11	>0.9	>0.9	0.51	-9.6, 11	>0.9	>0.9	0.5	-9.6, 11	>0.9	>0.9

race	3.7	-1.4, 8.7	0.2	0.2	3.7	-1.4, 8.7	0.2	0.3	3.7	-1.4, 8.7	0.2	0.3	3.7	-1.4, 8.7	0.2	0.4
LifeTob									0	0.00, 0.00	0.7	0.8	0	0.00, 0.00	0.7	0.9
LifeMJ									0	-0.01, 0.01	0.7	0.8	0	-0.01, 0.01	0.7	0.9
age_d * age_m	0.04	-0.23, 0.30	0.8	0.8	0.04	-0.23, 0.30	0.8	>0.9	0.03	-0.23, 0.30	0.8	0.9	0.03	-0.24, 0.30	0.8	>0.9
age_d * baseline trauma	-0.12	-0.59, 0.35	0.6	0.7	-0.11	-0.62, 0.40	0.7	0.9	-0.12	-0.59, 0.35	0.6	0.8	-0.11	-0.62, 0.40	0.7	0.9
age_m * baseline trauma	-0.42	-2.1, 1.3	0.6	0.7	-0.41	-2.1, 1.3	0.6	0.9	-0.42	-2.1, 1.3	0.6	0.8	-0.42	-2.1, 1.3	0.6	0.9
age_d * age_m * baseline trauma	-0.09	-0.27, 0.09	0.3	0.5	-0.09	-0.27, 0.10	0.4	0.5	-0.09	-0.27, 0.09	0.3	0.6	-0.09	-0.27, 0.10	0.3	0.6
baseline trauma * DrkClass					-0.04	-1.0, 0.95	>0.9	>0.9					-0.05	-1.0, 0.94	>0.9	>0.9
Characteristic	Left Subiculum Body															
	Model 1				Model 2				Model 1 (controlling for Drug Use)				Model 2 (controlling for Drug Use)			
	Beta	95% CI[1]	p-value	q-value[2]	Beta	95% CI[1]	p-value	q-value[2]	Beta	95% CI[1]	p-value	q-value[2]	Beta	95% CI[1]	p-value	q-value[2]
age_d	1	0.75, 1.3	<0.001	**<0.001**	1	0.72, 1.3	<0.001	**<0.001**	1	0.71, 1.3	<0.001	**<0.001**	1	0.69, 1.3	<0.001	**<0.001**
age_m	1.4	0.28, 2.5	0.014	**0.046**	1.4	0.27, 2.5	0.015	0.053	1.4	0.27, 2.5	0.015	0.057	1.4	0.25, 2.5	0.016	0.065
baseline trauma	-0.57	-2.6, 1.4	0.6	0.8	-0.54	-2.6, 1.5	0.6	0.8	-0.58	-2.6, 1.4	0.6	0.7	-0.54	-2.6, 1.5	0.6	0.8
DrkClass	0.06	-0.36, 0.48	0.8	0.8	0.12	-0.47, 0.72	0.7	0.8	0.06	-0.36, 0.48	0.8	0.8	0.13	-0.46, 0.72	0.7	0.8
wholeHippo	0.01	0.01, 0.01	<0.001	**<0.001**	0.01	0.01, 0.01	<0.001	**<0.001**	0.01	0.01, 0.01	<0.001	**<0.001**	0.01	0.01, 0.01	<0.001	**<0.001**
sex																
F	—	—			—	—			—	—			—	—		
M	16	12, 20	<0.001	**<0.001**	16	12, 20	<0.001	**<0.001**	16	12, 20	<0.001	**<0.001**	16	12, 20	<0.001	**<0.001**
ses	0.5	-0.31, 1.3	0.2	0.4	0.5	-0.31, 1.3	0.2	0.4	0.51	-0.31, 1.3	0.2	0.3	0.51	-0.31, 1.3	0.2	0.4
family alcohol density	-0.55	-5.0, 3.9	0.8	0.8	-0.55	-5.0, 3.9	0.8	0.8	-0.55	-5.0, 3.9	0.8	0.8	-0.55	-5.0, 3.9	0.8	0.9
race	2.2	-0.02, 4.3	0.052	0.14	2.2	-0.02, 4.3	0.052	0.15	2.1	-0.03, 4.3	0.054	0.2	2.1	-0.04, 4.3	0.054	0.2
LifeTob									0	0.00, 0.00	0.2	0.3	0	0.00, 0.00	0.2	0.3
LifeMJ									0	0.00, 0.01	0.2	0.3	0	0.00, 0.01	0.2	0.3
age_d * age_m	-0.04	-0.15, 0.08	0.5	0.8	-0.04	-0.15, 0.07	0.5	0.8	-0.04	-0.15, 0.07	0.5	0.6	-0.04	-0.16, 0.07	0.4	0.7
age_d * baseline trauma	-0.04	-0.23, 0.16	0.7	0.8	-0.02	-0.24, 0.19	0.8	0.8	-0.03	-0.23, 0.16	0.7	0.8	-0.02	-0.23, 0.19	0.9	0.9
age_m * baseline trauma	-0.63	-1.4, 0.11	0.1	0.2	-0.62	-1.4, 0.13	0.1	0.2	-0.63	-1.4, 0.11	0.094	0.2	-0.62	-1.4, 0.12	0.1	0.3
age_d * age_m * baseline trauma	-0.06	-0.14, 0.02	0.13	0.2	-0.06	-0.13, 0.02	0.15	0.3	-0.06	-0.14, 0.02	0.12	0.3	-0.06	-0.14, 0.02	0.14	0.3
baseline trauma * DrkClass					-0.06	-0.48, 0.35	0.8	0.8					-0.07	-0.49, 0.35	0.7	0.8
Characteristic	Right Subiculum Body															
	Model 1				Model 2				Model 1 (controlling for Drug Use)				Model 2 (controlling for Drug Use)			
	Beta	95% CI[1]	p-value	q-value[2]	Beta	95% CI[1]	p-value	q-value[2]	Beta	95% CI[1]	p-value	q-value[2]	Beta	95% CI[1]	p-value	q-value[2]
age_d	0.85	0.55, 1.2	<0.001	**<0.001**	0.83	0.52, 1.1	<0.001	**<0.001**	0.82	0.52, 1.1	<0.001	**<0.001**	0.8	0.48, 1.1	<0.001	**<0.001**
age_m	1.2	0.27, 2.2	0.012	**0.04**	1.2	0.25, 2.2	0.013	**0.047**	1.2	0.26, 2.2	0.013	**0.049**	1.2	0.24, 2.1	0.015	0.058

baseline trauma	1.3	-0.44, 3.0	0.14	0.3	1.4	-0.40, 3.1	0.13	0.3	1.3	-0.45, 3.0	0.15	0.4	1.4	-0.40, 3.1	0.13	0.3
DrkClass	-0.02	-0.44, 0.40	>0.9	>0.9	0.07	-0.51, 0.66	0.8	0.9	-0.03	-0.45, 0.39	0.9	>0.9	0.07	-0.52, 0.66	0.8	0.9
wholeHippo	0.01	0.01, 0.01	<0.001	**<0.001**	0.01	0.01, 0.01	<0.001	**<0.001**	0.01	0.01, 0.01	<0.001	**<0.001**	0.01	0.01, 0.01	<0.001	**<0.001**
sex																
F	—	—			—	—			—	—			—	—		
M	13	8.9, 16	<0.001	**<0.001**	13	8.9, 16	<0.001	**<0.001**	13	8.8, 16	<0.001	**<0.001**	12	8.8, 16	<0.001	**<0.001**
ses	0.3	-0.41, 1.0	0.4	0.7	0.3	-0.41, 1.0	0.4	0.7	0.3	-0.40, 1.0	0.4	0.7	0.31	-0.40, 1.0	0.4	0.8
family alcohol density	-1.4	-5.2, 2.4	0.5	0.7	-1.4	-5.2, 2.4	0.5	0.7	-1.4	-5.2, 2.4	0.5	0.7	-1.4	-5.2, 2.4	0.5	0.8
race	0.21	-1.7, 2.1	0.8	>0.9	0.21	-1.7, 2.1	0.8	0.9	0.2	-1.7, 2.1	0.8	>0.9	0.2	-1.7, 2.1	0.8	0.9
LifeTob									0	0.00, 0.00	0.7	>0.9	0	0.00, 0.00	0.7	0.9
LifeMJ									0	0.00, 0.01	0.2	0.5	0	0.00, 0.01	0.2	0.5
age_d * age_m	0	-0.11, 0.11	>0.9	>0.9	0	-0.12, 0.11	>0.9	>0.9	-0.01	-0.12, 0.10	>0.9	>0.9	-0.01	-0.12, 0.10	0.9	0.9
age_d * baseline trauma	-0.06	-0.25, 0.14	0.6	0.7	-0.04	-0.25, 0.17	0.7	0.9	-0.06	-0.25, 0.14	0.6	0.8	-0.04	-0.25, 0.18	0.7	0.9
age_m * baseline trauma	-0.25	-0.89, 0.39	0.5	0.7	-0.23	-0.88, 0.41	0.5	0.7	-0.25	-0.89, 0.39	0.4	0.7	-0.24	-0.88, 0.41	0.5	0.8
age_d * age_m * baseline trauma	-0.07	-0.14, 0.01	0.079	0.2	-0.07	-0.14, 0.01	0.1	0.3	-0.07	-0.15, 0.01	0.071	0.2	-0.07	-0.14, 0.01	0.089	0.3
baseline trauma * DrkClass					-0.09	-0.50, 0.32	0.7	0.9					-0.1	-0.52, 0.31	0.6	0.9

Characteristic	Left Subiculum Head															
	Model 1				Model 2				Model 1 (controlling for Drug Use)				Model 2 (controlling for Drug Use)			
	Beta	95% CI[1]	p-value	q-value[2]	Beta	95% CI[1]	p-value	q-value[2]	Beta	95% CI[1]	p-value	q-value[2]	Beta	95% CI[1]	p-value	q-value[2]
age_d	0.28	-0.03, 0.58	0.074	0.14	0.2	-0.11, 0.52	0.2	0.3	0.27	-0.04, 0.58	0.084	0.2	0.2	-0.12, 0.52	0.2	0.4
age_m	-0.47	-1.5, 0.55	0.4	0.5	-0.52	-1.5, 0.50	0.3	0.4	-0.47	-1.5, 0.56	0.4	0.6	-0.52	-1.5, 0.50	0.3	0.5
baseline trauma	-0.52	-2.4, 1.3	0.6	0.7	-0.32	-2.2, 1.6	0.7	0.8	-0.53	-2.4, 1.3	0.6	0.7	-0.32	-2.2, 1.6	0.7	0.8
DrkClass	0.35	-0.07, 0.78	0.1	0.2	0.69	0.10, 1.3	0.023	**0.046**	0.35	-0.08, 0.77	0.11	0.2	0.69	0.09, 1.3	0.024	0.056
wholeHippo	0.01	0.01, 0.01	<0.001	**<0.001**	0.01	0.01, 0.01	<0.001	**<0.001**	0.01	0.01, 0.01	<0.001	**<0.001**	0.01	0.01, 0.01	<0.001	**<0.001**
sex																
F	—	—			—	—			—	—			—	—		
M	11	7.4, 15	<0.001	**<0.001**	11	7.4, 15	<0.001	**<0.001**	11	7.4, 15	<0.001	**<0.001**	11	7.4, 15	<0.001	**<0.001**
ses	1.1	0.35, 1.9	0.004	**0.011**	1.1	0.36, 1.9	0.004	**0.011**	1.1	0.35, 1.9	0.004	**0.013**	1.1	0.37, 1.9	0.004	**0.012**
family alcohol density	6.4	2.3, 10	0.002	**0.007**	6.4	2.4, 10	0.002	**0.007**	6.4	2.3, 10	0.002	**0.008**	6.4	2.3, 10	0.002	**0.008**
race	4.4	2.4, 6.4	<0.001	**<0.001**	4.4	2.4, 6.4	<0.001	**<0.001**	4.4	2.4, 6.4	<0.001	**<0.001**	4.4	2.4, 6.4	<0.001	**<0.001**
LifeTob									0	0.00, 0.00	0.6	0.7	0	0.00, 0.00	0.6	0.7
LifeMJ									0	0.00, 0.00	>0.9	>0.9	0	0.00, 0.00	0.9	>0.9
age_d * age_m	0.01	-0.10, 0.12	0.9	0.9	0	-0.11, 0.11	>0.9	>0.9	0.01	-0.10, 0.12	0.9	>0.9	0	-0.12, 0.11	>0.9	>0.9
age_d * baseline trauma	0.21	0.01, 0.41	0.04	0.087	0.27	0.06, 0.49	0.012	**0.029**	0.21	0.01, 0.40	0.041	0.1	0.27	0.06, 0.49	0.013	**0.034**

age_m * baseline trauma	0.28	-0.40, 0.97	0.4	0.5	0.34	-0.35, 1.0	0.3	0.4	0.28	-0.41, 0.97	0.4	0.6	0.33	-0.36, 1.0	0.3	0.5
age_d * age_m * baseline trauma	0.01	-0.07, 0.09	0.8	0.8	0.02	-0.06, 0.10	0.6	0.7	0.01	-0.07, 0.09	0.8	>0.9	0.02	-0.06, 0.10	0.6	0.7
baseline trauma * DrkClass					-0.34	-0.76, 0.08	0.11	0.2					-0.34	-0.76, 0.08	0.11	0.2

Characteristic	Right Subiculum Head															
	Model 1				Model 2				Model 1 (controlling for Drug Use)				Model 2 (controlling for Drug Use)			
	Beta	95% CI[1]	p-value	q-value[2]	Beta	95% CI[1]	p-value	q-value[2]	Beta	95% CI[1]	p-value	q-value[2]	Beta	95% CI[1]	p-value	q-value[2]
age_d	0.21	-0.05, 0.47	0.11	0.2	0.16	-0.11, 0.43	0.2	0.4	0.21	-0.06, 0.48	0.12	0.3	0.16	-0.12, 0.43	0.3	0.5
age_m	0.57	-0.39, 1.5	0.2	0.4	0.54	-0.43, 1.5	0.3	0.4	0.58	-0.39, 1.5	0.2	0.5	0.54	-0.43, 1.5	0.3	0.5
baseline trauma	-0.27	-2.0, 1.5	0.8	>0.9	-0.12	-1.9, 1.7	0.9	0.9	-0.27	-2.0, 1.5	0.8	>0.9	-0.12	-1.9, 1.7	0.9	>0.9
DrkClass	-0.05	-0.42, 0.31	0.8	>0.9	0.18	-0.33, 0.70	0.5	0.7	-0.06	-0.43, 0.31	0.7	>0.9	0.18	-0.34, 0.69	0.5	0.8
wholeHippo	0.01	0.01, 0.01	<0.001	**<0.001**	0.01	0.01, 0.01	<0.001	**<0.001**	0.01	0.01, 0.01	<0.001	**<0.001**	0.01	0.01, 0.01	<0.001	**<0.001**
sex																
F	—	—			—	—			—	—			—	—		
M	10	6.2, 14	<0.001	**<0.001**	10	6.2, 14	<0.001	**<0.001**	10	6.2, 14	<0.001	**<0.001**	10	6.2, 14	<0.001	**<0.001**
ses	1	0.29, 1.7	0.006	**0.027**	1	0.29, 1.7	0.006	**0.028**	1	0.29, 1.7	0.006	**0.031**	1	0.29, 1.7	0.006	**0.032**
family alcohol density	4.7	0.85, 8.6	0.017	0.056	4.7	0.86, 8.6	0.017	0.059	4.7	0.85, 8.6	0.017	0.064	4.7	0.85, 8.6	0.017	0.068
race	2.3	0.33, 4.2	0.022	0.057	2.2	0.33, 4.2	0.022	0.062	2.3	0.33, 4.2	0.022	0.066	2.2	0.33, 4.2	0.022	0.071
LifeTob									0	0.00, 0.00	0.6	>0.9	0	0.00, 0.00	0.6	0.8
LifeMJ									0	0.00, 0.00	>0.9	>0.9	0	0.00, 0.00	>0.9	>0.9
age_d * age_m	0.02	-0.08, 0.12	0.7	>0.9	0.01	-0.09, 0.11	0.8	0.9	0.02	-0.08, 0.12	0.7	>0.9	0.01	-0.09, 0.11	0.8	>0.9
age_d * baseline trauma	0.01	-0.17, 0.18	>0.9	>0.9	0.05	-0.13, 0.24	0.6	0.7	0	-0.17, 0.18	>0.9	>0.9	0.05	-0.13, 0.24	0.6	0.8
age_m * baseline trauma	-0.53	-1.2, 0.13	0.12	0.2	-0.49	-1.1, 0.17	0.15	0.3	-0.53	-1.2, 0.13	0.12	0.3	-0.49	-1.1, 0.17	0.15	0.4
age_d * age_m * baseline trauma	0	-0.07, 0.06	>0.9	>0.9	0.01	-0.06, 0.07	0.9	0.9	0	-0.07, 0.06	>0.9	>0.9	0.01	-0.06, 0.07	0.9	>0.9
baseline trauma * DrkClass					-0.24	-0.60, 0.13	0.2	0.4					-0.24	-0.60, 0.13	0.2	0.5

Characteristic	Left CA1 Body															
	Model 1				Model 2				Model 1 (controlling for Drug Use)				Model 2 (controlling for Drug Use)			
	Beta	95% CI[1]	p-value	q-value[2]	Beta	95% CI[1]	p-value	q-value[2]	Beta	95% CI[1]	p-value	q-value[2]	Beta	95% CI[1]	p-value	q-value[2]
age_d	0.24	0.04, 0.43	0.018	0.087	0.21	0.00, 0.41	0.047	0.2	0.22	0.02, 0.42	0.029	0.14	0.19	-0.02, 0.40	0.074	0.3
age_m	0.54	-0.13, 1.2	0.11	0.2	0.52	-0.15, 1.2	0.13	0.3	0.54	-0.13, 1.2	0.12	0.3	0.51	-0.16, 1.2	0.13	0.3
baseline trauma	-0.69	-1.9, 0.53	0.3	0.4	-0.61	-1.8, 0.62	0.3	0.5	-0.7	-1.9, 0.52	0.3	0.5	-0.61	-1.8, 0.62	0.3	0.5
DrkClass	0.1	-0.17, 0.38	0.5	0.6	0.24	-0.14, 0.63	0.2	0.4	0.09	-0.19, 0.36	0.5	0.7	0.23	-0.15, 0.62	0.2	0.5
wholeHippo	0.01	0.01, 0.01	<0.001	**<0.001**	0.01	0.01, 0.01	<0.001	**<0.001**	0.01	0.01, 0.01	<0.001	**<0.001**	0.01	0.01, 0.01	<0.001	**<0.001**
sex																
F	—	—			—	—			—	—			—	—		

M	2.1	-0.50, 4.7	0.11	0.2	2.1	-0.52, 4.7	0.12	0.3	2.1	-0.53, 4.7	0.12	0.3	2.1	-0.55, 4.7	0.12	0.3
ses	0.42	-0.08, 0.92	0.1	0.2	0.42	-0.08, 0.92	0.1	0.3	0.42	-0.08, 0.92	0.1	0.3	0.43	-0.07, 0.93	0.094	0.3
family alcohol density	2.1	-0.59, 4.8	0.13	0.2	2.1	-0.60, 4.8	0.13	0.3	2.1	-0.60, 4.8	0.13	0.3	2.1	-0.61, 4.8	0.13	0.3
race	0.24	-1.1, 1.6	0.7	0.8	0.23	-1.1, 1.6	0.7	0.7	0.23	-1.1, 1.6	0.7	0.8	0.23	-1.1, 1.6	0.7	0.7
LifeTob									0	0.00, 0.00	0.3	0.6	0	0.00, 0.00	0.3	0.5
LifeMJ									0	0.00, 0.00	0.5	0.7	0	0.00, 0.00	0.5	0.6
age_d * age_m	0.02	-0.05, 0.10	0.6	0.7	0.02	-0.06, 0.09	0.6	0.7	0.02	-0.05, 0.09	0.6	0.7	0.02	-0.06, 0.09	0.7	0.7
age_d * baseline trauma	0.02	-0.11, 0.15	0.8	0.8	0.05	-0.09, 0.19	0.5	0.6	0.02	-0.11, 0.14	0.8	0.8	0.05	-0.09, 0.19	0.5	0.6
age_m * baseline trauma	0.17	-0.28, 0.62	0.5	0.6	0.19	-0.26, 0.65	0.4	0.5	0.17	-0.28, 0.62	0.5	0.7	0.19	-0.26, 0.65	0.4	0.5
age_d * age_m * baseline trauma	-0.06	-0.11, -0.01	0.02	0.087	-0.06	-0.11, 0.00	0.035	0.2	-0.06	-0.11, -0.01	0.018	0.14	-0.06	-0.11, 0.00	0.032	0.3
baseline trauma * DrkClass					-0.14	-0.41, 0.13	0.3	0.5					-0.15	-0.42, 0.13	0.3	0.5
Characteristic	Right CA1 Body															
	Model 1				Model 2				Model 1 (controlling for Drug Use)				Model 2 (controlling for Drug Use)			
	Beta	95% CI[1]	p-value	q-value[2]	Beta	95% CI[1]	p-value	q-value[2]	Beta	95% CI[1]	p-value	q-value[2]	Beta	95% CI[1]	p-value	q-value[2]
age_d	0.39	0.20, 0.57	<0.001	**<0.001**	0.39	0.20, 0.58	<0.001	**<0.001**	0.36	0.17, 0.54	<0.001	**0.001**	0.36	0.16, 0.55	<0.001	**0.003**
age_m	0.6	-0.07, 1.3	0.081	0.2	0.6	-0.07, 1.3	0.079	0.2	0.59	-0.08, 1.3	0.086	0.2	0.59	-0.08, 1.3	0.086	0.2
baseline trauma	0.19	-1.0, 1.4	0.8	0.8	0.17	-1.1, 1.4	0.8	>0.9	0.18	-1.0, 1.4	0.8	0.9	0.17	-1.1, 1.4	0.8	>0.9
DrkClass	0.23	-0.03, 0.49	0.08	0.2	0.21	-0.15, 0.57	0.3	0.5	0.22	-0.04, 0.47	0.1	0.2	0.2	-0.16, 0.56	0.3	0.4
wholeHippo	0.01	0.01, 0.01	<0.001	**<0.001**	0.01	0.01, 0.01	<0.001	**<0.001**	0.01	0.01, 0.01	<0.001	**<0.001**	0.01	0.01, 0.01	<0.001	**<0.001**
sex																
F	—	—			—	—			—	—			—	—		
M	3.5	0.88, 6.1	0.009	**0.039**	3.5	0.88, 6.1	0.009	**0.042**	3.4	0.80, 6.0	0.011	0.053	3.4	0.80, 6.0	0.011	0.057
ses	0.49	0.00, 0.99	0.051	0.2	0.49	0.00, 0.99	0.051	0.2	0.5	0.01, 1.00	0.048	0.2	0.5	0.01, 1.00	0.048	0.2
family alcohol density	2.3	-0.41, 4.9	0.1	0.2	2.3	-0.41, 4.9	0.1	0.2	2.3	-0.42, 4.9	0.1	0.2	2.3	-0.42, 4.9	0.1	0.2
race	1	-0.32, 2.3	0.14	0.2	1	-0.32, 2.3	0.14	0.3	1	-0.33, 2.3	0.14	0.2	1	-0.33, 2.3	0.14	0.3
LifeTob									0	0.00, 0.00	>0.9	>0.9	0	0.00, 0.00	>0.9	>0.9
LifeMJ									0	0.00, 0.01	0.069	0.2	0	0.00, 0.01	0.07	0.2
age_d * age_m	-0.02	-0.09, 0.05	0.5	0.7	-0.02	-0.09, 0.05	0.6	0.8	-0.03	-0.09, 0.04	0.5	0.6	-0.03	-0.09, 0.04	0.5	0.7
age_d * baseline trauma	0	-0.12, 0.12	>0.9	>0.9	-0.01	-0.14, 0.12	>0.9	>0.9	0	-0.12, 0.12	>0.9	>0.9	-0.01	-0.14, 0.13	>0.9	>0.9
age_m * baseline trauma	-0.1	-0.56, 0.35	0.7	0.8	-0.11	-0.56, 0.35	0.6	0.8	-0.11	-0.57, 0.35	0.6	0.8	-0.11	-0.57, 0.35	0.6	0.8
age_d * age_m * baseline trauma	-0.02	-0.07, 0.02	0.3	0.5	-0.03	-0.07, 0.02	0.3	0.5	-0.03	-0.07, 0.02	0.3	0.4	-0.03	-0.07, 0.02	0.3	0.4
baseline trauma * DrkClass					0.02	-0.23, 0.28	0.9	>0.9					0.01	-0.24, 0.27	>0.9	>0.9
Characteristic	Left CA1 Head															
	Model 1				Model 2				Model 1 (controlling for Drug Use)				Model 2 (controlling for Drug Use)			

	Beta	95% CI[1]	p-value	q-value[2]	Beta	95% CI[1]	p-value	q-value[2]	Beta	95% CI[1]	p-value	q-value[2]	Beta	95% CI[1]	p-value	q-value[2]
age_d	0.57	0.02, 1.1	0.043	0.11	0.59	0.02, 1.2	0.042	0.12	0.52	-0.03, 1.1	0.065	0.2	0.55	-0.03, 1.1	0.065	0.2
age_m	-0.05	-1.9, 1.8	>0.9	>0.9	-0.03	-1.9, 1.8	>0.9	>0.9	-0.07	-1.9, 1.8	>0.9	>0.9	-0.05	-1.9, 1.8	>0.9	>0.9
baseline trauma	-1.8	-5.1, 1.6	0.3	0.4	-1.9	-5.3, 1.5	0.3	0.4	-1.8	-5.2, 1.6	0.3	0.4	-1.9	-5.3, 1.5	0.3	0.4
DrkClass	0.2	-0.57, 0.96	0.6	0.7	0.07	-1.0, 1.1	0.9	>0.9	0.19	-0.57, 0.96	0.6	0.7	0.08	-0.99, 1.2	0.9	>0.9
wholeHippo	0.03	0.03, 0.03	<0.001	**<0.001**	0.03	0.03, 0.03	<0.001	**<0.001**	0.03	0.03, 0.03	<0.001	**<0.001**	0.03	0.03, 0.03	<0.001	**<0.001**
sex																
F	—	—			—	—			—	—			—	—		
M	38	30, 45	<0.001	**<0.001**	38	30, 45	<0.001	**<0.001**	37	30, 45	<0.001	**<0.001**	37	30, 45	<0.001	**<0.001**
ses	3.2	1.8, 4.6	<0.001	**<0.001**	3.2	1.8, 4.6	<0.001	**<0.001**	3.2	1.8, 4.6	<0.001	**<0.001**	3.2	1.8, 4.6	<0.001	**<0.001**
family alcohol density	6.5	-0.82, 14	0.082	0.2	6.5	-0.82, 14	0.082	0.2	6.5	-0.81, 14	0.082	0.2	6.5	-0.81, 14	0.082	0.2
race	8.8	5.1, 12	<0.001	**<0.001**	8.8	5.1, 12	<0.001	**<0.001**	8.7	5.1, 12	<0.001	**<0.001**	8.7	5.1, 12	<0.001	**<0.001**
LifeTob									0	0.00, 0.00	0.2	0.3	0	0.00, 0.00	0.2	0.4
LifeMJ									0	0.00, 0.01	0.3	0.4	0	0.00, 0.01	0.3	0.4
age_d * age_m	-0.01	-0.21, 0.20	>0.9	>0.9	0	-0.21, 0.20	>0.9	>0.9	-0.02	-0.22, 0.19	0.9	>0.9	-0.01	-0.22, 0.19	>0.9	>0.9
age_d * baseline trauma	0.31	-0.05, 0.67	0.091	0.2	0.28	-0.11, 0.67	0.2	0.3	0.31	-0.05, 0.67	0.087	0.2	0.29	-0.10, 0.68	0.14	0.3
age_m * baseline trauma	1	-0.21, 2.3	0.1	0.2	1	-0.23, 2.3	0.11	0.2	1	-0.22, 2.3	0.11	0.2	1	-0.24, 2.2	0.12	0.3
age_d * age_m * baseline trauma	-0.09	-0.23, 0.05	0.2	0.3	-0.1	-0.24, 0.05	0.2	0.3	-0.09	-0.23, 0.05	0.2	0.3	-0.1	-0.24, 0.04	0.2	0.3
baseline trauma * DrkClass					0.13	-0.63, 0.89	0.7	>0.9					0.11	-0.65, 0.87	0.8	>0.9
Characteristic	Right CA1 Head															
	Model 1				Model 2				Model 1 (controlling for Drug Use)				Model 2 (controlling for Drug Use)			
	Beta	95% CI[1]	p-value	q-value[2]	Beta	95% CI[1]	p-value	q-value[2]	Beta	95% CI[1]	p-value	q-value[2]	Beta	95% CI[1]	p-value	q-value[2]
age_d	0.17	-0.35, 0.69	0.5	0.8	-0.06	-0.61, 0.48	0.8	0.9	0.29	-0.24, 0.82	0.3	0.5	0.06	-0.49, 0.61	0.8	>0.9
age_m	1.3	-0.67, 3.2	0.2	0.4	1.1	-0.84, 3.1	0.3	0.4	1.3	-0.63, 3.3	0.2	0.4	1.2	-0.80, 3.1	0.2	0.4
baseline trauma	-0.42	-4.0, 3.1	0.8	>0.9	0.24	-3.3, 3.8	0.9	0.9	-0.38	-4.0, 3.2	0.8	0.9	0.25	-3.3, 3.8	0.9	>0.9
DrkClass	0	-0.73, 0.73	>0.9	>0.9	1.1	0.06, 2.1	0.038	0.088	0.05	-0.68, 0.79	0.9	0.9	1.1	0.07, 2.1	0.036	0.082
wholeHippo	0.03	0.03, 0.03	<0.001	**<0.001**	0.03	0.03, 0.03	<0.001	**<0.001**	0.03	0.03, 0.03	<0.001	**<0.001**	0.03	0.03, 0.03	<0.001	**<0.001**
sex																
F	—	—			—	—			—	—			—	—		
M	38	31, 46	<0.001	**<0.001**	38	31, 46	<0.001	**<0.001**	39	31, 46	<0.001	**<0.001**	39	31, 46	<0.001	**<0.001**
ses	1.7	0.22, 3.1	0.024	0.079	1.7	0.25, 3.1	0.022	0.061	1.6	0.19, 3.1	0.027	0.08	1.7	0.22, 3.1	0.024	0.065
family alcohol density	6.2	-1.6, 14	0.12	0.3	6.2	-1.5, 14	0.12	0.2	6.2	-1.5, 14	0.12	0.3	6.2	-1.5, 14	0.11	0.2
race	7.7	3.8, 12	<0.001	**<0.001**	7.7	3.8, 12	<0.001	**<0.001**	7.8	3.9, 12	<0.001	**<0.001**	7.8	3.9, 12	<0.001	**<0.001**
LifeTob									0	0.00, 0.00	0.8	0.9	0	0.00, 0.00	0.8	>0.9

LifeMJ									-0.01	-0.02, 0.00	0.012	**0.044**	-0.01	-0.02, 0.00	0.016	0.053
age_d * age_m	-0.06	-0.25, 0.13	0.5	0.8	-0.09	-0.29, 0.10	0.3	0.5	-0.04	-0.23, 0.15	0.7	0.9	-0.07	-0.27, 0.12	0.5	0.7
age_d * baseline trauma	0.04	-0.30, 0.38	0.8	>0.9	0.26	-0.11, 0.63	0.2	0.3	0.04	-0.30, 0.38	0.8	0.9	0.25	-0.12, 0.62	0.2	0.3
age_m * baseline trauma	0.21	-1.1, 1.5	0.8	>0.9	0.38	-0.94, 1.7	0.6	0.7	0.23	-1.1, 1.5	0.7	0.9	0.4	-0.92, 1.7	0.6	0.7
age_d * age_m * baseline trauma	-0.04	-0.18, 0.09	0.5	0.8	-0.01	-0.14, 0.13	0.9	0.9	-0.04	-0.17, 0.10	0.6	0.9	0	-0.14, 0.13	>0.9	>0.9
baseline trauma * DrkClass					-1.1	-1.8, -0.37	0.003	**0.011**					-1.1	-1.8, -0.33	0.004	**0.017**
Characteristic	Left CA3 Body															
	Model 1				Model 2				Model 1 (controlling for Drug Use)				Model 2 (controlling for Drug Use)			
	Beta	95% CI[1]	p-value	q-value[2]	Beta	95% CI[1]	p-value	q-value[2]	Beta	95% CI[1]	p-value	q-value[2]	Beta	95% CI[1]	p-value	q-value[2]
age_d	0.13	-0.05, 0.32	0.2	0.4	0.11	-0.08, 0.30	0.3	0.5	0.12	-0.07, 0.30	0.2	0.5	0.09	-0.10, 0.28	0.4	0.5
age_m	0.5	-0.15, 1.2	0.13	0.4	0.48	-0.17, 1.1	0.2	0.4	0.49	-0.16, 1.1	0.14	0.5	0.47	-0.18, 1.1	0.2	0.5
baseline trauma	0.5	-0.68, 1.7	0.4	0.5	0.58	-0.62, 1.8	0.3	0.5	0.5	-0.69, 1.7	0.4	0.6	0.58	-0.62, 1.8	0.3	0.5
DrkClass	0.2	-0.06, 0.46	0.13	0.4	0.33	-0.03, 0.69	0.076	0.4	0.19	-0.06, 0.45	0.14	0.5	0.33	-0.03, 0.69	0.076	0.5
wholeHippo	0	0.00, 0.01	<0.001	**<0.001**	0	0.00, 0.01	<0.001	**<0.001**	0	0.00, 0.01	<0.001	**<0.001**	0	0.00, 0.01	<0.001	**<0.001**
sex																
F	—	—			—	—			—	—			—	—		
M	0.44	-2.1, 3.0	0.7	0.9	0.43	-2.1, 3.0	0.7	0.8	0.4	-2.1, 2.9	0.8	0.9	0.39	-2.1, 2.9	0.8	0.9
ses	0.41	-0.07, 0.90	0.093	0.4	0.42	-0.07, 0.90	0.091	0.4	0.42	-0.07, 0.90	0.091	0.5	0.42	-0.06, 0.90	0.088	0.5
family alcohol density	1.5	-1.1, 4.1	0.3	0.5	1.5	-1.1, 4.1	0.3	0.5	1.5	-1.1, 4.1	0.3	0.5	1.5	-1.1, 4.1	0.3	0.5
race	-0.64	-1.9, 0.65	0.3	0.5	-0.65	-1.9, 0.65	0.3	0.5	-0.65	-2.0, 0.65	0.3	0.5	-0.66	-2.0, 0.64	0.3	0.5
LifeTob									0	0.00, 0.00	0.8	0.9	0	0.00, 0.00	0.8	>0.9
LifeMJ									0	0.00, 0.00	0.4	0.6	0	0.00, 0.00	0.3	0.5
age_d * age_m	0.01	-0.06, 0.07	0.9	0.9	0	-0.07, 0.07	>0.9	>0.9	0	-0.06, 0.07	>0.9	>0.9	0	-0.07, 0.07	>0.9	>0.9
age_d * baseline trauma	0.08	-0.04, 0.19	0.2	0.5	0.1	-0.03, 0.23	0.13	0.4	0.08	-0.04, 0.20	0.2	0.5	0.1	-0.03, 0.23	0.12	0.5
age_m * baseline trauma	0.06	-0.38, 0.50	0.8	0.9	0.08	-0.36, 0.52	0.7	0.8	0.05	-0.38, 0.49	0.8	0.9	0.08	-0.36, 0.52	0.7	0.9
age_d * age_m * baseline trauma	-0.02	-0.07, 0.02	0.3	0.5	-0.02	-0.07, 0.03	0.4	0.6	-0.02	-0.07, 0.02	0.3	0.5	-0.02	-0.07, 0.03	0.4	0.6
baseline trauma * DrkClass					-0.13	-0.38, 0.12	0.3	0.5					-0.13	-0.39, 0.12	0.3	0.5
Characteristic	Right CA3 Body															
	Model 1				Model 2				Model 1 (controlling for Drug Use)				Model 2 (controlling for Drug Use)			
	Beta	95% CI[1]	p-value	q-value[2]	Beta	95% CI[1]	p-value	q-value[2]	Beta	95% CI[1]	p-value	q-value[2]	Beta	95% CI[1]	p-value	q-value[2]
age_d	0.44	0.23, 0.64	<0.001	**<0.001**	0.39	0.17, 0.60	<0.001	**0.003**	0.41	0.20, 0.61	<0.001	**<0.001**	0.35	0.14, 0.57	0.001	**0.011**
age_m	0.47	-0.19, 1.1	0.2	0.3	0.44	-0.23, 1.1	0.2	0.4	0.46	-0.20, 1.1	0.2	0.3	0.42	-0.24, 1.1	0.2	0.4
baseline trauma	0.25	-0.96, 1.5	0.7	0.7	0.39	-0.83, 1.6	0.5	0.6	0.24	-0.97, 1.5	0.7	0.7	0.39	-0.83, 1.6	0.5	0.6
DrkClass	0.23	-0.05, 0.52	0.11	0.3	0.46	0.07, 0.86	0.022	0.078	0.22	-0.07, 0.50	0.14	0.3	0.46	0.06, 0.86	0.024	0.1

wholeHippo	0	0.00, 0.01	<0.001	**<0.001**	0	0.00, 0.01	<0.001	**<0.001**	0	0.00, 0.01	<0.001	**<0.001**	0	0.00, 0.01	<0.001	**<0.001**
sex																
F	—	—			—	—			—	—			—	—		
M	3.1	0.51, 5.7	0.019	0.084	3.1	0.49, 5.7	0.02	0.078	3	0.43, 5.6	0.023	0.11	3	0.41, 5.6	0.023	0.1
ses	0.26	-0.23, 0.76	0.3	0.4	0.27	-0.23, 0.76	0.3	0.4	0.27	-0.22, 0.76	0.3	0.4	0.28	-0.22, 0.77	0.3	0.4
family alcohol density	2.1	-0.57, 4.7	0.12	0.3	2.1	-0.57, 4.7	0.12	0.3	2.1	-0.57, 4.7	0.13	0.3	2.1	-0.57, 4.7	0.13	0.3
race	0.37	-0.94, 1.7	0.6	0.6	0.37	-0.95, 1.7	0.6	0.6	0.35	-0.96, 1.7	0.6	0.7	0.35	-0.96, 1.7	0.6	0.6
LifeTob									0	0.00, 0.00	>0.9	>0.9	0	0.00, 0.00	>0.9	>0.9
LifeMJ									0	0.00, 0.01	0.11	0.3	0	0.00, 0.01	0.093	0.2
age_d * age_m	-0.04	-0.11, 0.04	0.3	0.4	-0.05	-0.12, 0.03	0.2	0.4	-0.04	-0.12, 0.03	0.3	0.4	-0.05	-0.13, 0.03	0.2	0.4
age_d * baseline trauma	-0.1	-0.23, 0.03	0.14	0.3	-0.05	-0.20, 0.09	0.5	0.6	-0.1	-0.23, 0.03	0.14	0.3	-0.05	-0.20, 0.09	0.5	0.6
age_m * baseline trauma	0.24	-0.21, 0.69	0.3	0.4	0.28	-0.17, 0.73	0.2	0.4	0.24	-0.21, 0.68	0.3	0.4	0.27	-0.18, 0.72	0.2	0.4
age_d * age_m * baseline trauma	-0.02	-0.07, 0.03	0.5	0.5	-0.01	-0.06, 0.04	0.6	0.6	-0.02	-0.07, 0.03	0.4	0.5	-0.01	-0.07, 0.04	0.6	0.6
baseline trauma * DrkClass					-0.23	-0.51, 0.05	0.11	0.3					-0.24	-0.53, 0.04	0.089	0.2
**Characteristic**	Left CA3 Head															
	Model 1				Model 2				Model 1 (controlling for Drug Use)				Model 2 (controlling for Drug Use)			
	Beta	95% CI[1]	p-value	q-value[2]	Beta	95% CI[1]	p-value	q-value[2]	Beta	95% CI[1]	p-value	q-value[2]	Beta	95% CI[1]	p-value	q-value[2]
age_d	0.07	-0.12, 0.26	0.4	0.5	0.09	-0.11, 0.28	0.4	0.5	0.07	-0.12, 0.26	0.5	0.6	0.09	-0.11, 0.29	0.4	0.6
age_m	-0.02	-0.57, 0.53	>0.9	>0.9	-0.01	-0.56, 0.54	>0.9	>0.9	-0.02	-0.57, 0.53	>0.9	>0.9	-0.01	-0.56, 0.54	>0.9	>0.9
baseline trauma	0.87	-0.13, 1.9	0.09	0.2	0.83	-0.19, 1.9	0.11	0.3	0.87	-0.13, 1.9	0.09	0.2	0.83	-0.19, 1.9	0.11	0.3
DrkClass	0.3	0.04, 0.56	0.022	0.073	0.24	-0.13, 0.60	0.2	0.4	0.3	0.04, 0.56	0.025	0.092	0.23	-0.13, 0.60	0.2	0.4
wholeHippo	0.01	0.01, 0.01	<0.001	**<0.001**	0.01	0.01, 0.01	<0.001	**<0.001**	0.01	0.01, 0.01	<0.001	**<0.001**	0.01	0.01, 0.01	<0.001	**<0.001**
sex																
F	—	—			—	—			—	—			—	—		
M	6.3	4.1, 8.4	<0.001	**<0.001**	6.3	4.1, 8.4	<0.001	**<0.001**	6.3	4.1, 8.4	<0.001	**<0.001**	6.3	4.1, 8.4	<0.001	**<0.001**
ses	0.75	0.34, 1.2	<0.001	**0.002**	0.75	0.34, 1.2	<0.001	**0.002**	0.75	0.34, 1.2	<0.001	**0.002**	0.75	0.34, 1.2	<0.001	**0.002**
family alcohol density	0.11	-2.1, 2.3	>0.9	>0.9	0.11	-2.1, 2.3	>0.9	>0.9	0.11	-2.1, 2.3	>0.9	>0.9	0.11	-2.1, 2.3	>0.9	>0.9
race	1.1	-0.03, 2.1	0.057	0.15	1.1	-0.03, 2.1	0.057	0.2	1.1	-0.03, 2.1	0.057	0.2	1.1	-0.03, 2.1	0.057	0.2
LifeTob									0	0.00, 0.00	0.5	0.6	0	0.00, 0.00	0.5	0.7
LifeMJ									0	0.00, 0.00	0.9	>0.9	0	0.00, 0.00	0.9	>0.9
age_d * age_m	0.05	-0.02, 0.12	0.2	0.3	0.05	-0.02, 0.12	0.2	0.3	0.05	-0.02, 0.12	0.2	0.3	0.05	-0.02, 0.12	0.15	0.3
age_d * baseline trauma	0.05	-0.07, 0.17	0.4	0.5	0.03	-0.10, 0.17	0.6	0.7	0.05	-0.08, 0.17	0.5	0.6	0.03	-0.10, 0.17	0.6	0.8
age_m * baseline trauma	0.21	-0.16, 0.58	0.3	0.4	0.2	-0.17, 0.57	0.3	0.4	0.21	-0.15, 0.58	0.3	0.4	0.2	-0.17, 0.57	0.3	0.5
age_d * age_m * baseline trauma	-0.04	-0.09, 0.01	0.082	0.2	-0.05	-0.09, 0.00	0.072	0.2	-0.04	-0.09, 0.01	0.082	0.2	-0.05	-0.09, 0.00	0.072	0.2

baseline trauma * DrkClass					0.07	-0.19, 0.33	0.6	0.7					0.07	-0.19, 0.33	0.6	0.8
**Characteristic**	Right CA3 Head															
	Model 1				Model 2				Model 1 (controlling for Drug Use)				Model 2 (controlling for Drug Use)			
	Beta	95% CI[1]	p-value	q-value[2]	Beta	95% CI[1]	p-value	q-value[2]	Beta	95% CI[1]	p-value	q-value[2]	Beta	95% CI[1]	p-value	q-value[2]
age_d	0.06	-0.13, 0.24	0.5	0.6	0.02	-0.17, 0.22	0.8	0.9	0.07	-0.12, 0.26	0.5	0.6	0.04	-0.16, 0.23	0.7	0.8
age_m	0.3	-0.28, 0.88	0.3	0.5	0.28	-0.30, 0.86	0.3	0.5	0.31	-0.27, 0.89	0.3	0.6	0.28	-0.30, 0.87	0.3	0.5
baseline trauma	1.2	0.18, 2.3	0.022	0.057	1.3	0.26, 2.4	0.015	**0.041**	1.2	0.19, 2.3	0.021	0.064	1.3	0.27, 2.4	0.015	**0.047**
DrkClass	0.36	0.10, 0.62	0.006	**0.027**	0.51	0.15, 0.87	0.006	**0.027**	0.37	0.11, 0.63	0.005	**0.026**	0.51	0.15, 0.88	0.005	**0.029**
wholeHippo	0.01	0.01, 0.01	<0.001	**<0.001**	0.01	0.01, 0.01	<0.001	**<0.001**	0.01	0.01, 0.01	<0.001	**<0.001**	0.01	0.01, 0.01	<0.001	**<0.001**
sex																
F	—	—			—	—			—	—			—	—		
M	8	5.7, 10	<0.001	**<0.001**	8	5.7, 10	<0.001	**<0.001**	8	5.7, 10	<0.001	**<0.001**	8	5.7, 10	<0.001	**<0.001**
ses	0.4	-0.03, 0.84	0.069	0.13	0.41	-0.03, 0.84	0.067	0.13	0.4	-0.04, 0.83	0.072	0.2	0.4	-0.03, 0.84	0.07	0.2
family alcohol density	-0.78	-3.1, 1.5	0.5	0.6	-0.79	-3.1, 1.5	0.5	0.6	-0.78	-3.1, 1.5	0.5	0.6	-0.78	-3.1, 1.5	0.5	0.6
race	1.5	0.31, 2.6	0.013	**0.043**	1.5	0.31, 2.6	0.013	**0.041**	1.5	0.31, 2.6	0.013	**0.049**	1.5	0.31, 2.6	0.013	**0.047**
LifeTob									0	0.00, 0.00	0.8	0.8	0	0.00, 0.00	0.8	0.8
LifeMJ									0	0.00, 0.00	0.5	0.6	0	0.00, 0.00	0.5	0.6
age_d * age_m	-0.07	-0.14, 0.00	0.043	0.093	-0.07	-0.14, -0.01	0.032	0.075	-0.07	-0.14, 0.00	0.049	0.12	-0.07	-0.14, 0.00	0.037	0.1
age_d * baseline trauma	0.02	-0.10, 0.14	0.8	0.8	0.05	-0.08, 0.18	0.5	0.6	0.02	-0.10, 0.14	0.7	0.8	0.05	-0.08, 0.18	0.5	0.6
age_m * baseline trauma	0.18	-0.21, 0.58	0.4	0.5	0.21	-0.19, 0.60	0.3	0.5	0.19	-0.21, 0.58	0.4	0.6	0.21	-0.18, 0.60	0.3	0.5
age_d * age_m * baseline trauma	0	-0.05, 0.04	>0.9	>0.9	0	-0.04, 0.05	>0.9	>0.9	0	-0.05, 0.05	>0.9	>0.9	0	-0.04, 0.05	0.9	0.9
baseline trauma * DrkClass					-0.15	-0.41, 0.10	0.2	0.4					-0.15	-0.40, 0.11	0.3	0.5
**Characteristic**	Left CA4 Body															
	Model 1				Model 2				Model 1 (controlling for Drug Use)				Model 2 (controlling for Drug Use)			
	Beta	95% CI[1]	p-value	q-value[2]	Beta	95% CI[1]	p-value	q-value[2]	Beta	95% CI[1]	p-value	q-value[2]	Beta	95% CI[1]	p-value	q-value[2]
age_d	0.35	0.17, 0.53	<0.001	**0.001**	0.3	0.11, 0.49	0.002	**0.012**	0.36	0.17, 0.54	<0.001	**0.001**	0.31	0.12, 0.50	0.001	**0.012**
age_m	0.38	-0.02, 0.78	0.066	0.3	0.34	-0.06, 0.74	0.1	0.3	0.38	-0.02, 0.78	0.062	0.3	0.35	-0.05, 0.75	0.091	0.3
baseline trauma	-0.31	-1.0, 0.42	0.4	0.5	-0.18	-0.92, 0.56	0.6	0.7	-0.31	-1.0, 0.42	0.4	0.5	-0.18	-0.92, 0.56	0.6	0.7
DrkClass	0.18	-0.07, 0.43	0.15	0.4	0.39	0.04, 0.74	0.027	0.13	0.18	-0.07, 0.43	0.2	0.4	0.39	0.04, 0.74	0.028	0.2
wholeHippo	0.01	0.01, 0.01	<0.001	**<0.001**	0.01	0.01, 0.01	<0.001	**<0.001**	0.01	0.01, 0.01	<0.001	**<0.001**	0.01	0.01, 0.01	<0.001	**<0.001**
sex																
F	—	—			—	—			—	—			—	—		
M	0.76	-0.82, 2.3	0.3	0.5	0.74	-0.83, 2.3	0.4	0.5	0.78	-0.79, 2.4	0.3	0.5	0.76	-0.81, 2.3	0.3	0.5
ses	0.23	-0.07, 0.53	0.13	0.4	0.24	-0.07, 0.54	0.13	0.3	0.23	-0.07, 0.53	0.13	0.4	0.24	-0.07, 0.54	0.13	0.3

family alcohol density	1.1	-0.54, 2.7	0.2	0.4	1.1	-0.54, 2.7	0.2	0.3	1.1	-0.54, 2.7	0.2	0.4	1.1	-0.54, 2.7	0.2	0.3
race	-0.09	-0.88, 0.70	0.8	0.9	-0.09	-0.88, 0.70	0.8	0.8	-0.08	-0.87, 0.70	0.8	0.9	-0.09	-0.87, 0.70	0.8	0.8
LifeTob									0	0.00, 0.00	0.2	0.4	0	0.00, 0.00	0.2	0.3
LifeMJ									0	0.00, 0.00	0.4	0.5	0	0.00, 0.00	0.5	0.6
age_d * age_m	0.04	-0.03, 0.11	0.3	0.5	0.03	-0.03, 0.10	0.3	0.5	0.04	-0.03, 0.11	0.2	0.4	0.04	-0.03, 0.10	0.3	0.5
age_d * baseline trauma	0.06	-0.06, 0.18	0.3	0.5	0.1	-0.03, 0.23	0.12	0.3	0.06	-0.06, 0.18	0.3	0.5	0.1	-0.03, 0.23	0.13	0.3
age_m * baseline trauma	0.01	-0.26, 0.28	>0.9	>0.9	0.04	-0.23, 0.31	0.8	0.8	0.01	-0.26, 0.28	>0.9	>0.9	0.04	-0.23, 0.31	0.7	0.8
age_d * age_m * baseline trauma	-0.02	-0.07, 0.02	0.3	0.5	-0.02	-0.06, 0.03	0.5	0.6	-0.02	-0.07, 0.02	0.3	0.5	-0.02	-0.06, 0.03	0.5	0.6
baseline trauma * DrkClass					-0.21	-0.46, 0.03	0.092	0.3					-0.21	-0.46, 0.04	0.095	0.3
Characteristic	Right CA4 Body															
	Model 1				Model 2				Model 1 (controlling for Drug Use)				Model 2 (controlling for Drug Use)			
	Beta	95% CI[1]	p-value	q-value[2]	Beta	95% CI[1]	p-value	q-value[2]	Beta	95% CI[1]	p-value	q-value[2]	Beta	95% CI[1]	p-value	q-value[2]
age_d	0.5	0.30, 0.69	<0.001	**<0.001**	0.44	0.24, 0.64	<0.001	**<0.001**	0.49	0.29, 0.68	<0.001	**<0.001**	0.43	0.22, 0.63	<0.001	**<0.001**
age_m	0.49	0.04, 0.94	0.035	0.11	0.45	-0.01, 0.90	0.054	0.2	0.48	0.03, 0.94	0.036	0.13	0.44	-0.01, 0.90	0.056	0.2
baseline trauma	-0.12	-0.94, 0.70	0.8	0.8	0.04	-0.80, 0.87	>0.9	>0.9	-0.12	-0.95, 0.70	0.8	0.8	0.04	-0.80, 0.87	>0.9	>0.9
DrkClass	-0.05	-0.31, 0.22	0.7	0.8	0.21	-0.16, 0.59	0.3	0.5	-0.06	-0.33, 0.21	0.7	0.8	0.2	-0.17, 0.58	0.3	0.6
wholeHippo	0.01	0.01, 0.01	<0.001	**<0.001**	0.01	0.01, 0.01	<0.001	**<0.001**	0.01	0.01, 0.01	<0.001	**<0.001**	0.01	0.01, 0.01	<0.001	**<0.001**
sex																
F	—	—			—	—			—	—			—	—		
M	2.7	0.97, 4.5	0.003	**0.011**	2.7	0.95, 4.5	0.003	**0.013**	2.7	0.95, 4.5	0.003	**0.014**	2.7	0.93, 4.5	0.003	**0.016**
ses	0.04	-0.30, 0.38	0.8	0.8	0.05	-0.29, 0.39	0.8	>0.9	0.05	-0.29, 0.39	0.8	0.8	0.05	-0.29, 0.39	0.8	0.9
family alcohol density	0.18	-1.6, 2.0	0.8	0.8	0.18	-1.6, 2.0	0.8	>0.9	0.18	-1.6, 2.0	0.8	0.8	0.17	-1.6, 2.0	0.8	>0.9
race	0.16	-0.73, 1.1	0.7	0.8	0.16	-0.73, 1.0	0.7	>0.9	0.15	-0.74, 1.0	0.7	0.8	0.15	-0.74, 1.0	0.7	0.9
LifeTob									0	0.00, 0.00	0.4	0.8	0	0.00, 0.00	0.4	0.7
LifeMJ									0	0.00, 0.00	0.6	0.8	0	0.00, 0.00	0.5	0.8
age_d * age_m	-0.03	-0.10, 0.04	0.4	0.8	-0.04	-0.11, 0.03	0.3	0.5	-0.03	-0.10, 0.04	0.4	0.8	-0.04	-0.11, 0.03	0.3	0.6
age_d * baseline trauma	-0.03	-0.15, 0.10	0.7	0.8	0.02	-0.11, 0.16	0.7	>0.9	-0.03	-0.16, 0.10	0.7	0.8	0.02	-0.11, 0.16	0.7	0.9
age_m * baseline trauma	0.04	-0.27, 0.34	0.8	0.8	0.08	-0.23, 0.38	0.6	>0.9	0.03	-0.27, 0.34	0.8	0.8	0.08	-0.23, 0.38	0.6	0.9
age_d * age_m * baseline trauma	-0.03	-0.08, 0.02	0.2	0.5	-0.03	-0.08, 0.02	0.3	0.5	-0.03	-0.08, 0.01	0.2	0.5	-0.03	-0.08, 0.02	0.3	0.6
baseline trauma * DrkClass					-0.26	-0.52, 0.01	0.055	0.2					-0.26	-0.53, 0.00	0.05	0.2
Characteristic	Left CA4 Head															
	Model 1				Model 2				Model 1 (controlling for Drug Use)				Model 2 (controlling for Drug Use)			
	Beta	95% CI[1]	p-value	q-value[2]	Beta	95% CI[1]	p-value	q-value[2]	Beta	95% CI[1]	p-value	q-value[2]	Beta	95% CI[1]	p-value	q-value[2]
age_d	0.13	-0.06, 0.32	0.2	0.3	0.14	-0.06, 0.34	0.2	0.4	0.13	-0.06, 0.32	0.2	0.4	0.15	-0.05, 0.35	0.2	0.4

age_m	-0.18	-0.65, 0.28	0.4	0.5	-0.17	-0.64, 0.29	0.5	0.6	-0.18	-0.65, 0.28	0.4	0.5	-0.17	-0.64, 0.30	0.5	0.7
baseline trauma	0.51	-0.34, 1.4	0.2	0.3	0.46	-0.41, 1.3	0.3	0.4	0.51	-0.34, 1.4	0.2	0.4	0.46	-0.41, 1.3	0.3	0.5
DrkClass	0.14	-0.12, 0.40	0.3	0.3	0.06	-0.31, 0.43	0.7	0.7	0.14	-0.12, 0.40	0.3	0.4	0.06	-0.31, 0.43	0.7	0.8
wholeHippo	0.01	0.01, 0.01	<0.001	**<0.001**	0.01	0.01, 0.01	<0.001	**<0.001**	0.01	0.01, 0.01	<0.001	**<0.001**	0.01	0.01, 0.01	<0.001	**<0.001**
sex																
F	—	—			—	—			—	—			—	—		
M	4.7	2.9, 6.5	<0.001	**<0.001**	4.7	2.9, 6.5	<0.001	**<0.001**	4.7	2.9, 6.5	<0.001	**<0.001**	4.7	2.9, 6.6	<0.001	**<0.001**
ses	0.7	0.35, 1.0	<0.001	**<0.001**	0.7	0.35, 1.0	<0.001	**<0.001**	0.7	0.35, 1.0	<0.001	**<0.001**	0.7	0.35, 1.0	<0.001	**<0.001**
family alcohol density	0.48	-1.4, 2.3	0.6	0.6	0.48	-1.4, 2.3	0.6	0.7	0.48	-1.4, 2.3	0.6	0.7	0.48	-1.4, 2.3	0.6	0.7
race	1.2	0.33, 2.2	0.008	**0.025**	1.2	0.33, 2.2	0.008	**0.027**	1.2	0.33, 2.2	0.008	**0.028**	1.2	0.34, 2.2	0.008	**0.03**
LifeTob									0	0.00, 0.00	0.7	0.8	0	0.00, 0.00	0.7	0.8
LifeMJ									0	0.00, 0.00	0.8	0.8	0	0.00, 0.00	0.8	0.8
age_d * age_m	0.04	-0.03, 0.11	0.2	0.3	0.04	-0.03, 0.11	0.2	0.4	0.04	-0.03, 0.11	0.2	0.4	0.04	-0.03, 0.12	0.2	0.4
age_d * baseline trauma	0.09	-0.03, 0.22	0.2	0.3	0.08	-0.06, 0.21	0.3	0.4	0.09	-0.03, 0.22	0.2	0.4	0.07	-0.06, 0.21	0.3	0.5
age_m * baseline trauma	0.22	-0.10, 0.53	0.2	0.3	0.2	-0.11, 0.52	0.2	0.4	0.22	-0.09, 0.53	0.2	0.4	0.2	-0.11, 0.52	0.2	0.4
age_d * age_m * baseline trauma	-0.04	-0.09, 0.01	0.13	0.3	-0.04	-0.09, 0.01	0.11	0.3	-0.04	-0.09, 0.01	0.13	0.4	-0.04	-0.09, 0.01	0.12	0.4
baseline trauma * DrkClass					0.08	-0.18, 0.34	0.5	0.6					0.08	-0.18, 0.34	0.5	0.7

Characteristic	Right CA4 Head															
	Model 1				Model 2				Model 1 (controlling for Drug Use)				Model 2 (controlling for Drug Use)			
	Beta	95% CI[1]	p-value	q-value[2]	Beta	95% CI[1]	p-value	q-value[2]	Beta	95% CI[1]	p-value	q-value[2]	Beta	95% CI[1]	p-value	q-value[2]
age_d	0.23	0.05, 0.41	0.011	**0.047**	0.18	-0.01, 0.36	0.061	0.12	0.22	0.04, 0.40	0.015	0.069	0.17	-0.02, 0.35	0.082	0.2
age_m	-0.2	-0.67, 0.26	0.4	0.5	-0.24	-0.71, 0.22	0.3	0.4	-0.21	-0.67, 0.26	0.4	0.5	-0.25	-0.72, 0.22	0.3	0.4
baseline trauma	0.4	-0.44, 1.3	0.4	0.5	0.56	-0.30, 1.4	0.2	0.3	0.4	-0.45, 1.3	0.4	0.5	0.56	-0.30, 1.4	0.2	0.3
DrkClass	0.13	-0.12, 0.38	0.3	0.4	0.38	0.04, 0.73	0.031	0.088	0.13	-0.12, 0.38	0.3	0.5	0.38	0.04, 0.73	0.03	0.1
wholeHippo	0.01	0.01, 0.01	<0.001	**<0.001**	0.01	0.01, 0.01	<0.001	**<0.001**	0.01	0.01, 0.01	<0.001	**<0.001**	0.01	0.01, 0.01	<0.001	**<0.001**
sex																
F	—	—			—	—			—	—			—	—		
M	5.2	3.3, 7.0	<0.001	**<0.001**	5.2	3.3, 7.0	<0.001	**<0.001**	5.1	3.3, 7.0	<0.001	**<0.001**	5.1	3.3, 7.0	<0.001	**<0.001**
ses	0.38	0.03, 0.74	0.034	0.089	0.39	0.04, 0.74	0.031	0.088	0.39	0.03, 0.74	0.034	0.1	0.39	0.04, 0.75	0.031	0.1
family alcohol density	0.04	-1.8, 1.9	>0.9	>0.9	0.04	-1.8, 1.9	>0.9	>0.9	0.04	-1.8, 1.9	>0.9	>0.9	0.04	-1.8, 1.9	>0.9	>0.9
race	1.1	0.20, 2.1	0.018	0.058	1.1	0.20, 2.1	0.018	0.083	1.1	0.19, 2.1	0.018	0.069	1.1	0.19, 2.1	0.018	0.1
LifeTob									0	0.00, 0.00	0.5	0.6	0	0.00, 0.00	0.5	0.6
LifeMJ									0	0.00, 0.00	0.5	0.6	0	0.00, 0.00	0.5	0.6
age_d * age_m	-0.05	-0.11, 0.02	0.2	0.3	-0.06	-0.12, 0.01	0.1	0.2	-0.05	-0.12, 0.02	0.14	0.3	-0.06	-0.12, 0.01	0.088	0.2

age_d * baseline trauma	-0.08	-0.20, 0.03	0.2	0.3	-0.03	-0.16, 0.09	0.6	0.7	-0.08	-0.20, 0.04	0.2	0.4	-0.03	-0.16, 0.10	0.6	0.7
age_m * baseline trauma	0.18	-0.14, 0.50	0.3	0.4	0.22	-0.10, 0.54	0.2	0.3	0.18	-0.14, 0.50	0.3	0.5	0.22	-0.10, 0.54	0.2	0.3
age_d * age_m * baseline trauma	0	-0.04, 0.05	>0.9	>0.9	0.01	-0.04, 0.05	0.7	0.8	0	-0.04, 0.05	>0.9	>0.9	0.01	-0.04, 0.05	0.7	0.8
baseline trauma * DrkClass					-0.25	-0.49, -0.01	0.043	0.1					-0.25	-0.50, -0.01	0.041	0.11

Characteristic	Left Fissure															
	Model 1				Model 2				Model 1 (controlling for Drug Use)				Model 2 (controlling for Drug Use)			
	Beta	95% CI[1]	p-value	q-value[2]	Beta	95% CI[1]	p-value	q-value[2]	Beta	95% CI[1]	p-value	q-value[2]	Beta	95% CI[1]	p-value	q-value[2]
age_d	0.24	-0.39, 0.86	0.5	0.6	0.16	-0.49, 0.82	0.6	0.7	0.3	-0.33, 0.93	0.3	0.4	0.24	-0.42, 0.89	0.5	0.6
age_m	0.56	-0.31, 1.4	0.2	0.4	0.51	-0.37, 1.4	0.3	0.4	0.58	-0.29, 1.4	0.2	0.4	0.53	-0.34, 1.4	0.2	0.4
baseline trauma	0.22	-1.3, 1.8	0.8	>0.9	0.42	-1.2, 2.1	0.6	0.7	0.25	-1.3, 1.8	0.8	0.9	0.43	-1.2, 2.1	0.6	0.7
DrkClass	0.41	-0.42, 1.2	0.3	0.5	0.74	-0.42, 1.9	0.2	0.4	0.5	-0.34, 1.3	0.2	0.4	0.79	-0.38, 2.0	0.2	0.4
wholeHippo	0.01	0.01, 0.01	<0.001	**<0.001**	0.01	0.01, 0.01	<0.001	**<0.001**	0.01	0.01, 0.01	<0.001	**<0.001**	0.01	0.01, 0.01	<0.001	**<0.001**
sex																
F	—	—			—	—			—	—			—	—		
M	3.5	0.03, 7.0	0.048	0.2	3.5	0.03, 7.0	0.049	0.2	3.6	0.13, 7.1	0.042	0.2	3.6	0.13, 7.1	0.043	0.2
ses	0.47	-0.20, 1.1	0.2	0.4	0.47	-0.19, 1.1	0.2	0.4	0.44	-0.23, 1.1	0.2	0.4	0.44	-0.22, 1.1	0.2	0.4
family alcohol density	1.8	-1.7, 5.3	0.3	0.5	1.8	-1.7, 5.3	0.3	0.5	1.8	-1.6, 5.3	0.3	0.4	1.8	-1.7, 5.3	0.3	0.4
race	1.2	-0.49, 3.0	0.2	0.4	1.2	-0.49, 3.0	0.2	0.4	1.3	-0.46, 3.0	0.2	0.4	1.3	-0.46, 3.0	0.2	0.4
LifeTob									0	0.00, 0.00	0.3	0.4	0	0.00, 0.00	0.3	0.4
LifeMJ									0	-0.01, 0.00	0.2	0.4	0	-0.01, 0.00	0.2	0.4
age_d * age_m	-0.15	-0.38, 0.09	0.2	0.4	-0.16	-0.39, 0.08	0.2	0.4	-0.14	-0.37, 0.09	0.2	0.4	-0.15	-0.38, 0.09	0.2	0.4
age_d * baseline trauma	0.43	0.01, 0.84	0.043	0.2	0.49	0.05, 0.93	0.03	0.2	0.43	0.02, 0.84	0.04	0.2	0.49	0.05, 0.93	0.03	0.2
age_m * baseline trauma	-0.03	-0.62, 0.55	>0.9	>0.9	0.02	-0.58, 0.62	>0.9	>0.9	-0.02	-0.60, 0.56	>0.9	>0.9	0.02	-0.57, 0.62	>0.9	>0.9
age_d * age_m * baseline trauma	0.01	-0.15, 0.17	0.9	>0.9	0.02	-0.14, 0.18	0.8	0.9	0.02	-0.14, 0.18	0.8	>0.9	0.03	-0.14, 0.19	0.8	0.8
baseline trauma * DrkClass					-0.33	-1.1, 0.48	0.4	0.6					-0.29	-1.1, 0.52	0.5	0.6

Characteristic	Right Fissure															
	Model 1				Model 2				Model 1 (controlling for Drug Use)				Model 2 (controlling for Drug Use)			
	Beta	95% CI[1]	p-value	q-value[2]	Beta	95% CI[1]	p-value	q-value[2]	Beta	95% CI[1]	p-value	q-value[2]	Beta	95% CI[1]	p-value	q-value[2]
age_d	-0.26	-0.93, 0.42	0.5	0.7	-0.26	-0.96, 0.44	0.5	0.7	-0.27	-0.95, 0.41	0.4	0.7	-0.27	-0.98, 0.44	0.5	0.7
age_m	0.55	-0.34, 1.4	0.2	0.5	0.55	-0.35, 1.4	0.2	0.5	0.54	-0.35, 1.4	0.2	0.5	0.54	-0.36, 1.4	0.2	0.6
baseline trauma	-0.02	-1.6, 1.6	>0.9	>0.9	-0.02	-1.7, 1.7	>0.9	>0.9	-0.03	-1.6, 1.6	>0.9	>0.9	-0.02	-1.7, 1.7	>0.9	>0.9
DrkClass	1.1	0.21, 2.0	0.016	0.095	1.1	-0.15, 2.3	0.084	0.4	1.1	0.22, 2.0	0.015	0.1	1.1	-0.13, 2.4	0.079	0.4
wholeHippo	0.01	0.01, 0.01	<0.001	**<0.001**	0.01	0.01, 0.01	<0.001	**<0.001**	0.01	0.01, 0.01	<0.001	**<0.001**	0.01	0.01, 0.01	<0.001	**<0.001**
sex																

F	—	—			—	—			—	—			—	—		
M	2.3	-1.3, 5.8	0.2	0.5	2.3	-1.3, 5.8	0.2	0.5	2.2	-1.3, 5.7	0.2	0.5	2.2	-1.3, 5.7	0.2	0.6
ses	-0.03	-0.70, 0.64	>0.9	>0.9	-0.03	-0.70, 0.64	>0.9	>0.9	-0.03	-0.70, 0.64	>0.9	>0.9	-0.03	-0.70, 0.64	>0.9	>0.9
family alcohol density	1.7	-1.8, 5.2	0.3	0.6	1.7	-1.8, 5.2	0.3	0.7	1.7	-1.8, 5.2	0.3	0.6	1.7	-1.8, 5.2	0.3	0.7
race	0.17	-1.6, 1.9	0.8	>0.9	0.17	-1.6, 1.9	0.8	>0.9	0.16	-1.6, 1.9	0.9	>0.9	0.16	-1.6, 1.9	0.9	>0.9
LifeTob									0	0.00, 0.00	0.2	0.5	0	0.00, 0.00	0.2	0.6
LifeMJ									0	-0.01, 0.01	0.6	0.9	0	-0.01, 0.01	0.6	>0.9
age_d * age_m	-0.29	-0.54, -0.04	0.022	0.095	-0.29	-0.55, -0.04	0.022	0.2	-0.3	-0.55, -0.05	0.02	0.1	-0.3	-0.55, -0.05	0.02	0.2
age_d * baseline trauma	0.39	-0.05, 0.84	0.082	0.3	0.4	-0.08, 0.87	0.1	0.4	0.4	-0.04, 0.84	0.078	0.3	0.4	-0.07, 0.88	0.1	0.4
age_m * baseline trauma	-0.24	-0.82, 0.35	0.4	0.7	-0.24	-0.84, 0.37	0.4	0.7	-0.24	-0.83, 0.35	0.4	0.7	-0.24	-0.84, 0.37	0.4	0.7
age_d * age_m * baseline trauma	0.02	-0.15, 0.19	0.8	>0.9	0.02	-0.15, 0.20	0.8	>0.9	0.02	-0.15, 0.19	0.8	>0.9	0.02	-0.15, 0.20	0.8	>0.9
baseline trauma * DrkClass					-0.01	-0.87, 0.85	>0.9	>0.9					-0.01	-0.88, 0.85	>0.9	>0.9

Characteristic	Left Presubiculum Body															
	Model 1				Model 2				Model 1 (controlling for Drug Use)				Model 2 (controlling for Drug Use)			
	Beta	95% CI[1]	p-value	q-value[2]	Beta	95% CI[1]	p-value	q-value[2]	Beta	95% CI[1]	p-value	q-value[2]	Beta	95% CI[1]	p-value	q-value[2]
age_d	0.26	-0.02, 0.55	0.071	0.2	0.3	0.00, 0.59	0.053	0.15	0.25	-0.04, 0.54	0.091	0.3	0.28	-0.02, 0.59	0.068	0.2
age_m	0.88	-0.20, 2.0	0.11	0.2	0.9	-0.17, 2.0	0.1	0.2	0.88	-0.20, 2.0	0.11	0.3	0.9	-0.18, 2.0	0.1	0.3
baseline trauma	0.01	-2.0, 2.0	>0.9	>0.9	-0.08	-2.1, 1.9	>0.9	>0.9	0	-2.0, 2.0	>0.9	>0.9	-0.08	-2.1, 1.9	>0.9	>0.9
DrkClass	-0.12	-0.52, 0.28	0.6	0.8	-0.27	-0.83, 0.29	0.3	0.6	-0.14	-0.54, 0.27	0.5	0.8	-0.28	-0.84, 0.28	0.3	0.7
wholeHippo	0.01	0.00, 0.01	<0.001	**<0.001**	0.01	0.00, 0.01	<0.001	**<0.001**	0.01	0.00, 0.01	<0.001	**<0.001**	0.01	0.00, 0.01	<0.001	**<0.001**
sex																
F	—	—			—	—			—	—			—	—		
M	14	10.0, 18	<0.001	**<0.001**	14	10, 18	<0.001	**<0.001**	14	10.0, 18	<0.001	**<0.001**	14	10.0, 18	<0.001	**<0.001**
ses	0.86	0.06, 1.7	0.035	0.11	0.85	0.06, 1.6	0.036	0.12	0.86	0.07, 1.7	0.034	0.13	0.86	0.06, 1.7	0.035	0.14
family alcohol density	1.5	-2.9, 5.8	0.5	0.8	1.5	-2.9, 5.8	0.5	0.7	1.4	-2.9, 5.8	0.5	0.8	1.4	-2.9, 5.8	0.5	0.7
race	3.8	1.7, 5.9	<0.001	**0.002**	3.8	1.7, 5.9	<0.001	**0.002**	3.8	1.7, 5.9	<0.001	**0.003**	3.8	1.7, 5.9	<0.001	**0.003**
LifeTob									0	0.00, 0.00	0.4	0.8	0	0.00, 0.00	0.4	0.7
LifeMJ									0	0.00, 0.00	0.7	0.9	0	0.00, 0.00	0.8	0.8
age_d * age_m	0.02	-0.08, 0.13	0.6	0.8	0.03	-0.08, 0.14	0.6	0.7	0.02	-0.08, 0.13	0.7	0.8	0.03	-0.08, 0.13	0.6	0.7
age_d * baseline trauma	-0.02	-0.21, 0.16	0.8	0.9	-0.05	-0.26, 0.15	0.6	0.7	-0.02	-0.21, 0.16	0.8	0.9	-0.05	-0.26, 0.15	0.6	0.7
age_m * baseline trauma	-0.34	-1.1, 0.39	0.4	0.7	-0.36	-1.1, 0.37	0.3	0.6	-0.34	-1.1, 0.39	0.4	0.8	-0.36	-1.1, 0.37	0.3	0.7
age_d * age_m * baseline trauma	0.02	-0.05, 0.09	0.6	0.8	0.02	-0.06, 0.09	0.7	0.7	0.02	-0.05, 0.09	0.6	0.8	0.01	-0.06, 0.09	0.7	0.8
baseline trauma * DrkClass					0.15	-0.25, 0.54	0.5	0.7					0.14	-0.25, 0.54	0.5	0.7

Characteristic	Right Presubiculum Body

	Model 1				Model 2				Model 1 (controlling for Drug Use)				Model 2 (controlling for Drug Use)			
	Beta	95% CI[1]	p-value	q-value[2]	Beta	95% CI[1]	p-value	q-value[2]	Beta	95% CI[1]	p-value	q-value[2]	Beta	95% CI[1]	p-value	q-value[2]
age_d	-0.23	-0.52, 0.06	0.11	0.2	-0.2	-0.50, 0.10	0.2	0.4	-0.22	-0.51, 0.07	0.14	0.3	-0.19	-0.49, 0.12	0.2	0.4
age_m	0.09	-0.80, 0.97	0.8	0.8	0.11	-0.78, 1.00	0.8	0.8	0.09	-0.79, 0.98	0.8	0.8	0.12	-0.77, 1.0	0.8	0.8
baseline trauma	0.72	-0.90, 2.3	0.4	0.5	0.64	-1.00, 2.3	0.4	0.6	0.73	-0.89, 2.3	0.4	0.5	0.64	-0.99, 2.3	0.4	0.6
DrkClass	-0.36	-0.76, 0.04	0.077	0.2	-0.49	-1.1, 0.07	0.084	0.2	-0.36	-0.76, 0.04	0.075	0.2	-0.5	-1.1, 0.06	0.08	0.3
ICV	0.01	0.01, 0.01	<0.001	**<0.001**	0.01	0.01, 0.01	<0.001	**<0.001**	0.01	0.01, 0.01	<0.001	**<0.001**	0.01	0.01, 0.01	<0.001	**<0.001**
sex																
F	—	—			—	—			—	—			—	—		
M	10	6.8, 14	<0.001	**<0.001**	10	6.8, 14	<0.001	**<0.001**	10	6.8, 14	<0.001	**<0.001**	10	6.8, 14	<0.001	**<0.001**
ses	0.19	-0.48, 0.85	0.6	0.6	0.18	-0.48, 0.85	0.6	0.7	0.19	-0.48, 0.85	0.6	0.6	0.18	-0.48, 0.85	0.6	0.7
family alcohol density	-2.1	-5.6, 1.5	0.3	0.4	-2.1	-5.6, 1.5	0.3	0.4	-2.1	-5.6, 1.5	0.3	0.4	-2.1	-5.6, 1.5	0.3	0.4
race	2	0.23, 3.8	0.027	0.12	2	0.24, 3.8	0.027	0.12	2	0.24, 3.8	0.026	0.13	2	0.25, 3.8	0.026	0.14
LifeTob									0	0.00, 0.00	0.2	0.4	0	0.00, 0.00	0.2	0.4
LifeMJ									0	-0.01, 0.00	0.5	0.6	0	-0.01, 0.00	0.5	0.6
age_d * age_m	0.11	0.00, 0.21	0.046	0.15	0.11	0.01, 0.22	0.04	0.14	0.11	0.01, 0.22	0.04	0.15	0.12	0.01, 0.22	0.034	0.14
age_d * baseline trauma	0.1	-0.09, 0.29	0.3	0.4	0.07	-0.13, 0.28	0.5	0.6	0.1	-0.09, 0.29	0.3	0.5	0.07	-0.13, 0.28	0.5	0.6
age_m * baseline trauma	0.17	-0.43, 0.77	0.6	0.6	0.15	-0.46, 0.75	0.6	0.7	0.17	-0.43, 0.77	0.6	0.6	0.15	-0.46, 0.75	0.6	0.7
age_d * age_m * baseline trauma	-0.06	-0.13, 0.01	0.12	0.2	-0.06	-0.14, 0.01	0.1	0.2	-0.06	-0.13, 0.02	0.12	0.3	-0.06	-0.14, 0.01	0.1	0.3
baseline trauma * DrkClass					0.13	-0.26, 0.53	0.5	0.6					0.14	-0.26, 0.53	0.5	0.6
Characteristic	Left Presubiculum Head															
	Model 1				Model 2				Model 1 (controlling for Drug Use)				Model 2 (controlling for Drug Use)			
	Beta	95% CI[1]	p-value	q-value[2]	Beta	95% CI[1]	p-value	q-value[2]	Beta	95% CI[1]	p-value	q-value[2]	Beta	95% CI[1]	p-value	q-value[2]
age_d	0.31	0.08, 0.55	0.01	**0.026**	0.26	0.01, 0.50	0.042	0.12	0.34	0.10, 0.58	0.006	**0.017**	0.29	0.04, 0.54	0.025	0.081
age_m	0.24	-0.39, 0.88	0.5	0.7	0.2	-0.43, 0.84	0.5	0.7	0.25	-0.38, 0.89	0.4	0.7	0.21	-0.42, 0.85	0.5	0.7
baseline trauma	-1.1	-2.3, 0.04	0.059	0.13	-0.96	-2.1, 0.21	0.11	0.2	-1.1	-2.3, 0.05	0.062	0.2	-0.96	-2.1, 0.22	0.11	0.3
DrkClass	0.09	-0.24, 0.42	0.6	0.8	0.35	-0.12, 0.81	0.15	0.2	0.11	-0.22, 0.44	0.5	0.7	0.36	-0.11, 0.82	0.14	0.3
wholeHippo	0.01	0.01, 0.01	<0.001	**<0.001**	0.01	0.01, 0.01	<0.001	**<0.001**	0.01	0.01, 0.01	<0.001	**<0.001**	0.01	0.01, 0.01	<0.001	**<0.001**
sex																
F	—	—			—	—			—	—			—	—		
M	8.3	5.7, 11	<0.001	**<0.001**	8.2	5.7, 11	<0.001	**<0.001**	8.3	5.8, 11	<0.001	**<0.001**	8.3	5.8, 11	<0.001	**<0.001**
ses	0.97	0.49, 1.5	<0.001	**<0.001**	0.98	0.50, 1.5	<0.001	**<0.001**	0.97	0.48, 1.4	<0.001	**<0.001**	0.97	0.49, 1.5	<0.001	**<0.001**
family alcohol density	1.8	-0.79, 4.4	0.2	0.3	1.8	-0.78, 4.4	0.2	0.2	1.8	-0.78, 4.4	0.2	0.4	1.8	-0.77, 4.4	0.2	0.3
race	3.3	2.1, 4.6	<0.001	**<0.001**	3.3	2.1, 4.6	<0.001	**<0.001**	3.4	2.1, 4.6	<0.001	**<0.001**	3.4	2.1, 4.6	<0.001	**<0.001**

Characteristic	Model 1 Beta	Model 1 95% CI[1]	Model 1 p-value	Model 1 q-value[2]	Model 2 Beta	Model 2 95% CI[1]	Model 2 p-value	Model 2 q-value[2]	Model 1 (controlling for Drug Use) Beta	95% CI[1]	p-value	q-value[2]	Model 2 (controlling for Drug Use) Beta	95% CI[1]	p-value	q-value[2]
LifeTob									0	0.00, 0.00	0.6	0.8	0	0.00, 0.00	0.6	0.7
LifeMJ									0	-0.01, 0.00	0.2	0.4	0	-0.01, 0.00	0.2	0.3
age_d * age_m	0	-0.09, 0.08	>0.9	>0.9	-0.01	-0.10, 0.08	0.8	0.8	0	-0.09, 0.09	>0.9	>0.9	-0.01	-0.10, 0.08	0.8	>0.9
age_d * baseline trauma	0.08	-0.07, 0.24	0.3	0.5	0.13	-0.03, 0.30	0.12	0.2	0.09	-0.07, 0.24	0.3	0.5	0.13	-0.03, 0.30	0.12	0.3
age_m * baseline trauma	-0.02	-0.45, 0.41	>0.9	>0.9	0.02	-0.41, 0.46	>0.9	>0.9	-0.01	-0.44, 0.42	>0.9	>0.9	0.03	-0.41, 0.46	>0.9	>0.9
age_d * age_m * baseline trauma	0.01	-0.05, 0.07	0.8	>0.9	0.02	-0.05, 0.08	0.6	0.7	0.01	-0.05, 0.07	0.8	0.9	0.02	-0.04, 0.08	0.6	0.7
baseline trauma * DrkClass					-0.26	-0.58, 0.07	0.13	0.2					-0.25	-0.57, 0.08	0.14	0.3

Characteristic	Right Presubiculum Head															
	Model 1				Model 2				Model 1 (controlling for Drug Use)				Model 2 (controlling for Drug Use)			
	Beta	95% CI[1]	p-value	q-value[2]	Beta	95% CI[1]	p-value	q-value[2]	Beta	95% CI[1]	p-value	q-value[2]	Beta	95% CI[1]	p-value	q-value[2]
age_d	0.16	-0.05, 0.38	0.14	0.4	0.12	-0.11, 0.35	0.3	0.5	0.2	-0.02, 0.42	0.079	0.2	0.15	-0.08, 0.38	0.2	0.3
age_m	-0.04	-0.60, 0.53	0.9	0.9	-0.07	-0.64, 0.49	0.8	>0.9	-0.02	-0.59, 0.54	>0.9	>0.9	-0.05	-0.62, 0.51	0.8	>0.9
baseline trauma	-0.47	-1.5, 0.56	0.4	0.6	-0.34	-1.4, 0.71	0.5	0.7	-0.46	-1.5, 0.58	0.4	0.6	-0.33	-1.4, 0.71	0.5	0.7
DrkClass	0.09	-0.21, 0.39	0.6	0.8	0.3	-0.12, 0.72	0.2	0.3	0.09	-0.21, 0.39	0.5	0.7	0.29	-0.13, 0.71	0.2	0.3
wholeHippo	0.01	0.01, 0.01	<0.001	**<0.001**	0.01	0.01, 0.01	<0.001	**<0.001**	0.01	0.01, 0.01	<0.001	**<0.001**	0.01	0.01, 0.01	<0.001	**<0.001**
sex																
F	—	—			—	—			—	—			—	—		
M	4.9	2.7, 7.2	<0.001	**<0.001**	4.9	2.7, 7.1	<0.001	**<0.001**	5	2.8, 7.3	<0.001	**<0.001**	5	2.8, 7.2	<0.001	**<0.001**
ses	0.49	0.07, 0.92	0.023	0.076	0.5	0.07, 0.92	0.022	0.077	0.49	0.06, 0.91	0.024	0.073	0.49	0.07, 0.92	0.023	0.074
family alcohol density	1	-1.2, 3.3	0.4	0.6	1	-1.2, 3.3	0.4	0.6	1	-1.2, 3.3	0.4	0.6	1	-1.2, 3.3	0.4	0.5
race	2.5	1.4, 3.6	<0.001	**<0.001**	2.5	1.4, 3.6	<0.001	**<0.001**	2.5	1.4, 3.6	<0.001	**<0.001**	2.5	1.4, 3.6	<0.001	**<0.001**
LifeTob									0	0.00, 0.00	0.018	0.068	0	0.00, 0.00	0.018	0.073
LifeMJ									0	-0.01, 0.00	0.04	0.1	0	-0.01, 0.00	0.046	0.12
age_d * age_m	0.01	-0.07, 0.09	0.7	0.8	0.01	-0.07, 0.09	0.8	>0.9	0.02	-0.06, 0.10	0.6	0.7	0.02	-0.07, 0.10	0.7	0.9
age_d * baseline trauma	0.08	-0.07, 0.22	0.3	0.6	0.12	-0.04, 0.27	0.13	0.3	0.07	-0.07, 0.22	0.3	0.6	0.11	-0.04, 0.27	0.15	0.3
age_m * baseline trauma	-0.03	-0.41, 0.35	0.9	0.9	0	-0.38, 0.38	>0.9	>0.9	-0.02	-0.40, 0.36	>0.9	>0.9	0.01	-0.37, 0.39	>0.9	>0.9
age_d * age_m * baseline trauma	-0.01	-0.07, 0.04	0.6	0.8	-0.01	-0.06, 0.05	0.8	>0.9	-0.01	-0.07, 0.04	0.7	0.8	-0.01	-0.06, 0.05	0.8	>0.9
baseline trauma * DrkClass					-0.21	-0.51, 0.09	0.2	0.3					-0.2	-0.50, 0.10	0.2	0.3

Characteristic	Left Parasubiculum															
	Model 1				Model 2				Model 1 (controlling for Drug Use)				Model 2 (controlling for Drug Use)			
	Beta	95% CI[1]	p-value	q-value[2]	Beta	95% CI[1]	p-value	q-value[2]	Beta	95% CI[1]	p-value	q-value[2]	Beta	95% CI[1]	p-value	q-value[2]
age_d	-0.04	-0.16, 0.08	0.5	0.6	-0.05	-0.18, 0.08	0.4	0.6	-0.03	-0.16, 0.09	0.6	0.6	-0.04	-0.17, 0.09	0.5	0.6
age_m	-0.11	-0.49, 0.27	0.6	0.6	-0.11	-0.49, 0.27	0.6	0.6	-0.1	-0.48, 0.28	0.6	0.6	-0.11	-0.49, 0.27	0.6	0.6
baseline trauma	-0.3	-0.99, 0.40	0.4	0.6	-0.27	-0.97, 0.43	0.5	0.6	-0.29	-0.99, 0.40	0.4	0.6	-0.27	-0.97, 0.43	0.5	0.6

Characteristic	Beta	95% CI[1]	p-value	q-value[2]	Beta	95% CI[1]	p-value	q-value[2]	Beta	95% CI[1]	p-value	q-value[2]	Beta	95% CI[1]	p-value	q-value[2]
DrkClass	0.09	-0.08, 0.26	0.3	0.5	0.14	-0.10, 0.38	0.3	0.5	0.1	-0.07, 0.27	0.2	0.5	0.14	-0.10, 0.38	0.3	0.6
wholeHippo	0	0.00, 0.00	<0.001	**<0.001**	0	0.00, 0.00	<0.001	**<0.001**	0	0.00, 0.00	<0.001	**<0.001**	0	0.00, 0.00	<0.001	**<0.001**
sex																
F	—	—			—	—			—	—			—	—		
M	5.7	4.2, 7.1	<0.001	**<0.001**	5.6	4.2, 7.1	<0.001	**<0.001**	5.7	4.2, 7.2	<0.001	**<0.001**	5.7	4.2, 7.2	<0.001	**<0.001**
ses	0.39	0.11, 0.68	0.007	**0.022**	0.39	0.11, 0.68	0.007	**0.023**	0.39	0.11, 0.67	0.007	**0.027**	0.39	0.11, 0.67	0.007	**0.029**
family alcohol density	0.55	-0.96, 2.1	0.5	0.6	0.55	-0.96, 2.1	0.5	0.6	0.55	-0.96, 2.1	0.5	0.6	0.55	-0.96, 2.1	0.5	0.6
race	1.6	0.85, 2.3	<0.001	**<0.001**	1.6	0.85, 2.3	<0.001	**<0.001**	1.6	0.85, 2.4	<0.001	**<0.001**	1.6	0.85, 2.4	<0.001	**<0.001**
LifeTob									0	0.00, 0.00	0.6	0.6	0	0.00, 0.00	0.6	0.6
LifeMJ									0	0.00, 0.00	0.4	0.6	0	0.00, 0.00	0.4	0.6
age_d * age_m	0.05	0.01, 0.10	0.027	0.069	0.05	0.00, 0.10	0.032	0.09	0.05	0.01, 0.10	0.023	0.069	0.05	0.01, 0.10	0.027	0.088
age_d * baseline trauma	0.02	-0.06, 0.10	0.6	0.6	0.03	-0.06, 0.12	0.5	0.6	0.02	-0.06, 0.10	0.6	0.6	0.03	-0.06, 0.12	0.5	0.6
age_m * baseline trauma	0.18	-0.08, 0.43	0.2	0.4	0.19	-0.07, 0.44	0.2	0.4	0.18	-0.07, 0.44	0.2	0.4	0.19	-0.07, 0.44	0.2	0.4
age_d * age_m * baseline trauma	-0.01	-0.04, 0.02	0.4	0.6	-0.01	-0.04, 0.02	0.5	0.6	-0.01	-0.04, 0.02	0.4	0.6	-0.01	-0.04, 0.02	0.5	0.6
baseline trauma * DrkClass					-0.04	-0.21, 0.12	0.6	0.6					-0.04	-0.21, 0.13	0.6	0.6

Characteristic	Right Parasubiculum															
	Model 1				Model 2				Model 1 (controlling for Drug Use)				Model 2 (controlling for Drug Use)			
	Beta	95% CI[1]	p-value	q-value[2]	Beta	95% CI[1]	p-value	q-value[2]	Beta	95% CI[1]	p-value	q-value[2]	Beta	95% CI[1]	p-value	q-value[2]
age_d	-0.07	-0.18, 0.05	0.3	0.5	-0.09	-0.22, 0.03	0.14	0.2	-0.05	-0.17, 0.07	0.4	0.6	-0.08	-0.21, 0.05	0.2	0.3
age_m	-0.24	-0.61, 0.13	0.2	0.4	-0.26	-0.63, 0.10	0.2	0.3	-0.24	-0.60, 0.13	0.2	0.4	-0.26	-0.63, 0.11	0.2	0.3
baseline trauma	0.09	-0.59, 0.76	0.8	0.9	0.17	-0.51, 0.85	0.6	0.8	0.09	-0.58, 0.77	0.8	>0.9	0.17	-0.51, 0.85	0.6	0.8
DrkClass	0.07	-0.10, 0.24	0.4	0.7	0.2	-0.03, 0.43	0.093	0.2	0.07	-0.10, 0.24	0.4	0.6	0.2	-0.04, 0.43	0.1	0.2
wholeHippo	0	0.00, 0.00	0.005	**0.016**	0	0.00, 0.00	0.004	**0.015**	0	0.00, 0.00	0.007	**0.026**	0	0.00, 0.00	0.006	**0.022**
sex																
F	—	—			—	—			—	—			—	—		
M	4.9	3.4, 6.3	<0.001	**<0.001**	4.8	3.4, 6.3	<0.001	**<0.001**	4.9	3.5, 6.3	<0.001	**<0.001**	4.9	3.5, 6.3	<0.001	**<0.001**
ses	0.4	0.13, 0.68	0.004	**0.016**	0.4	0.13, 0.68	0.004	**0.015**	0.4	0.12, 0.67	0.005	**0.023**	0.4	0.13, 0.68	0.004	**0.022**
family alcohol density	-0.52	-2.0, 0.94	0.5	0.7	-0.52	-2.0, 0.94	0.5	0.7	-0.53	-2.0, 0.94	0.5	0.7	-0.52	-2.0, 0.94	0.5	0.6
race	1.7	0.93, 2.4	<0.001	**<0.001**	1.7	0.93, 2.4	<0.001	**<0.001**	1.7	0.94, 2.4	<0.001	**<0.001**	1.7	0.94, 2.4	<0.001	**<0.001**
LifeTob									0	0.00, 0.00	0.041	0.12	0	0.00, 0.00	0.041	0.11
LifeMJ									0	0.00, 0.00	0.12	0.2	0	0.00, 0.00	0.13	0.2
age_d * age_m	0.01	-0.04, 0.05	0.7	0.9	0	-0.04, 0.05	0.8	>0.9	0.01	-0.03, 0.06	0.6	0.8	0.01	-0.04, 0.05	0.7	0.8
age_d * baseline trauma	0.07	-0.01, 0.15	0.094	0.2	0.09	0.01, 0.18	0.032	0.088	0.07	-0.01, 0.14	0.1	0.2	0.09	0.01, 0.18	0.035	0.11
age_m * baseline trauma	0	-0.25, 0.25	>0.9	>0.9	0.02	-0.23, 0.27	0.9	>0.9	0	-0.24, 0.25	>0.9	>0.9	0.02	-0.22, 0.27	0.8	>0.9

age_d * age_m * baseline trauma	0	-0.03, 0.03	0.8	0.9	0	-0.03, 0.03	>0.9	>0.9	0	-0.03, 0.03	0.9	>0.9	0	-0.03, 0.03	>0.9	>0.9
baseline trauma * DrkClass					-0.13	-0.30, 0.03	0.12	0.2					-0.13	-0.29, 0.04	0.13	0.2

Characteristic	Left Molecular Layer HP Body															
	Model 1				Model 2				Model 1 (controlling for Drug Use)				Model 2 (controlling for Drug Use)			
	Beta	95% CI[1]	p-value	q-value[2]	Beta	95% CI[1]	p-value	q-value[2]	Beta	95% CI[1]	p-value	q-value[2]	Beta	95% CI[1]	p-value	q-value[2]
age_d	0.64	0.38, 0.89	<0.001	**<0.001**	0.61	0.35, 0.88	<0.001	**<0.001**	0.62	0.36, 0.88	<0.001	**<0.001**	0.59	0.32, 0.86	<0.001	**<0.001**
age_m	1.1	0.41, 1.7	0.002	**0.005**	1.1	0.39, 1.7	0.002	**0.007**	1.1	0.40, 1.7	0.002	**0.006**	1	0.38, 1.7	0.002	**0.009**
baseline trauma	0.35	-0.87, 1.6	0.6	0.7	0.42	-0.82, 1.6	0.5	0.6	0.34	-0.87, 1.6	0.6	0.7	0.42	-0.82, 1.6	0.5	0.6
DrkClass	0.3	-0.06, 0.65	0.1	0.2	0.41	-0.09, 0.91	0.11	0.2	0.28	-0.07, 0.64	0.12	0.3	0.4	-0.10, 0.90	0.12	0.3
wholeHippo	0.01	0.01, 0.02	<0.001	**<0.001**	0.01	0.01, 0.02	<0.001	**<0.001**	0.01	0.01, 0.02	<0.001	**<0.001**	0.01	0.01, 0.02	<0.001	**<0.001**
sex																
F	—	—			—	—			—	—			—	—		
M	4.8	2.2, 7.5	<0.001	**0.002**	4.8	2.2, 7.5	<0.001	**0.002**	4.8	2.2, 7.4	<0.001	**0.002**	4.8	2.1, 7.4	<0.001	**0.002**
ses	0.55	0.05, 1.1	0.031	0.081	0.55	0.05, 1.1	0.031	0.086	0.56	0.06, 1.1	0.029	0.088	0.56	0.06, 1.1	0.029	0.092
family alcohol density	2.6	-0.01, 5.3	0.051	0.11	2.6	-0.01, 5.3	0.052	0.12	2.6	-0.02, 5.3	0.052	0.13	2.6	-0.02, 5.3	0.052	0.14
race	0.33	-0.99, 1.7	0.6	0.7	0.33	-0.99, 1.7	0.6	0.7	0.32	-1.0, 1.7	0.6	0.7	0.32	-1.0, 1.6	0.6	0.7
LifeTob									0	0.00, 0.00	0.7	0.7	0	0.00, 0.00	0.7	0.7
LifeMJ									0	0.00, 0.01	0.5	0.6	0	0.00, 0.01	0.5	0.6
age_d * age_m	-0.01	-0.11, 0.08	0.8	0.8	-0.01	-0.11, 0.08	0.8	0.8	-0.01	-0.11, 0.08	0.8	0.8	-0.02	-0.11, 0.08	0.7	0.7
age_d * baseline trauma	0.08	-0.09, 0.25	0.4	0.5	0.1	-0.08, 0.28	0.3	0.5	0.08	-0.09, 0.25	0.4	0.6	0.1	-0.08, 0.28	0.3	0.5
age_m * baseline trauma	-0.2	-0.65, 0.25	0.4	0.5	-0.18	-0.64, 0.27	0.4	0.6	-0.21	-0.65, 0.24	0.4	0.6	-0.19	-0.64, 0.27	0.4	0.6
age_d * age_m * baseline trauma	-0.03	-0.10, 0.03	0.3	0.5	-0.03	-0.10, 0.04	0.4	0.6	-0.03	-0.10, 0.03	0.3	0.6	-0.03	-0.10, 0.03	0.4	0.6
baseline trauma * DrkClass					-0.11	-0.46, 0.24	0.5	0.6					-0.12	-0.47, 0.23	0.5	0.6

Characteristic	Right Molecular Layer HP Body															
	Model 1				Model 2				Model 1 (controlling for Drug Use)				Model 2 (controlling for Drug Use)			
	Beta	95% CI[1]	p-value	q-value[2]	Beta	95% CI[1]	p-value	q-value[2]	Beta	95% CI[1]	p-value	q-value[2]	Beta	95% CI[1]	p-value	q-value[2]
age_d	0.72	0.45, 0.98	<0.001	**<0.001**	0.68	0.41, 0.96	<0.001	**<0.001**	0.68	0.41, 0.95	<0.001	**<0.001**	0.64	0.36, 0.92	<0.001	**<0.001**
age_m	0.86	0.13, 1.6	0.022	0.07	0.84	0.10, 1.6	0.026	0.092	0.85	0.12, 1.6	0.024	0.089	0.82	0.08, 1.6	0.029	0.12
baseline trauma	0.99	-0.34, 2.3	0.15	0.3	1.1	-0.26, 2.4	0.11	0.3	0.98	-0.36, 2.3	0.2	0.3	1.1	-0.26, 2.4	0.12	0.2
DrkClass	0.17	-0.20, 0.53	0.4	0.6	0.33	-0.18, 0.85	0.2	0.4	0.14	-0.22, 0.51	0.4	0.7	0.32	-0.19, 0.84	0.2	0.4
wholeHippo	0.01	0.01, 0.01	<0.001	**<0.001**	0.01	0.01, 0.01	<0.001	**<0.001**	0.01	0.01, 0.01	<0.001	**<0.001**	0.01	0.01, 0.01	<0.001	**<0.001**
sex																
F	—	—			—	—			—	—			—	—		
M	7.7	4.8, 11	<0.001	**<0.001**	7.6	4.8, 11	<0.001	**<0.001**	7.6	4.7, 10	<0.001	**<0.001**	7.5	4.7, 10	<0.001	**<0.001**

ses	0.43	-0.12, 0.98	0.13	0.3	0.43	-0.12, 0.98	0.13	0.3	0.44	-0.11, 0.99	0.12	0.3	0.44	-0.11, 0.99	0.12	0.2
family alcohol density	0.99	-1.9, 3.9	0.5	0.6	0.99	-1.9, 3.9	0.5	0.6	0.97	-1.9, 3.9	0.5	0.7	0.97	-1.9, 3.9	0.5	0.6
race	0.59	-0.86, 2.0	0.4	0.6	0.59	-0.86, 2.0	0.4	0.6	0.57	-0.88, 2.0	0.4	0.7	0.57	-0.88, 2.0	0.4	0.6
LifeTob									0	0.00, 0.00	>0.9	>0.9	0	0.00, 0.00	>0.9	>0.9
LifeMJ									0	0.00, 0.01	0.1	0.3	0	0.00, 0.01	0.095	0.2
age_d * age_m	0	-0.10, 0.09	>0.9	>0.9	-0.01	-0.11, 0.09	0.9	>0.9	-0.01	-0.11, 0.09	0.9	>0.9	-0.01	-0.11, 0.08	0.8	0.9
age_d * baseline trauma	-0.03	-0.20, 0.15	0.8	0.8	0.01	-0.18, 0.19	>0.9	>0.9	-0.03	-0.20, 0.15	0.8	0.9	0.01	-0.18, 0.20	>0.9	>0.9
age_m * baseline trauma	0.16	-0.33, 0.65	0.5	0.6	0.19	-0.31, 0.68	0.5	0.6	0.15	-0.34, 0.64	0.5	0.7	0.18	-0.32, 0.68	0.5	0.6
age_d * age_m * baseline trauma	-0.06	-0.12, 0.01	0.09	0.2	-0.05	-0.12, 0.02	0.13	0.3	-0.06	-0.13, 0.01	0.077	0.2	-0.05	-0.12, 0.01	0.11	0.2
baseline trauma * DrkClass					-0.17	-0.53, 0.20	0.4	0.6					-0.18	-0.54, 0.18	0.3	0.5
Characteristic	Left Molecular Layer HP Head															
	Model 1				Model 2				Model 1 (controlling for Drug Use)				Model 2 (controlling for Drug Use)			
	Beta	95% CI[1]	p-value	q-value[2]	Beta	95% CI[1]	p-value	q-value[2]	Beta	95% CI[1]	p-value	q-value[2]	Beta	95% CI[1]	p-value	q-value[2]
age_d	0.57	0.24, 0.91	<0.001	**0.002**	0.56	0.21, 0.90	0.002	**0.005**	0.56	0.22, 0.90	0.001	**0.004**	0.54	0.19, 0.90	0.003	**0.008**
age_m	-0.17	-1.3, 0.97	0.8	0.8	-0.18	-1.3, 0.96	0.8	0.8	-0.17	-1.3, 0.97	0.8	0.8	-0.18	-1.3, 0.96	0.8	0.8
baseline trauma	-0.41	-2.5, 1.7	0.7	0.8	-0.37	-2.5, 1.7	0.7	0.8	-0.42	-2.5, 1.7	0.7	0.8	-0.37	-2.5, 1.7	0.7	0.8
DrkClass	0.26	-0.21, 0.72	0.3	0.4	0.33	-0.32, 0.99	0.3	0.5	0.25	-0.22, 0.72	0.3	0.4	0.33	-0.33, 0.99	0.3	0.5
wholeHippo	0.02	0.02, 0.02	<0.001	**<0.001**	0.02	0.02, 0.02	<0.001	**<0.001**	0.02	0.02, 0.02	<0.001	**<0.001**	0.02	0.02, 0.02	<0.001	**<0.001**
sex																
F	—	—			—	—			—	—			—	—		
M	17	13, 21	<0.001	**<0.001**	17	13, 21	<0.001	**<0.001**	17	12, 21	<0.001	**<0.001**	17	12, 21	<0.001	**<0.001**
ses	1.8	1.00, 2.7	<0.001	**<0.001**	1.8	1.0, 2.7	<0.001	**<0.001**	1.9	1.0, 2.7	<0.001	**<0.001**	1.9	1.0, 2.7	<0.001	**<0.001**
family alcohol density	6	1.4, 11	0.01	**0.022**	6	1.4, 11	0.01	**0.024**	6	1.4, 11	0.01	**0.026**	6	1.4, 11	0.01	**0.027**
race	5	2.7, 7.3	<0.001	**<0.001**	5	2.7, 7.3	<0.001	**<0.001**	5	2.7, 7.3	<0.001	**<0.001**	5	2.7, 7.3	<0.001	**<0.001**
LifeTob									0	0.00, 0.00	>0.9	>0.9	0	0.00, 0.00	>0.9	>0.9
LifeMJ									0	0.00, 0.01	0.7	0.8	0	0.00, 0.01	0.7	0.8
age_d * age_m	0.03	-0.10, 0.15	0.7	0.8	0.02	-0.10, 0.15	0.7	0.8	0.02	-0.10, 0.15	0.7	0.8	0.02	-0.10, 0.15	0.7	0.8
age_d * baseline trauma	0.26	0.04, 0.47	0.022	**0.041**	0.27	0.03, 0.51	0.026	0.052	0.26	0.04, 0.47	0.022	**0.048**	0.27	0.03, 0.51	0.026	0.058
age_m * baseline trauma	0.55	-0.22, 1.3	0.2	0.3	0.56	-0.21, 1.3	0.2	0.3	0.55	-0.22, 1.3	0.2	0.3	0.56	-0.21, 1.3	0.2	0.3
age_d * age_m * baseline trauma	-0.05	-0.13, 0.04	0.3	0.4	-0.04	-0.13, 0.04	0.3	0.5	-0.05	-0.13, 0.04	0.3	0.4	-0.04	-0.13, 0.04	0.3	0.5
baseline trauma * DrkClass					-0.08	-0.54, 0.39	0.7	0.8					-0.08	-0.54, 0.38	0.7	0.8
Characteristic	Right Molecular Layer HP Head															
	Model 1				Model 2				Model 1 (controlling for Drug Use)				Model 2 (controlling for Drug Use)			
	Beta	95% CI[1]	p-value	q-value[2]	Beta	95% CI[1]	p-value	q-value[2]	Beta	95% CI[1]	p-value	q-value[2]	Beta	95% CI[1]	p-value	q-value[2]

age_d	0.44	0.13, 0.76	0.006	**0.02**	0.34	0.01, 0.67	0.045	0.091	0.49	0.17, 0.81	0.003	**0.01**	0.39	0.05, 0.72	0.024	0.093
age_m	0.39	-0.67, 1.5	0.5	0.8	0.31	-0.75, 1.4	0.6	0.7	0.41	-0.66, 1.5	0.5	0.8	0.33	-0.73, 1.4	0.5	0.7
baseline trauma	-0.11	-2.1, 1.8	>0.9	>0.9	0.19	-1.8, 2.1	0.9	>0.9	-0.09	-2.0, 1.9	>0.9	>0.9	0.19	-1.8, 2.2	0.8	0.9
DrkClass	0.09	-0.36, 0.53	0.7	>0.9	0.57	-0.05, 1.2	0.071	0.12	0.11	-0.34, 0.55	0.6	0.9	0.57	-0.05, 1.2	0.069	0.14
wholeHippo	0.02	0.02, 0.02	<0.001	**<0.001**	0.02	0.02, 0.02	<0.001	**<0.001**	0.02	0.02, 0.02	<0.001	**<0.001**	0.02	0.02, 0.02	<0.001	**<0.001**
sex																
F	—	—			—	—			—	—			—	—		
M	15	11, 19	<0.001	**<0.001**	15	11, 19	<0.001	**<0.001**	15	11, 19	<0.001	**<0.001**	15	11, 19	<0.001	**<0.001**
ses	0.87	0.08, 1.7	0.032	0.084	0.88	0.09, 1.7	0.03	0.084	0.86	0.06, 1.6	0.034	0.1	0.87	0.08, 1.7	0.032	0.093
family alcohol density	4.4	0.16, 8.6	0.042	0.092	4.4	0.17, 8.6	0.042	0.091	4.4	0.17, 8.7	0.042	0.11	4.4	0.17, 8.6	0.042	0.1
race	3.8	1.7, 5.9	<0.001	**0.002**	3.8	1.7, 5.9	<0.001	**0.002**	3.9	1.8, 6.0	<0.001	**0.002**	3.9	1.8, 6.0	<0.001	**0.002**
LifeTob									0	0.00, 0.00	0.6	0.9	0	0.00, 0.00	0.6	0.7
LifeMJ									0	-0.01, 0.00	0.078	0.2	0	-0.01, 0.00	0.1	0.2
age_d * age_m	-0.05	-0.17, 0.07	0.4	0.8	-0.06	-0.18, 0.05	0.3	0.5	-0.04	-0.16, 0.08	0.5	0.8	-0.06	-0.17, 0.06	0.4	0.6
age_d * baseline trauma	-0.01	-0.22, 0.20	>0.9	>0.9	0.09	-0.14, 0.31	0.5	0.6	-0.01	-0.22, 0.19	>0.9	>0.9	0.08	-0.14, 0.31	0.5	0.7
age_m * baseline trauma	0.04	-0.68, 0.76	>0.9	>0.9	0.12	-0.60, 0.84	0.7	0.9	0.05	-0.67, 0.77	0.9	>0.9	0.13	-0.59, 0.85	0.7	0.8
age_d * age_m * baseline trauma	-0.01	-0.09, 0.07	0.8	>0.9	0	-0.08, 0.08	>0.9	>0.9	-0.01	-0.09, 0.07	0.8	>0.9	0.01	-0.08, 0.09	0.9	0.9
baseline trauma * DrkClass					-0.49	-0.92, -0.05	0.029	0.084					-0.47	-0.91, -0.03	0.035	0.093
Characteristic	Left GCMLDG Body															
	Model 1				Model 2				Model 1 (controlling for Drug Use)				Model 2 (controlling for Drug Use)			
	Beta	95% CI[1]	p-value	q-value[2]	Beta	95% CI[1]	p-value	q-value[2]	Beta	95% CI[1]	p-value	q-value[2]	Beta	95% CI[1]	p-value	q-value[2]
age_d	0.36	0.18, 0.54	<0.001	**<0.001**	0.32	0.13, 0.51	<0.001	**0.007**	0.37	0.19, 0.55	<0.001	**<0.001**	0.33	0.14, 0.52	<0.001	**0.006**
age_m	0.48	0.13, 0.82	0.007	**0.023**	0.45	0.10, 0.79	0.012	**0.042**	0.48	0.14, 0.82	0.007	**0.024**	0.45	0.10, 0.80	0.011	**0.044**
baseline trauma	-0.02	-0.64, 0.61	>0.9	>0.9	0.09	-0.55, 0.74	0.8	>0.9	-0.01	-0.64, 0.61	>0.9	>0.9	0.09	-0.55, 0.74	0.8	0.9
DrkClass	0.19	-0.05, 0.44	0.12	0.2	0.37	0.03, 0.72	0.034	0.1	0.2	-0.05, 0.45	0.12	0.3	0.37	0.03, 0.72	0.035	0.11
wholeHippo	0.01	0.01, 0.01	<0.001	**<0.001**	0.01	0.01, 0.01	<0.001	**<0.001**	0.01	0.01, 0.01	<0.001	**<0.001**	0.01	0.01, 0.01	<0.001	**<0.001**
sex																
F	—	—			—	—			—	—			—	—		
M	2.2	0.85, 3.6	0.002	**0.007**	2.2	0.85, 3.6	0.002	**0.007**	2.2	0.88, 3.6	0.001	**0.007**	2.2	0.87, 3.6	0.001	**0.007**
ses	0.21	-0.05, 0.47	0.12	0.2	0.21	-0.05, 0.47	0.11	0.2	0.21	-0.06, 0.47	0.12	0.3	0.21	-0.05, 0.47	0.12	0.3
family alcohol density	1.1	-0.29, 2.5	0.12	0.2	1.1	-0.29, 2.5	0.12	0.2	1.1	-0.29, 2.5	0.12	0.3	1.1	-0.29, 2.5	0.12	0.3
race	-0.04	-0.72, 0.64	0.9	>0.9	-0.04	-0.72, 0.64	>0.9	>0.9	-0.04	-0.72, 0.64	>0.9	>0.9	-0.04	-0.72, 0.64	>0.9	>0.9
LifeTob									0	0.00, 0.00	0.5	0.6	0	0.00, 0.00	0.5	0.7
LifeMJ									0	0.00, 0.00	0.4	0.6	0	0.00, 0.00	0.4	0.6

age_d * age_m	0.02	-0.04, 0.09	0.5	0.6	0.02	-0.05, 0.09	0.6	0.8	0.03	-0.04, 0.09	0.4	0.6	0.02	-0.05, 0.09	0.5	0.7
age_d * baseline trauma	0.05	-0.07, 0.17	0.4	0.6	0.09	-0.04, 0.22	0.2	0.3	0.05	-0.07, 0.17	0.4	0.6	0.09	-0.04, 0.22	0.2	0.3
age_m * baseline trauma	-0.02	-0.25, 0.21	0.9	>0.9	0.01	-0.22, 0.24	>0.9	>0.9	-0.02	-0.25, 0.21	0.9	>0.9	0.01	-0.22, 0.24	>0.9	>0.9
age_d * age_m * baseline trauma	-0.02	-0.06, 0.03	0.5	0.6	-0.01	-0.06, 0.03	0.6	0.8	-0.02	-0.06, 0.03	0.5	0.6	-0.01	-0.06, 0.04	0.6	0.8
baseline trauma * DrkClass					-0.18	-0.42, 0.06	0.15	0.3					-0.18	-0.42, 0.07	0.2	0.3
Characteristic	Right GCMLDG Body															
	Model 1				Model 2				Model 1 (controlling for Drug Use)				Model 2 (controlling for Drug Use)			
	Beta	95% CI[1]	p-value	q-value[2]	Beta	95% CI[1]	p-value	q-value[2]	Beta	95% CI[1]	p-value	q-value[2]	Beta	95% CI[1]	p-value	q-value[2]
age_d	0.43	0.24, 0.61	<0.001	**<0.001**	0.38	0.19, 0.58	<0.001	**<0.001**	0.41	0.22, 0.60	<0.001	**<0.001**	0.36	0.17, 0.56	<0.001	**0.002**
age_m	0.49	0.11, 0.88	0.012	**0.04**	0.46	0.07, 0.85	0.02	0.07	0.49	0.10, 0.87	0.013	**0.05**	0.45	0.07, 0.84	0.022	0.087
baseline trauma	0.13	-0.57, 0.83	0.7	>0.9	0.25	-0.46, 0.97	0.5	0.7	0.12	-0.58, 0.82	0.7	>0.9	0.25	-0.47, 0.97	0.5	0.7
DrkClass	-0.03	-0.28, 0.23	0.8	>0.9	0.18	-0.18, 0.54	0.3	0.6	-0.04	-0.29, 0.22	0.8	>0.9	0.17	-0.18, 0.53	0.3	0.6
wholeHippo	0.01	0.01, 0.01	<0.001	**<0.001**	0.01	0.01, 0.01	<0.001	**<0.001**	0.01	0.01, 0.01	<0.001	**<0.001**	0.01	0.01, 0.01	<0.001	**<0.001**
sex																
F	—	—			—	—			—	—			—	—		
M	4.1	2.6, 5.7	<0.001	**<0.001**	4.1	2.6, 5.7	<0.001	**<0.001**	4.1	2.6, 5.6	<0.001	**<0.001**	4.1	2.6, 5.6	<0.001	**<0.001**
ses	0.15	-0.15, 0.44	0.3	0.7	0.15	-0.14, 0.44	0.3	0.6	0.15	-0.14, 0.44	0.3	0.8	0.16	-0.14, 0.45	0.3	0.6
family alcohol density	0.42	-1.1, 2.0	0.6	>0.9	0.41	-1.1, 2.0	0.6	0.7	0.41	-1.1, 1.9	0.6	>0.9	0.41	-1.1, 1.9	0.6	0.7
race	0.07	-0.69, 0.83	0.9	>0.9	0.07	-0.69, 0.83	0.9	0.9	0.07	-0.70, 0.83	0.9	>0.9	0.06	-0.70, 0.83	0.9	0.9
LifeTob									0	0.00, 0.00	0.7	>0.9	0	0.00, 0.00	0.7	0.8
LifeMJ									0	0.00, 0.00	0.4	0.8	0	0.00, 0.00	0.3	0.6
age_d * age_m	-0.03	-0.10, 0.04	0.5	0.9	-0.03	-0.10, 0.04	0.4	0.6	-0.03	-0.10, 0.04	0.4	0.8	-0.03	-0.10, 0.03	0.3	0.6
age_d * baseline trauma	0	-0.12, 0.12	>0.9	>0.9	0.04	-0.09, 0.18	0.5	0.7	0	-0.12, 0.12	>0.9	>0.9	0.04	-0.09, 0.18	0.5	0.7
age_m * baseline trauma	0.01	-0.25, 0.27	>0.9	>0.9	0.04	-0.22, 0.30	0.8	0.8	0.01	-0.25, 0.26	>0.9	>0.9	0.04	-0.22, 0.30	0.8	0.8
age_d * age_m * baseline trauma	-0.03	-0.08, 0.02	0.3	0.7	-0.02	-0.07, 0.03	0.4	0.6	-0.03	-0.08, 0.02	0.2	0.7	-0.02	-0.07, 0.03	0.4	0.6
baseline trauma * DrkClass					-0.21	-0.46, 0.05	0.11	0.3					-0.21	-0.46, 0.04	0.1	0.3
Characteristic	Left GCMLDG Head															
	Model 1				Model 2				Model 1 (controlling for Drug Use)				Model 2 (controlling for Drug Use)			
	Beta	95% CI[1]	p-value	q-value[2]	Beta	95% CI[1]	p-value	q-value[2]	Beta	95% CI[1]	p-value	q-value[2]	Beta	95% CI[1]	p-value	q-value[2]
age_d	0.22	0.02, 0.42	0.031	0.08	0.24	0.03, 0.45	0.023	0.064	0.22	0.02, 0.43	0.03	0.091	0.25	0.04, 0.46	0.022	0.072
age_m	-0.13	-0.65, 0.40	0.6	0.6	-0.11	-0.64, 0.42	0.7	0.7	-0.12	-0.65, 0.40	0.6	0.7	-0.11	-0.64, 0.42	0.7	0.8
baseline trauma	0.51	-0.46, 1.5	0.3	0.4	0.45	-0.53, 1.4	0.4	0.5	0.51	-0.46, 1.5	0.3	0.5	0.45	-0.53, 1.4	0.4	0.6
DrkClass	0.14	-0.14, 0.42	0.3	0.4	0.04	-0.35, 0.43	0.8	0.8	0.14	-0.14, 0.42	0.3	0.5	0.04	-0.35, 0.43	0.9	0.9
wholeHippo	0.01	0.01, 0.01	<0.001	**<0.001**	0.01	0.01, 0.01	<0.001	**<0.001**	0.01	0.01, 0.01	<0.001	**<0.001**	0.01	0.01, 0.01	<0.001	**<0.001**

sex																
F	—	—			—	—			—	—			—	—		
M	6.7	4.6, 8.8	<0.001	**<0.001**	6.7	4.7, 8.8	<0.001	**<0.001**	6.7	4.7, 8.8	<0.001	**<0.001**	6.7	4.7, 8.8	<0.001	**<0.001**
ses	0.73	0.33, 1.1	<0.001	**0.001**	0.73	0.33, 1.1	<0.001	**0.002**	0.73	0.33, 1.1	<0.001	**0.002**	0.73	0.33, 1.1	<0.001	**0.002**
family alcohol density	0.63	-1.5, 2.7	0.6	0.6	0.63	-1.5, 2.7	0.6	0.6	0.63	-1.5, 2.7	0.6	0.7	0.63	-1.5, 2.7	0.6	0.7
race	1.3	0.22, 2.3	0.018	0.058	1.3	0.22, 2.3	0.018	0.062	1.3	0.22, 2.3	0.018	0.066	1.3	0.22, 2.3	0.018	0.07
LifeTob									0	0.00, 0.00	0.6	0.7	0	0.00, 0.00	0.6	0.7
LifeMJ									0	0.00, 0.00	0.8	0.8	0	0.00, 0.00	0.7	0.8
age_d * age_m	0.04	-0.03, 0.12	0.2	0.3	0.05	-0.03, 0.12	0.2	0.4	0.05	-0.03, 0.12	0.2	0.4	0.05	-0.03, 0.12	0.2	0.4
age_d * baseline trauma	0.1	-0.03, 0.23	0.13	0.2	0.08	-0.06, 0.22	0.3	0.4	0.1	-0.03, 0.23	0.14	0.3	0.08	-0.06, 0.22	0.3	0.5
age_m * baseline trauma	0.28	-0.08, 0.63	0.13	0.2	0.26	-0.10, 0.62	0.2	0.3	0.28	-0.08, 0.63	0.13	0.3	0.26	-0.10, 0.62	0.2	0.4
age_d * age_m * baseline trauma	-0.04	-0.09, 0.01	0.2	0.3	-0.04	-0.09, 0.01	0.13	0.3	-0.04	-0.09, 0.01	0.2	0.3	-0.04	-0.09, 0.01	0.13	0.3
baseline trauma * DrkClass					0.1	-0.17, 0.38	0.5	0.6					0.1	-0.17, 0.38	0.5	0.7

Characteristic	Right GCMLDG Head															
	Model 1				Model 2				Model 1 (controlling for Drug Use)				Model 2 (controlling for Drug Use)			
	Beta	95% CI[1]	p-value	q-value[2]	Beta	95% CI[1]	p-value	q-value[2]	Beta	95% CI[1]	p-value	q-value[2]	Beta	95% CI[1]	p-value	q-value[2]
age_d	0.32	0.13, 0.52	0.001	**0.004**	0.27	0.07, 0.47	0.008	**0.039**	0.33	0.14, 0.53	<0.001	**0.005**	0.28	0.07, 0.48	0.008	**0.042**
age_m	-0.12	-0.65, 0.41	0.7	0.7	-0.16	-0.69, 0.37	0.6	0.6	-0.12	-0.65, 0.41	0.7	0.8	-0.16	-0.69, 0.37	0.6	0.6
baseline trauma	0.37	-0.59, 1.3	0.4	0.6	0.52	-0.46, 1.5	0.3	0.4	0.38	-0.59, 1.3	0.4	0.6	0.52	-0.46, 1.5	0.3	0.5
DrkClass	0.09	-0.18, 0.35	0.5	0.6	0.34	-0.04, 0.71	0.082	0.2	0.09	-0.17, 0.36	0.5	0.6	0.34	-0.04, 0.72	0.077	0.2
wholeHippo	0.01	0.01, 0.01	<0.001	**<0.001**	0.01	0.01, 0.01	<0.001	**<0.001**	0.01	0.01, 0.01	<0.001	**<0.001**	0.01	0.01, 0.01	<0.001	**<0.001**
sex																
F	—	—			—	—			—	—			—	—		
M	7.2	5.1, 9.3	<0.001	**<0.001**	7.2	5.1, 9.3	<0.001	**<0.001**	7.3	5.2, 9.4	<0.001	**<0.001**	7.2	5.1, 9.3	<0.001	**<0.001**
ses	0.37	-0.03, 0.77	0.068	0.2	0.38	-0.02, 0.78	0.063	0.2	0.37	-0.03, 0.77	0.07	0.2	0.38	-0.02, 0.78	0.065	0.2
family alcohol density	0.78	-1.3, 2.9	0.5	0.6	0.78	-1.3, 2.9	0.5	0.6	0.79	-1.3, 2.9	0.5	0.6	0.79	-1.3, 2.9	0.5	0.6
race	1.1	-0.01, 2.1	0.052	0.2	1.1	-0.01, 2.1	0.052	0.2	1.1	0.00, 2.1	0.051	0.2	1.1	0.00, 2.1	0.051	0.2
LifeTob									0	0.00, 0.00	0.5	0.6	0	0.00, 0.00	0.5	0.6
LifeMJ									0	0.00, 0.00	0.8	0.8	0	0.00, 0.00	0.8	0.9
age_d * age_m	-0.04	-0.11, 0.03	0.3	0.5	-0.04	-0.12, 0.03	0.2	0.3	-0.04	-0.11, 0.04	0.3	0.6	-0.04	-0.12, 0.03	0.2	0.4
age_d * baseline trauma	-0.09	-0.22, 0.04	0.2	0.3	-0.04	-0.18, 0.10	0.6	0.6	-0.09	-0.22, 0.04	0.2	0.4	-0.04	-0.18, 0.10	0.6	0.6
age_m * baseline trauma	0.19	-0.17, 0.55	0.3	0.5	0.23	-0.13, 0.59	0.2	0.3	0.19	-0.17, 0.55	0.3	0.6	0.23	-0.13, 0.59	0.2	0.4
age_d * age_m * baseline trauma	-0.01	-0.05, 0.04	0.8	0.8	0	-0.05, 0.05	>0.9	>0.9	0	-0.05, 0.04	0.9	0.9	0	-0.05, 0.05	>0.9	>0.9
baseline trauma * DrkClass					-0.25	-0.51, 0.02	0.066	0.2					-0.25	-0.51, 0.02	0.07	0.2

Characteristic	Left Fimbria															
	Model 1				Model 2				Model 1 (controlling for Drug Use)				Model 2 (controlling for Drug Use)			
	Beta	95% CI[1]	p-value	q-value[2]	Beta	95% CI[1]	p-value	q-value[2]	Beta	95% CI[1]	p-value	q-value[2]	Beta	95% CI[1]	p-value	q-value[2]
age_d	0.62	0.38, 0.85	<0.001	**<0.001**	0.57	0.32, 0.81	<0.001	**<0.001**	0.64	0.40, 0.88	<0.001	**<0.001**	0.59	0.34, 0.84	<0.001	**<0.001**
age_m	0.1	-0.39, 0.58	0.7	0.8	0.06	-0.43, 0.55	0.8	0.8	0.1	-0.39, 0.59	0.7	0.9	0.07	-0.42, 0.55	0.8	0.8
baseline trauma	-0.27	-1.2, 0.62	0.5	0.8	-0.13	-1.0, 0.78	0.8	0.8	-0.26	-1.1, 0.62	0.6	0.8	-0.13	-1.0, 0.78	0.8	0.8
DrkClass	-0.02	-0.34, 0.31	>0.9	>0.9	0.21	-0.24, 0.67	0.4	0.6	0	-0.32, 0.33	>0.9	>0.9	0.22	-0.23, 0.68	0.3	0.6
wholeHippo	0	0.00, 0.00	<0.001	**<0.001**	0	0.00, 0.00	<0.001	**<0.001**	0	0.00, 0.00	<0.001	**<0.001**	0	0.00, 0.00	<0.001	**<0.001**
sex																
F	—	—			—	—			—	—			—	—		
M	8.6	6.6, 11	<0.001	**<0.001**	8.6	6.6, 10	<0.001	**<0.001**	8.6	6.7, 11	<0.001	**<0.001**	8.6	6.7, 11	<0.001	**<0.001**
ses	0.07	-0.30, 0.44	0.7	0.8	0.07	-0.30, 0.44	0.7	0.8	0.06	-0.31, 0.43	0.7	0.9	0.07	-0.30, 0.44	0.7	0.8
family alcohol density	2.3	0.40, 4.3	0.018	0.06	2.3	0.40, 4.3	0.018	0.064	2.4	0.41, 4.3	0.018	0.066	2.4	0.41, 4.3	0.018	0.071
race	0.49	-0.47, 1.5	0.3	0.8	0.49	-0.47, 1.5	0.3	0.6	0.5	-0.46, 1.5	0.3	0.7	0.5	-0.46, 1.5	0.3	0.6
LifeTob									0	0.00, 0.00	0.5	0.8	0	0.00, 0.00	0.5	0.7
LifeMJ									0	0.00, 0.00	0.3	0.7	0	0.00, 0.00	0.4	0.6
age_d * age_m	-0.01	-0.10, 0.07	0.8	0.8	-0.02	-0.11, 0.07	0.6	0.8	-0.01	-0.10, 0.08	0.8	0.9	-0.02	-0.11, 0.07	0.7	0.8
age_d * baseline trauma	0.07	-0.08, 0.23	0.3	0.8	0.12	-0.05, 0.29	0.2	0.4	0.08	-0.08, 0.23	0.3	0.7	0.12	-0.05, 0.29	0.2	0.5
age_m * baseline trauma	0.09	-0.24, 0.41	0.6	0.8	0.12	-0.21, 0.45	0.5	0.7	0.09	-0.24, 0.42	0.6	0.8	0.12	-0.21, 0.45	0.5	0.7
age_d * age_m * baseline trauma	0.02	-0.04, 0.08	0.5	0.8	0.03	-0.03, 0.09	0.4	0.6	0.02	-0.04, 0.08	0.5	0.8	0.03	-0.03, 0.09	0.4	0.6
baseline trauma * DrkClass					-0.23	-0.55, 0.09	0.2	0.4					-0.22	-0.54, 0.10	0.2	0.5

Characteristic	Right Fimbria															
	Model 1				Model 2				Model 1 (controlling for Drug Use)				Model 2 (controlling for Drug Use)			
	Beta	95% CI[1]	p-value	q-value[2]	Beta	95% CI[1]	p-value	q-value[2]	Beta	95% CI[1]	p-value	q-value[2]	Beta	95% CI[1]	p-value	q-value[2]
age_d	0.71	0.47, 0.95	<0.001	**<0.001**	0.7	0.45, 0.95	<0.001	**<0.001**	0.71	0.47, 0.96	<0.001	**<0.001**	0.7	0.45, 0.96	<0.001	**<0.001**
age_m	0.2	-0.27, 0.67	0.4	0.7	0.19	-0.28, 0.66	0.4	0.8	0.2	-0.27, 0.67	0.4	0.8	0.19	-0.28, 0.66	0.4	>0.9
baseline trauma	-0.26	-1.1, 0.59	0.5	0.7	-0.24	-1.1, 0.64	0.6	0.8	-0.26	-1.1, 0.59	0.5	0.8	-0.24	-1.1, 0.64	0.6	>0.9
DrkClass	-0.1	-0.43, 0.23	0.5	0.7	-0.06	-0.52, 0.41	0.8	0.8	-0.1	-0.43, 0.24	0.6	0.8	-0.06	-0.52, 0.41	0.8	>0.9
wholeHippo	0	0.00, 0.00	<0.001	**<0.001**	0	0.00, 0.00	<0.001	**<0.001**	0	0.00, 0.00	<0.001	**<0.001**	0	0.00, 0.00	<0.001	**<0.001**
sex																
F	—	—			—	—			—	—			—	—		
M	8.4	6.6, 10	<0.001	**<0.001**	8.4	6.6, 10	<0.001	**<0.001**	8.4	6.6, 10	<0.001	**<0.001**	8.4	6.6, 10	<0.001	**<0.001**
ses	0.05	-0.30, 0.41	0.8	0.8	0.05	-0.30, 0.41	0.8	0.8	0.05	-0.30, 0.40	0.8	>0.9	0.05	-0.30, 0.41	0.8	>0.9
family alcohol density	0.48	-1.4, 2.3	0.6	0.7	0.48	-1.4, 2.3	0.6	0.8	0.48	-1.4, 2.3	0.6	0.8	0.48	-1.4, 2.3	0.6	>0.9

race	0.56	-0.36, 1.5	0.2	0.5	0.56	-0.36, 1.5	0.2	0.5	0.56	-0.36, 1.5	0.2	0.6	0.56	-0.36, 1.5	0.2	0.6
LifeTob									0	0.00, 0.00	>0.9	>0.9	0	0.00, 0.00	>0.9	>0.9
LifeMJ									0	0.00, 0.00	0.9	>0.9	0	0.00, 0.00	0.9	>0.9
age_d * age_m	-0.09	-0.18, 0.00	0.058	0.2	-0.09	-0.18, 0.00	0.056	0.2	-0.09	-0.18, 0.00	0.061	0.2	-0.09	-0.18, 0.00	0.059	0.2
age_d * baseline trauma	-0.04	-0.20, 0.12	0.6	0.7	-0.03	-0.20, 0.14	0.7	0.8	-0.04	-0.20, 0.12	0.6	0.8	-0.03	-0.20, 0.14	0.7	>0.9
age_m * baseline trauma	-0.07	-0.38, 0.24	0.7	0.7	-0.06	-0.38, 0.25	0.7	0.8	-0.07	-0.38, 0.24	0.7	0.8	-0.06	-0.38, 0.26	0.7	>0.9
age_d * age_m * baseline trauma	0.07	0.00, 0.13	0.035	0.11	0.07	0.01, 0.13	0.034	0.12	0.07	0.00, 0.13	0.034	0.13	0.07	0.01, 0.13	0.033	0.13
baseline trauma * DrkClass					-0.04	-0.37, 0.28	0.8	0.8					-0.04	-0.37, 0.28	0.8	>0.9
Characteristic	Left HATA															
	Model 1				Model 2				Model 1 (controlling for Drug Use)				Model 2 (controlling for Drug Use)			
	Beta	95% CI[1]	p-value	q-value[2]	Beta	95% CI[1]	p-value	q-value[2]	Beta	95% CI[1]	p-value	q-value[2]	Beta	95% CI[1]	p-value	q-value[2]
age_d	0.21	0.10, 0.33	<0.001	**<0.001**	0.24	0.12, 0.36	<0.001	**<0.001**	0.21	0.10, 0.33	<0.001	**0.001**	0.24	0.12, 0.36	<0.001	**<0.001**
age_m	0.32	0.03, 0.61	0.029	0.075	0.34	0.05, 0.63	0.022	0.062	0.32	0.03, 0.61	0.03	0.085	0.34	0.05, 0.63	0.022	0.071
baseline trauma	0.2	-0.33, 0.73	0.5	0.6	0.13	-0.41, 0.67	0.6	0.9	0.2	-0.33, 0.73	0.5	0.6	0.13	-0.41, 0.67	0.6	>0.9
DrkClass	0.07	-0.09, 0.23	0.4	0.6	-0.04	-0.27, 0.18	0.7	0.9	0.08	-0.08, 0.23	0.3	0.5	-0.04	-0.26, 0.18	0.7	>0.9
wholeHippo	0	0.00, 0.00	<0.001	**<0.001**	0	0.00, 0.00	<0.001	**<0.001**	0	0.00, 0.00	<0.001	**<0.001**	0	0.00, 0.00	<0.001	**<0.001**
sex																
F	—	—			—	—			—	—			—	—		
M	3	1.9, 4.2	<0.001	**<0.001**	3	1.9, 4.2	<0.001	**<0.001**	3	1.9, 4.2	<0.001	**<0.001**	3	1.9, 4.2	<0.001	**<0.001**
ses	0.15	-0.06, 0.37	0.2	0.3	0.15	-0.07, 0.37	0.2	0.3	0.15	-0.07, 0.37	0.2	0.3	0.15	-0.07, 0.37	0.2	0.3
family alcohol density	1.3	0.09, 2.4	0.034	0.075	1.3	0.09, 2.4	0.035	0.081	1.3	0.10, 2.4	0.034	0.085	1.3	0.10, 2.4	0.034	0.09
race	1.3	0.69, 1.8	<0.001	**<0.001**	1.3	0.69, 1.8	<0.001	**<0.001**	1.3	0.69, 1.8	<0.001	**<0.001**	1.3	0.69, 1.8	<0.001	**<0.001**
LifeTob									0	0.00, 0.00	0.2	0.3	0	0.00, 0.00	0.2	0.3
LifeMJ									0	0.00, 0.00	>0.9	>0.9	0	0.00, 0.00	>0.9	>0.9
age_d * age_m	0	-0.04, 0.04	>0.9	>0.9	0.01	-0.04, 0.05	0.8	0.9	0	-0.04, 0.04	>0.9	>0.9	0.01	-0.04, 0.05	0.8	>0.9
age_d * baseline trauma	-0.05	-0.12, 0.03	0.2	0.4	-0.07	-0.15, 0.01	0.095	0.2	-0.05	-0.12, 0.03	0.2	0.4	-0.07	-0.15, 0.01	0.1	0.2
age_m * baseline trauma	0	-0.19, 0.20	>0.9	>0.9	-0.01	-0.21, 0.18	0.9	0.9	0	-0.19, 0.20	>0.9	>0.9	-0.01	-0.21, 0.18	0.9	>0.9
age_d * age_m * baseline trauma	0	-0.03, 0.03	>0.9	>0.9	0	-0.03, 0.03	0.8	0.9	0	-0.03, 0.03	>0.9	>0.9	0	-0.03, 0.03	0.8	>0.9
baseline trauma * DrkClass					0.11	-0.04, 0.27	0.2	0.3					0.12	-0.04, 0.28	0.2	0.3
Characteristic	Right HATA															
	Model 1				Model 2				Model 1 (controlling for Drug Use)				Model 2 (controlling for Drug Use)			
	Beta	95% CI[1]	p-value	q-value[2]	Beta	95% CI[1]	p-value	q-value[2]	Beta	95% CI[1]	p-value	q-value[2]	Beta	95% CI[1]	p-value	q-value[2]
age_d	0.18	0.05, 0.30	0.006	**0.019**	0.17	0.04, 0.30	0.012	**0.044**	0.2	0.08, 0.33	0.002	**0.007**	0.2	0.06, 0.33	0.004	**0.016**
age_m	0.37	0.05, 0.70	0.024	0.062	0.37	0.04, 0.69	0.027	0.075	0.39	0.06, 0.71	0.02	**0.05**	0.38	0.05, 0.71	0.022	0.059

baseline trauma	0.31	-0.28, 0.91	0.3	0.6	0.34	-0.26, 0.94	0.3	0.5	0.32	-0.27, 0.91	0.3	0.5	0.34	-0.26, 0.94	0.3	0.6
DrkClass	0.05	-0.12, 0.23	0.5	0.7	0.1	-0.15, 0.34	0.4	0.6	0.07	-0.11, 0.24	0.4	0.6	0.1	-0.14, 0.35	0.4	0.6
wholeHippo	0	0.00, 0.01	<0.001	**<0.001**	0	0.00, 0.01	<0.001	**<0.001**	0	0.00, 0.01	<0.001	**<0.001**	0	0.00, 0.01	<0.001	**<0.001**
sex																
F	—	—			—	—			—	—			—	—		
M	3	1.7, 4.3	<0.001	**<0.001**	3	1.7, 4.3	<0.001	**<0.001**	3.1	1.8, 4.4	<0.001	**<0.001**	3.1	1.8, 4.4	<0.001	**<0.001**
ses	-0.03	-0.27, 0.22	0.8	0.9	-0.03	-0.27, 0.22	0.8	0.9	-0.03	-0.28, 0.21	0.8	0.8	-0.03	-0.28, 0.21	0.8	0.8
family alcohol density	0.57	-0.74, 1.9	0.4	0.6	0.57	-0.74, 1.9	0.4	0.6	0.57	-0.73, 1.9	0.4	0.6	0.57	-0.73, 1.9	0.4	0.6
race	1.5	0.81, 2.1	<0.001	**<0.001**	1.5	0.81, 2.1	<0.001	**<0.001**	1.5	0.82, 2.1	<0.001	**<0.001**	1.5	0.82, 2.1	<0.001	**<0.001**
LifeTob									0	0.00, 0.00	0.8	0.8	0	0.00, 0.00	0.8	0.8
LifeMJ									0	0.00, 0.00	0.016	**0.048**	0	0.00, 0.00	0.017	0.055
age_d * age_m	0.02	-0.03, 0.07	0.4	0.6	0.02	-0.03, 0.07	0.4	0.6	0.02	-0.02, 0.07	0.3	0.5	0.02	-0.02, 0.07	0.3	0.6
age_d * baseline trauma	-0.02	-0.11, 0.06	0.6	0.7	-0.01	-0.10, 0.07	0.7	0.9	-0.02	-0.11, 0.06	0.6	0.7	-0.02	-0.11, 0.07	0.7	0.8
age_m * baseline trauma	0	-0.22, 0.22	>0.9	>0.9	0	-0.22, 0.23	>0.9	>0.9	0	-0.22, 0.22	>0.9	>0.9	0.01	-0.21, 0.23	>0.9	>0.9
age_d * age_m * baseline trauma	-0.02	-0.05, 0.01	0.2	0.5	-0.02	-0.05, 0.01	0.3	0.5	-0.02	-0.05, 0.01	0.3	0.5	-0.02	-0.05, 0.02	0.3	0.6
baseline trauma * DrkClass					-0.04	-0.22, 0.13	0.6	0.8					-0.03	-0.21, 0.14	0.7	0.8

Characteristic	Left Whole Hippocampus Body															
	Model 1				Model 2				Model 1 (controlling for Drug Use)				Model 2 (controlling for Drug Use)			
	Beta	95% CI[1]	p-value	q-value[2]	Beta	95% CI[1]	p-value	q-value[2]	Beta	95% CI[1]	p-value	q-value[2]	Beta	95% CI[1]	p-value	q-value[2]
age_d	3.7	2.6, 4.7	<0.001	**<0.001**	3.4	2.3, 4.6	<0.001	**<0.001**	3.6	2.5, 4.7	<0.001	**<0.001**	3.4	2.2, 4.5	<0.001	**<0.001**
age_m	5.5	2.4, 8.6	<0.001	**0.002**	5.3	2.2, 8.4	<0.001	**0.003**	5.5	2.4, 8.6	<0.001	**0.002**	5.3	2.2, 8.4	<0.001	**0.004**
baseline trauma	-0.12	-5.8, 5.5	>0.9	>0.9	0.54	-5.2, 6.2	0.9	0.9	-0.14	-5.8, 5.5	>0.9	>0.9	0.53	-5.2, 6.2	0.9	0.9
DrkClass	0.88	-0.62, 2.4	0.3	0.4	2	-0.16, 4.1	0.07	0.12	0.84	-0.67, 2.4	0.3	0.5	1.9	-0.18, 4.1	0.074	0.15
wholeHippo	0.06	0.05, 0.06	<0.001	**<0.001**	0.06	0.05, 0.07	<0.001	**<0.001**	0.06	0.05, 0.06	<0.001	**<0.001**	0.06	0.05, 0.07	<0.001	**<0.001**
sex																
F	—	—			—	—			—	—			—	—		
M	51	39, 63	<0.001	**<0.001**	51	39, 63	<0.001	**<0.001**	51	38, 63	<0.001	**<0.001**	51	38, 63	<0.001	**<0.001**
ses	3.2	0.90, 5.6	0.007	**0.017**	3.2	0.92, 5.6	0.006	**0.018**	3.2	0.92, 5.6	0.006	**0.019**	3.3	0.94, 5.6	0.006	**0.019**
family alcohol density	14	1.8, 27	0.025	0.053	14	1.9, 27	0.025	0.057	14	1.8, 27	0.025	0.062	14	1.8, 27	0.025	0.066
race	6.7	0.60, 13	0.032	0.059	6.7	0.59, 13	0.032	0.064	6.7	0.57, 13	0.033	0.07	6.7	0.55, 13	0.033	0.076
LifeTob									0	0.00, 0.01	0.8	0.9	0	0.00, 0.01	0.8	0.9
LifeMJ									0	-0.01, 0.02	0.6	0.7	0	-0.01, 0.02	0.5	0.7
age_d * age_m	0.08	-0.32, 0.48	0.7	0.8	0.05	-0.36, 0.45	0.8	0.9	0.07	-0.33, 0.47	0.7	0.8	0.04	-0.37, 0.44	0.9	0.9
age_d * baseline trauma	0.37	-0.34, 1.1	0.3	0.4	0.58	-0.19, 1.4	0.14	0.2	0.36	-0.35, 1.1	0.3	0.5	0.58	-0.19, 1.4	0.14	0.2

age_m * baseline trauma	-0.98	-3.1, 1.1	0.4	0.4	-0.81	-2.9, 1.3	0.5	0.5	-0.99	-3.1, 1.1	0.4	0.5	-0.82	-2.9, 1.3	0.4	0.6
age_d * age_m * baseline trauma	-0.18	-0.46, 0.09	0.2	0.3	-0.15	-0.43, 0.13	0.3	0.4	-0.19	-0.47, 0.09	0.2	0.3	-0.15	-0.44, 0.13	0.3	0.4
baseline trauma * DrkClass					-1.1	-2.6, 0.42	0.2	0.2					-1.1	-2.6, 0.40	0.15	0.2
Characteristic	Right Whole Hippocampus Body															
	Model 1				Model 2				Model 1 (controlling for Drug Use)				Model 2 (controlling for Drug Use)			
	Beta	95% CI[1]	p-value	q-value[2]	Beta	95% CI[1]	p-value	q-value[2]	Beta	95% CI[1]	p-value	q-value[2]	Beta	95% CI[1]	p-value	q-value[2]
age_d	3.8	2.7, 5.0	<0.001	**<0.001**	3.7	2.5, 4.8	<0.001	**<0.001**	3.7	2.6, 4.8	<0.001	**<0.001**	3.5	2.3, 4.7	<0.001	**<0.001**
age_m	4.9	2.0, 7.8	0.001	**0.003**	4.8	1.8, 7.7	0.001	**0.005**	4.9	1.9, 7.8	0.001	**0.004**	4.7	1.8, 7.6	0.002	**0.007**
baseline trauma	4.8	-0.56, 10	0.08	0.2	5.3	-0.14, 11	0.057	0.2	4.7	-0.61, 10	0.083	0.2	5.2	-0.15, 11	0.057	0.2
DrkClass	0.16	-1.4, 1.7	0.8	0.9	0.99	-1.2, 3.2	0.4	0.5	0.05	-1.5, 1.6	>0.9	>0.9	0.94	-1.2, 3.1	0.4	0.6
wholeHippo	0.06	0.06, 0.07	<0.001	**<0.001**	0.06	0.06, 0.07	<0.001	**<0.001**	0.06	0.06, 0.07	<0.001	**<0.001**	0.07	0.06, 0.07	<0.001	**<0.001**
sex																
F	—	—			—	—			—	—			—	—		
M	53	41, 64	<0.001	**<0.001**	53	41, 64	<0.001	**<0.001**	52	41, 64	<0.001	**<0.001**	52	41, 64	<0.001	**<0.001**
ses	1.6	-0.64, 3.7	0.2	0.3	1.6	-0.62, 3.8	0.2	0.3	1.6	-0.59, 3.8	0.2	0.3	1.6	-0.57, 3.8	0.15	0.3
family alcohol density	2.5	-9.1, 14	0.7	0.8	2.5	-9.1, 14	0.7	0.8	2.5	-9.1, 14	0.7	0.8	2.5	-9.1, 14	0.7	0.8
race	4.8	-0.97, 11	0.1	0.2	4.8	-0.98, 11	0.1	0.2	4.7	-1.0, 10	0.11	0.2	4.7	-1.0, 10	0.11	0.3
LifeTob									0	0.00, 0.01	0.7	0.8	0	0.00, 0.01	0.7	0.8
LifeMJ									0.01	0.00, 0.03	0.2	0.3	0.01	0.00, 0.03	0.14	0.3
age_d * age_m	-0.1	-0.52, 0.32	0.6	0.8	-0.12	-0.54, 0.29	0.6	0.7	-0.12	-0.54, 0.30	0.6	0.8	-0.15	-0.57, 0.27	0.5	0.7
age_d * baseline trauma	-0.18	-0.91, 0.56	0.6	0.8	-0.01	-0.81, 0.79	>0.9	>0.9	-0.18	-0.92, 0.55	0.6	0.8	-0.01	-0.80, 0.79	>0.9	>0.9
age_m * baseline trauma	-0.19	-2.1, 1.8	0.9	0.9	-0.05	-2.0, 1.9	>0.9	>0.9	-0.22	-2.2, 1.7	0.8	0.9	-0.08	-2.0, 1.9	>0.9	>0.9
age_d * age_m * baseline trauma	-0.23	-0.52, 0.05	0.11	0.2	-0.21	-0.50, 0.08	0.2	0.3	-0.24	-0.53, 0.04	0.1	0.2	-0.22	-0.51, 0.07	0.15	0.3
baseline trauma * DrkClass					-0.83	-2.4, 0.71	0.3	0.5					-0.89	-2.4, 0.65	0.3	0.4
Characteristic	Left Whole Hippocampus Head															
	Model 1				Model 2				Model 1 (controlling for Drug Use)				Model 2 (controlling for Drug Use)			
	Beta	95% CI[1]	p-value	q-value[2]	Beta	95% CI[1]	p-value	q-value[2]	Beta	95% CI[1]	p-value	q-value[2]	Beta	95% CI[1]	p-value	q-value[2]
age_d	2.2	0.73, 3.7	0.004	**0.009**	2.2	0.61, 3.7	0.006	**0.018**	2.2	0.71, 3.7	0.004	**0.012**	2.2	0.59, 3.8	0.007	**0.024**
age_m	-0.16	-5.4, 5.1	>0.9	>0.9	-0.21	-5.5, 5.0	>0.9	>0.9	-0.17	-5.4, 5.1	>0.9	>0.9	-0.21	-5.5, 5.0	>0.9	>0.9
baseline trauma	-0.18	-9.7, 9.4	>0.9	>0.9	-0.02	-9.7, 9.6	>0.9	>0.9	-0.19	-9.7, 9.4	>0.9	>0.9	-0.03	-9.7, 9.6	>0.9	>0.9
DrkClass	1.5	-0.58, 3.6	0.2	0.2	1.8	-1.2, 4.7	0.2	0.3	1.5	-0.57, 3.6	0.2	0.3	1.8	-1.2, 4.7	0.2	0.4
wholeHippo	0.09	0.08, 0.10	<0.001	**<0.001**	0.09	0.08, 0.10	<0.001	**<0.001**	0.09	0.08, 0.10	<0.001	**<0.001**	0.09	0.08, 0.10	<0.001	**<0.001**
sex																
F	—	—			—	—			—	—			—	—		

M	110	90, 131	<0.001	**<0.001**	110	90, 131	<0.001	**<0.001**	110	90, 131	<0.001	**<0.001**	110	90, 131	<0.001	**<0.001**
ses	10	6.2, 14	<0.001	**<0.001**	10	6.2, 14	<0.001	**<0.001**	10	6.2, 14	<0.001	**<0.001**	10	6.2, 14	<0.001	**<0.001**
family alcohol density	25	3.9, 46	0.02	**0.044**	25	3.9, 46	0.02	**0.047**	25	3.9, 46	0.02	**0.05**	25	3.9, 46	0.02	0.053
race	29	19, 40	<0.001	**<0.001**	29	19, 40	<0.001	**<0.001**	29	19, 40	<0.001	**<0.001**	29	19, 40	<0.001	**<0.001**
LifeTob									0	-0.01, 0.00	0.7	0.9	0	-0.01, 0.00	0.7	>0.9
LifeMJ									0	-0.02, 0.02	>0.9	>0.9	0	-0.02, 0.02	>0.9	>0.9
age_d * age_m	0.21	-0.35, 0.76	0.5	0.5	0.2	-0.36, 0.76	0.5	0.6	0.21	-0.35, 0.76	0.5	0.6	0.2	-0.36, 0.76	0.5	0.7
age_d * baseline trauma	1.1	0.12, 2.1	0.028	0.053	1.1	0.09, 2.2	0.034	0.069	1.1	0.12, 2.1	0.028	0.06	1.2	0.09, 2.2	0.034	0.078
age_m * baseline trauma	3	-0.55, 6.5	0.1	0.2	3	-0.52, 6.6	0.1	0.2	3	-0.55, 6.5	0.1	0.2	3	-0.52, 6.6	0.1	0.2
age_d * age_m * baseline trauma	-0.25	-0.63, 0.13	0.2	0.3	-0.24	-0.63, 0.14	0.2	0.3	-0.25	-0.63, 0.13	0.2	0.3	-0.24	-0.63, 0.14	0.2	0.4
baseline trauma * DrkClass					-0.27	-2.3, 1.8	0.8	>0.9					-0.26	-2.3, 1.8	0.8	>0.9
Characteristic	Right Whole Hippocampus Head															
	Model 1				Model 2				Model 1 (controlling for Drug Use)				Model 2 (controlling for Drug Use)			
	Beta	95% CI[1]	p-value	q-value[2]	Beta	95% CI[1]	p-value	q-value[2]	Beta	95% CI[1]	p-value	q-value[2]	Beta	95% CI[1]	p-value	q-value[2]
age_d	1.6	0.22, 3.0	0.024	0.062	1.1	-0.40, 2.5	0.2	0.3	1.9	0.47, 3.3	0.009	**0.027**	1.3	-0.14, 2.8	0.076	0.2
age_m	2.7	-2.3, 7.8	0.3	0.5	2.3	-2.7, 7.4	0.4	0.4	2.9	-2.2, 7.9	0.3	0.5	2.5	-2.6, 7.5	0.3	0.4
baseline trauma	2.8	-6.5, 12	0.6	0.7	4.3	-5.0, 14	0.4	0.4	2.8	-6.4, 12	0.5	0.7	4.4	-5.0, 14	0.4	0.4
DrkClass	0.67	-1.3, 2.6	0.5	0.7	3.3	0.51, 6.0	0.02	**0.047**	0.78	-1.2, 2.7	0.4	0.7	3.3	0.52, 6.0	0.02	0.053
wholeHippo	0.09	0.08, 0.10	<0.001	**<0.001**	0.09	0.08, 0.10	<0.001	**<0.001**	0.09	0.08, 0.10	<0.001	**<0.001**	0.09	0.08, 0.10	<0.001	**<0.001**
sex																
F	—	—			—	—			—	—			—	—		
M	103	84, 123	<0.001	**<0.001**	103	83, 122	<0.001	**<0.001**	104	85, 124	<0.001	**<0.001**	104	84, 124	<0.001	**<0.001**
ses	5.7	1.9, 9.4	0.003	**0.01**	5.7	2.0, 9.5	0.003	**0.009**	5.6	1.9, 9.4	0.003	**0.013**	5.7	2.0, 9.4	0.003	**0.012**
family alcohol density	16	-3.9, 36	0.11	0.2	16	-3.8, 36	0.11	0.2	16	-3.8, 36	0.11	0.2	16	-3.8, 36	0.11	0.2
race	24	14, 34	<0.001	**<0.001**	24	14, 34	<0.001	**<0.001**	25	15, 35	<0.001	**<0.001**	25	14, 35	<0.001	**<0.001**
LifeTob									0	0.00, 0.01	0.5	0.7	0	0.00, 0.01	0.5	0.5
LifeMJ									-0.02	-0.04, 0.00	0.023	0.059	-0.02	-0.04, 0.00	0.031	0.071
age_d * age_m	-0.22	-0.74, 0.30	0.4	0.7	-0.3	-0.82, 0.22	0.3	0.4	-0.17	-0.69, 0.35	0.5	0.7	-0.25	-0.77, 0.27	0.3	0.4
age_d * baseline trauma	0	-0.91, 0.92	>0.9	>0.9	0.52	-0.47, 1.5	0.3	0.4	0	-0.91, 0.92	>0.9	>0.9	0.5	-0.49, 1.5	0.3	0.4
age_m * baseline trauma	0.26	-3.1, 3.7	0.9	>0.9	0.67	-2.7, 4.1	0.7	0.8	0.32	-3.1, 3.7	0.9	>0.9	0.71	-2.7, 4.1	0.7	0.7
age_d * age_m * baseline trauma	-0.09	-0.45, 0.27	0.6	0.7	-0.01	-0.37, 0.35	>0.9	>0.9	-0.07	-0.43, 0.28	0.7	0.8	0	-0.36, 0.37	>0.9	>0.9
baseline trauma * DrkClass					-2.6	-4.5, -0.66	0.009	**0.025**					-2.5	-4.4, -0.56	0.012	**0.037**
Characteristic	Left Whole Hippocampus															
	Model 1				Model 2				Model 1 (controlling for Drug Use)				Model 2 (controlling for Drug Use)			

	Beta	95% CI[1]	p-value	q-value[2]	Beta	95% CI[1]	p-value	q-value[2]	Beta	95% CI[1]	p-value	q-value[2]	Beta	95% CI[1]	p-value	q-value[2]
age_d	5.5	3.1, 7.8	<0.001	**<0.001**	5.6	3.2, 8.0	<0.001	**<0.001**	5.5	3.2, 7.9	<0.001	**<0.001**	5.7	3.2, 8.1	<0.001	**<0.001**
age_m	0.25	-9.7, 10	>0.9	>0.9	0.35	-9.7, 10	>0.9	>0.9	0.27	-9.7, 10	>0.9	>0.9	0.38	-9.6, 10	>0.9	>0.9
baseline trauma	-6.4	-25, 12	0.5	0.6	-6.8	-25, 11	0.5	0.6	-6.4	-25, 12	0.5	0.7	-6.8	-25, 11	0.5	0.7
DrkClass	-0.9	-4.1, 2.3	0.6	0.6	-1.5	-6.1, 3.0	0.5	0.6	-0.88	-4.1, 2.4	0.6	0.7	-1.5	-6.1, 3.0	0.5	0.7
wholeHippo	0	0.00, 0.00	<0.001	**<0.001**	0	0.00, 0.00	<0.001	**<0.001**	0	0.00, 0.00	<0.001	**<0.001**	0	0.00, 0.00	<0.001	**<0.001**
sex																
F	—	—			—	—			—	—			—	—		
M	120	77, 163	<0.001	**<0.001**	120	77, 163	<0.001	**<0.001**	120	78, 163	<0.001	**<0.001**	120	78, 163	<0.001	**<0.001**
ses	14	6.4, 21	<0.001	**<0.001**	14	6.4, 21	<0.001	**<0.001**	14	6.4, 21	<0.001	**<0.001**	14	6.4, 21	<0.001	**0.001**
family alcohol density	38	-0.98, 78	0.056	0.12	38	-0.99, 78	0.056	0.13	38	-0.96, 78	0.056	0.14	38	-0.97, 78	0.056	0.15
race	20	-0.07, 40	0.051	0.12	20	-0.08, 40	0.051	0.13	20	-0.05, 40	0.051	0.14	20	-0.05, 40	0.051	0.15
LifeTob									0	-0.01, 0.01	>0.9	>0.9	0	-0.01, 0.01	>0.9	>0.9
LifeMJ									0	-0.04, 0.03	0.8	>0.9	0	-0.04, 0.03	0.8	0.9
age_d * age_m	0.43	-0.42, 1.3	0.3	0.5	0.45	-0.41, 1.3	0.3	0.5	0.44	-0.42, 1.3	0.3	0.5	0.46	-0.40, 1.3	0.3	0.5
age_d * baseline trauma	1.2	-0.33, 2.7	0.13	0.2	1.1	-0.59, 2.7	0.2	0.4	1.2	-0.33, 2.7	0.13	0.3	1.1	-0.60, 2.7	0.2	0.4
age_m * baseline trauma	2.5	-4.2, 9.2	0.5	0.6	2.4	-4.3, 9.2	0.5	0.6	2.5	-4.2, 9.3	0.5	0.7	2.4	-4.3, 9.2	0.5	0.7
age_d * age_m * baseline trauma	-0.42	-1.0, 0.17	0.2	0.3	-0.44	-1.0, 0.15	0.15	0.3	-0.42	-1.0, 0.17	0.2	0.3	-0.44	-1.0, 0.16	0.15	0.3
baseline trauma * DrkClass					0.64	-2.6, 3.9	0.7	0.7					0.66	-2.6, 3.9	0.7	0.8
Characteristic	Right Whole Hippocampus															
	Model 1				Model 2				Model 1 (controlling for Drug Use)				Model 2 (controlling for Drug Use)			
	Beta	95% CI[1]	p-value	q-value[2]	Beta	95% CI[1]	p-value	q-value[2]	Beta	95% CI[1]	p-value	q-value[2]	Beta	95% CI[1]	p-value	q-value[2]
age_d	4.5	2.2, 6.8	<0.001	**<0.001**	4.1	1.7, 6.5	<0.001	**0.003**	4.8	2.5, 7.1	<0.001	**<0.001**	4.4	2.0, 6.9	<0.001	**0.002**
age_m	4.1	-6.0, 14	0.4	0.6	3.8	-6.2, 14	0.5	0.7	4.2	-5.8, 14	0.4	0.5	3.9	-6.1, 14	0.4	0.6
baseline trauma	-1.8	-20, 16	0.8	>0.9	-0.74	-19, 18	>0.9	>0.9	-1.7	-20, 17	0.9	>0.9	-0.7	-19, 18	>0.9	>0.9
DrkClass	-2.4	-5.6, 0.84	0.15	0.3	-0.64	-5.2, 3.9	0.8	>0.9	-2.3	-5.5, 0.93	0.2	0.3	-0.65	-5.2, 3.9	0.8	0.9
wholeHippo	0	0.00, 0.00	<0.001	**<0.001**	0	0.00, 0.00	<0.001	**<0.001**	0	0.00, 0.00	<0.001	**<0.001**	0	0.00, 0.00	<0.001	**<0.001**
sex																
F	—	—			—	—			—	—			—	—		
M	115	72, 158	<0.001	**<0.001**	115	72, 158	<0.001	**<0.001**	115	73, 158	<0.001	**<0.001**	116	73, 158	<0.001	**<0.001**
ses	5.8	-1.5, 13	0.12	0.3	5.9	-1.4, 13	0.11	0.3	5.8	-1.5, 13	0.12	0.3	5.8	-1.5, 13	0.12	0.4
family alcohol density	20	-20, 59	0.3	0.5	20	-20, 59	0.3	0.6	20	-20, 59	0.3	0.5	20	-20, 59	0.3	0.5
race	16	-4.0, 36	0.12	0.3	16	-4.0, 36	0.12	0.3	16	-3.9, 36	0.11	0.3	16	-3.9, 36	0.11	0.4
LifeTob									0	0.00, 0.01	0.3	0.5	0	0.00, 0.01	0.3	0.5

LifeMJ									-0.03	-0.06, 0.01	0.13	0.3	-0.03	-0.06, 0.01	0.14	0.4
age_d * age_m	-0.14	-0.99, 0.71	0.7	0.9	-0.19	-1.0, 0.66	0.7	>0.9	-0.09	-0.94, 0.76	0.8	>0.9	-0.14	-1.00, 0.72	0.8	0.9
age_d * baseline trauma	-0.59	-2.1, 0.92	0.4	0.6	-0.24	-1.9, 1.4	0.8	>0.9	-0.6	-2.1, 0.91	0.4	0.5	-0.26	-1.9, 1.4	0.8	0.9
age_m * baseline trauma	0.36	-6.4, 7.1	>0.9	>0.9	0.63	-6.1, 7.4	0.9	>0.9	0.42	-6.3, 7.1	>0.9	>0.9	0.68	-6.1, 7.4	0.8	0.9
age_d * age_m * baseline trauma	-0.37	-0.96, 0.21	0.2	0.4	-0.32	-0.91, 0.28	0.3	0.6	-0.35	-0.94, 0.23	0.2	0.4	-0.3	-0.90, 0.29	0.3	0.5
baseline trauma * DrkClass					-1.7	-4.9, 1.4	0.3	0.6					-1.7	-4.8, 1.5	0.3	0.5

Characteristic	Left Lateral															
	Model 1				Model 2				Model 1 (controlling for Drug Use)				Model 2 (controlling for Drug Use)			
	Beta	95% CI[1]	p-value	q-value[2]	Beta	95% CI[1]	p-value	q-value[2]	Beta	95% CI[1]	p-value	q-value[2]	Beta	95% CI[1]	p-value	q-value[2]
age_d	0.85	0.10, 1.6	0.027	0.07	0.72	-0.07, 1.5	0.073	0.2	0.85	0.09, 1.6	0.029	0.088	0.72	-0.08, 1.5	0.079	0.2
age_m	1.5	-0.38, 3.5	0.12	0.2	1.4	-0.48, 3.4	0.14	0.3	1.5	-0.39, 3.5	0.12	0.3	1.4	-0.49, 3.4	0.14	0.3
baseline trauma	-1.7	-5.2, 1.8	0.3	0.5	-1.4	-4.9, 2.2	0.5	0.6	-1.7	-5.2, 1.8	0.3	0.6	-1.4	-4.9, 2.2	0.5	0.7
DrkClass	0.06	-0.98, 1.1	>0.9	>0.9	0.67	-0.80, 2.1	0.4	0.5	0.07	-0.98, 1.1	0.9	>0.9	0.67	-0.79, 2.1	0.4	0.6
wholeHippo	0.08	0.07, 0.09	<0.001	**<0.001**	0.08	0.07, 0.09	<0.001	**<0.001**	0.08	0.07, 0.09	<0.001	**<0.001**	0.08	0.07, 0.09	<0.001	**<0.001**
sex																
F	—	—			—	—			—	—			—	—		
M	40	32, 47	<0.001	**<0.001**	40	32, 47	<0.001	**<0.001**	40	32, 47	<0.001	**<0.001**	40	32, 47	<0.001	**<0.001**
ses	1.7	0.21, 3.1	0.025	0.07	1.7	0.23, 3.1	0.023	0.081	1.6	0.21, 3.1	0.025	0.088	1.7	0.23, 3.1	0.023	0.093
family alcohol density	5.7	-2.0, 13	0.15	0.2	5.7	-2.0, 13	0.15	0.3	5.7	-2.0, 13	0.15	0.3	5.7	-2.0, 13	0.15	0.3
race	6.3	2.5, 10	0.001	**0.005**	6.3	2.5, 10	0.001	**0.005**	6.3	2.5, 10	0.001	**0.006**	6.3	2.5, 10	0.001	**0.006**
LifeTob									0	0.00, 0.00	0.7	>0.9	0	0.00, 0.00	0.7	0.8
LifeMJ									0	-0.01, 0.01	>0.9	>0.9	0	-0.01, 0.01	>0.9	>0.9
age_d * age_m	-0.04	-0.32, 0.24	0.8	>0.9	-0.05	-0.34, 0.23	0.7	0.8	-0.04	-0.32, 0.24	0.8	>0.9	-0.06	-0.34, 0.23	0.7	0.8
age_d * baseline trauma	0.47	-0.02, 0.97	0.062	0.13	0.59	0.06, 1.1	0.03	0.085	0.47	-0.02, 0.97	0.061	0.2	0.6	0.06, 1.1	0.03	0.1
age_m * baseline trauma	-0.02	-1.3, 1.3	>0.9	>0.9	0.07	-1.2, 1.4	>0.9	>0.9	-0.02	-1.3, 1.3	>0.9	>0.9	0.07	-1.2, 1.4	>0.9	>0.9
age_d * age_m * baseline trauma	0.02	-0.18, 0.21	0.9	>0.9	0.04	-0.16, 0.23	0.7	0.8	0.02	-0.18, 0.21	0.9	>0.9	0.04	-0.16, 0.23	0.7	0.8
baseline trauma * DrkClass					-0.61	-1.6, 0.42	0.2	0.4					-0.61	-1.6, 0.42	0.2	0.4

Characteristic	Right Lateral															
	Model 1				Model 2				Model 1 (controlling for Drug Use)				Model 2 (controlling for Drug Use)			
	Beta	95% CI[1]	p-value	q-value[2]	Beta	95% CI[1]	p-value	q-value[2]	Beta	95% CI[1]	p-value	q-value[2]	Beta	95% CI[1]	p-value	q-value[2]
age_d	0.84	0.11, 1.6	0.024	0.07	0.71	-0.04, 1.5	0.065	0.2	0.87	0.13, 1.6	0.021	0.081	0.74	-0.03, 1.5	0.06	0.2
age_m	2	0.23, 3.7	0.027	0.07	1.9	0.14, 3.6	0.035	0.11	2	0.23, 3.7	0.027	0.081	1.9	0.14, 3.6	0.035	0.14
baseline trauma	-0.43	-3.6, 2.7	0.8	0.8	-0.07	-3.3, 3.1	>0.9	>0.9	-0.42	-3.6, 2.7	0.8	0.9	-0.08	-3.3, 3.1	>0.9	>0.9
DrkClass	0.54	-0.46, 1.5	0.3	0.5	1.1	-0.30, 2.5	0.12	0.2	0.59	-0.41, 1.6	0.2	0.4	1.2	-0.26, 2.6	0.11	0.3

wholeHippo	0.09	0.08, 0.09	<0.001	**<0.001**	0.09	0.08, 0.09	<0.001	**<0.001**	0.09	0.08, 0.09	<0.001	**<0.001**	0.09	0.08, 0.09	<0.001	**<0.001**
sex																
F	—	—			—	—			—	—			—	—		
M	35	28, 42	<0.001	**<0.001**	35	28, 42	<0.001	**<0.001**	35	28, 42	<0.001	**<0.001**	35	28, 42	<0.001	**<0.001**
ses	1.4	0.05, 2.7	0.042	0.09	1.4	0.07, 2.7	0.039	0.11	1.3	0.03, 2.6	0.045	0.11	1.3	0.05, 2.6	0.042	0.14
family alcohol density	-0.96	-7.9, 6.0	0.8	0.8	-0.97	-7.9, 6.0	0.8	0.8	-0.92	-7.9, 6.1	0.8	0.9	-0.93	-7.9, 6.0	0.8	>0.9
race	6.2	2.8, 9.6	<0.001	**0.002**	6.2	2.8, 9.6	<0.001	**0.002**	6.2	2.8, 9.6	<0.001	**0.002**	6.2	2.8, 9.6	<0.001	**0.002**
LifeTob									0	-0.01, 0.00	0.12	0.3	0	-0.01, 0.00	0.13	0.3
LifeMJ									0	-0.01, 0.01	0.9	0.9	0	-0.01, 0.01	>0.9	>0.9
age_d * age_m	0.11	-0.16, 0.38	0.4	0.6	0.09	-0.18, 0.36	0.5	0.6	0.11	-0.16, 0.38	0.4	0.6	0.09	-0.18, 0.36	0.5	0.7
age_d * baseline trauma	0.11	-0.37, 0.59	0.7	0.8	0.22	-0.29, 0.74	0.4	0.6	0.12	-0.36, 0.59	0.6	0.9	0.23	-0.29, 0.75	0.4	0.6
age_m * baseline trauma	0.15	-1.0, 1.3	0.8	0.8	0.24	-0.93, 1.4	0.7	0.8	0.15	-1.0, 1.3	0.8	0.9	0.24	-0.93, 1.4	0.7	0.8
age_d * age_m * baseline trauma	-0.11	-0.30, 0.07	0.2	0.4	-0.1	-0.28, 0.09	0.3	0.5	-0.11	-0.30, 0.07	0.2	0.4	-0.09	-0.28, 0.09	0.3	0.5
baseline trauma * DrkClass					-0.57	-1.6, 0.42	0.3	0.4					-0.56	-1.6, 0.43	0.3	0.5
Characteristic	Left Basal															
	Model 1				Model 2				Model 1 (controlling for Drug Use)				Model 2 (controlling for Drug Use)			
	Beta	95% CI[1]	p-value	q-value[2]	Beta	95% CI[1]	p-value	q-value[2]	Beta	95% CI[1]	p-value	q-value[2]	Beta	95% CI[1]	p-value	q-value[2]
age_d	0.46	-0.06, 0.99	0.084	0.2	0.34	-0.20, 0.89	0.2	0.4	0.52	-0.01, 1.1	0.055	0.2	0.4	-0.15, 0.96	0.2	0.4
age_m	0.2	-1.1, 1.5	0.8	0.8	0.12	-1.2, 1.4	0.9	0.9	0.22	-1.1, 1.5	0.7	0.7	0.14	-1.2, 1.4	0.8	0.9
baseline trauma	-1.2	-3.5, 1.2	0.3	0.6	-0.82	-3.2, 1.6	0.5	0.6	-1.1	-3.5, 1.2	0.3	0.6	-0.82	-3.2, 1.6	0.5	0.7
DrkClass	0.1	-0.62, 0.82	0.8	0.8	0.66	-0.36, 1.7	0.2	0.4	0.14	-0.58, 0.87	0.7	0.7	0.67	-0.34, 1.7	0.2	0.4
wholeHippo	0.06	0.05, 0.06	<0.001	**<0.001**	0.06	0.05, 0.06	<0.001	**<0.001**	0.06	0.05, 0.06	<0.001	**<0.001**	0.06	0.05, 0.06	<0.001	**<0.001**
sex																
F	—	—			—	—			—	—			—	—		
M	25	20, 30	<0.001	**<0.001**	25	20, 30	<0.001	**<0.001**	25	20, 30	<0.001	**<0.001**	25	20, 30	<0.001	**<0.001**
ses	2.1	1.1, 3.1	<0.001	**<0.001**	2.1	1.1, 3.1	<0.001	**<0.001**	2.1	1.1, 3.0	<0.001	**<0.001**	2.1	1.1, 3.1	<0.001	**<0.001**
family alcohol density	2.3	-2.9, 7.5	0.4	0.6	2.3	-2.9, 7.5	0.4	0.5	2.3	-2.9, 7.6	0.4	0.6	2.3	-2.9, 7.5	0.4	0.6
race	7.3	4.7, 9.9	<0.001	**<0.001**	7.3	4.7, 9.9	<0.001	**<0.001**	7.3	4.7, 9.9	<0.001	**<0.001**	7.3	4.7, 9.9	<0.001	**<0.001**
LifeTob									0	0.00, 0.00	0.7	0.7	0	0.00, 0.00	0.7	0.8
LifeMJ									0	-0.01, 0.00	0.2	0.6	0	-0.01, 0.00	0.3	0.4
age_d * age_m	-0.05	-0.24, 0.15	0.6	0.8	-0.06	-0.26, 0.13	0.5	0.6	-0.04	-0.23, 0.16	0.7	0.7	-0.06	-0.25, 0.14	0.6	0.7
age_d * baseline trauma	0.18	-0.16, 0.53	0.3	0.6	0.29	-0.08, 0.67	0.12	0.3	0.18	-0.16, 0.53	0.3	0.6	0.29	-0.08, 0.66	0.13	0.4
age_m * baseline trauma	0.4	-0.48, 1.3	0.4	0.6	0.48	-0.40, 1.4	0.3	0.4	0.41	-0.47, 1.3	0.4	0.6	0.49	-0.39, 1.4	0.3	0.4
age_d * age_m * baseline trauma	-0.03	-0.17, 0.10	0.6	0.8	-0.01	-0.15, 0.12	0.8	0.9	-0.03	-0.16, 0.11	0.7	0.7	-0.01	-0.15, 0.12	0.9	0.9

Characteristic	Model 1 Beta	Model 1 95% CI[1]	Model 1 p-value	Model 1 q-value[2]	Model 2 Beta	Model 2 95% CI[1]	Model 2 p-value	Model 2 q-value[2]	Model 1 (controlling for Drug Use) Beta	95% CI[1]	p-value	q-value[2]	Model 2 (controlling for Drug Use) Beta	95% CI[1]	p-value	q-value[2]
baseline trauma * DrkClass					-0.55	-1.3, 0.16	0.13	0.3					-0.53	-1.2, 0.18	0.15	0.4

Characteristic	Right Basal															
	Model 1				Model 2				Model 1 (controlling for Drug Use)				Model 2 (controlling for Drug Use)			
	Beta	95% CI[1]	p-value	q-value[2]	Beta	95% CI[1]	p-value	q-value[2]	Beta	95% CI[1]	p-value	q-value[2]	Beta	95% CI[1]	p-value	q-value[2]
age_d	0.61	0.07, 1.1	0.028	0.072	0.44	-0.12, 1.0	0.13	0.2	0.59	0.04, 1.1	0.036	0.11	0.41	-0.16, 0.99	0.2	0.3
age_m	1.1	-0.05, 2.3	0.061	0.13	0.99	-0.17, 2.2	0.1	0.2	1.1	-0.06, 2.3	0.063	0.2	0.98	-0.19, 2.2	0.1	0.2
baseline trauma	0.27	-1.9, 2.4	0.8	0.9	0.74	-1.4, 2.9	0.5	0.6	0.26	-1.9, 2.4	0.8	0.9	0.73	-1.4, 2.9	0.5	0.6
DrkClass	0.54	-0.20, 1.3	0.2	0.3	1.3	0.26, 2.3	0.014	**0.04**	0.53	-0.21, 1.3	0.2	0.3	1.3	0.26, 2.3	0.014	**0.045**
wholeHippo	0.07	0.06, 0.07	<0.001	**<0.001**	0.07	0.06, 0.07	<0.001	**<0.001**	0.07	0.06, 0.07	<0.001	**<0.001**	0.07	0.06, 0.07	<0.001	**<0.001**
sex																
F	—	—			—	—			—	—			—	—		
M	19	15, 24	<0.001	**<0.001**	19	15, 24	<0.001	**<0.001**	19	15, 24	<0.001	**<0.001**	19	15, 24	<0.001	**<0.001**
ses	1.5	0.67, 2.4	<0.001	**0.002**	1.6	0.69, 2.4	<0.001	**0.002**	1.5	0.67, 2.4	<0.001	**0.002**	1.6	0.69, 2.4	<0.001	**0.002**
family alcohol density	1.7	-2.9, 6.4	0.5	0.6	1.7	-2.9, 6.3	0.5	0.6	1.7	-2.9, 6.4	0.5	0.7	1.7	-2.9, 6.3	0.5	0.6
race	4.6	2.4, 6.9	<0.001	**<0.001**	4.6	2.4, 6.9	<0.001	**<0.001**	4.6	2.3, 6.9	<0.001	**<0.001**	4.6	2.3, 6.9	<0.001	**<0.001**
LifeTob									0	0.00, 0.00	0.5	0.7	0	0.00, 0.00	0.5	0.6
LifeMJ									0	-0.01, 0.01	0.6	0.7	0	-0.01, 0.01	0.6	0.6
age_d * age_m	0.05	-0.15, 0.25	0.6	0.7	0.03	-0.17, 0.23	0.8	0.8	0.05	-0.15, 0.25	0.6	0.7	0.03	-0.18, 0.23	0.8	0.8
age_d * baseline trauma	0.14	-0.21, 0.50	0.4	0.6	0.3	-0.09, 0.69	0.13	0.2	0.15	-0.21, 0.50	0.4	0.7	0.3	-0.08, 0.69	0.12	0.2
age_m * baseline trauma	-0.03	-0.81, 0.75	>0.9	>0.9	0.1	-0.69, 0.88	0.8	0.8	-0.03	-0.81, 0.75	>0.9	>0.9	0.09	-0.70, 0.88	0.8	0.8
age_d * age_m * baseline trauma	-0.08	-0.22, 0.06	0.2	0.4	-0.06	-0.20, 0.08	0.4	0.6	-0.08	-0.22, 0.05	0.2	0.4	-0.06	-0.20, 0.08	0.4	0.6
baseline trauma * DrkClass					-0.77	-1.5, -0.04	0.04	0.093					-0.77	-1.5, -0.04	0.038	0.1

Characteristic	Left Accessory Basal															
	Model 1				Model 2				Model 1 (controlling for Drug Use)				Model 2 (controlling for Drug Use)			
	Beta	95% CI[1]	p-value	q-value[2]	Beta	95% CI[1]	p-value	q-value[2]	Beta	95% CI[1]	p-value	q-value[2]	Beta	95% CI[1]	p-value	q-value[2]
age_d	0.34	-0.04, 0.72	0.078	0.2	0.35	-0.04, 0.75	0.082	0.2	0.33	-0.06, 0.71	0.1	0.2	0.34	-0.07, 0.74	0.1	0.3
age_m	0.69	-0.31, 1.7	0.2	0.3	0.7	-0.31, 1.7	0.2	0.3	0.68	-0.32, 1.7	0.2	0.4	0.69	-0.31, 1.7	0.2	0.4
baseline trauma	0.09	-1.7, 1.9	>0.9	>0.9	0.06	-1.8, 1.9	>0.9	>0.9	0.09	-1.7, 1.9	>0.9	>0.9	0.06	-1.8, 1.9	>0.9	>0.9
DrkClass	0.27	-0.26, 0.79	0.3	0.5	0.22	-0.52, 0.96	0.6	0.8	0.27	-0.26, 0.79	0.3	0.6	0.22	-0.51, 0.96	0.6	0.8
wholeHippo	0.04	0.04, 0.04	<0.001	**<0.001**	0.04	0.04, 0.04	<0.001	**<0.001**	0.04	0.04, 0.04	<0.001	**<0.001**	0.04	0.04, 0.04	<0.001	**<0.001**
sex																
F	—	—			—	—			—	—			—	—		
M	15	11, 19	<0.001	**<0.001**	15	11, 19	<0.001	**<0.001**	15	11, 19	<0.001	**<0.001**	15	11, 19	<0.001	**<0.001**
ses	1.2	0.50, 2.0	0.001	**0.004**	1.2	0.50, 2.0	0.001	**0.004**	1.3	0.50, 2.0	0.001	**0.004**	1.2	0.50, 2.0	0.001	**0.005**

family alcohol density	1.8	-2.3, 5.8	0.4	0.6	1.8	-2.3, 5.8	0.4	0.7	1.8	-2.2, 5.8	0.4	0.6	1.8	-2.2, 5.8	0.4	0.8
race	4.1	2.1, 6.1	<0.001	**<0.001**	4.1	2.1, 6.1	<0.001	**<0.001**	4.1	2.1, 6.1	<0.001	**<0.001**	4.1	2.1, 6.1	<0.001	**<0.001**
LifeTob									0	0.00, 0.00	0.5	0.7	0	0.00, 0.00	0.5	0.8
LifeMJ									0	0.00, 0.01	0.6	0.7	0	0.00, 0.01	0.6	0.8
age_d * age_m	-0.13	-0.27, 0.01	0.077	0.2	-0.13	-0.27, 0.02	0.083	0.2	-0.13	-0.27, 0.01	0.072	0.2	-0.13	-0.27, 0.01	0.077	0.2
age_d * baseline trauma	0.07	-0.18, 0.32	0.6	0.7	0.06	-0.21, 0.33	0.6	0.8	0.07	-0.18, 0.32	0.6	0.7	0.07	-0.20, 0.34	0.6	0.8
age_m * baseline trauma	0.16	-0.51, 0.84	0.6	0.7	0.16	-0.52, 0.84	0.7	0.8	0.16	-0.52, 0.84	0.6	0.7	0.15	-0.53, 0.84	0.7	0.8
age_d * age_m * baseline trauma	0.02	-0.07, 0.12	0.6	0.7	0.02	-0.08, 0.12	0.7	0.8	0.02	-0.07, 0.12	0.6	0.7	0.02	-0.08, 0.12	0.7	0.8
baseline trauma * DrkClass					0.05	-0.47, 0.57	0.9	>0.9					0.04	-0.48, 0.57	0.9	>0.9
Characteristic	Right Accessory Basal															
	Model 1				Model 2				Model 1 (controlling for Drug Use)				Model 2 (controlling for Drug Use)			
	Beta	95% CI[1]	p-value	q-value[2]	Beta	95% CI[1]	p-value	q-value[2]	Beta	95% CI[1]	p-value	q-value[2]	Beta	95% CI[1]	p-value	q-value[2]
age_d	0.81	0.39, 1.2	<0.001	**<0.001**	0.76	0.33, 1.2	<0.001	**0.003**	0.79	0.37, 1.2	<0.001	**0.001**	0.74	0.30, 1.2	<0.001	**0.005**
age_m	1.3	0.39, 2.3	0.006	**0.015**	1.3	0.35, 2.2	0.007	**0.021**	1.5	0.48, 2.4	0.003	**0.01**	1.3	0.34, 2.2	0.008	**0.025**
baseline trauma	-0.06	-1.8, 1.7	>0.9	>0.9	0.08	-1.7, 1.8	>0.9	>0.9	-0.16	-1.9, 1.6	0.9	>0.9	0.08	-1.7, 1.8	>0.9	>0.9
DrkClass	-0.02	-0.59, 0.55	>0.9	>0.9	0.2	-0.60, 1.0	0.6	>0.9	-0.04	-0.61, 0.53	0.9	>0.9	0.2	-0.60, 1.0	0.6	>0.9
wholeHippo	0.05	0.04, 0.05	<0.001	**<0.001**	0.05	0.04, 0.05	<0.001	**<0.001**	0.05	0.04, 0.05	<0.001	**<0.001**	0.05	0.04, 0.05	<0.001	**<0.001**
sex																
F	—	—			—	—			—	—			—	—		
M	16	12, 20	<0.001	**<0.001**	16	12, 20	<0.001	**<0.001**	15	11, 19	<0.001	**<0.001**	16	12, 20	<0.001	**<0.001**
ses	0.63	-0.08, 1.3	0.081	0.2	0.64	-0.07, 1.4	0.079	0.2	0.57	-0.16, 1.3	0.12	0.3	0.64	-0.07, 1.4	0.077	0.2
family alcohol density	0.7	-3.1, 4.5	0.7	>0.9	0.7	-3.1, 4.5	0.7	>0.9	0.72	-3.1, 4.6	0.7	0.9	0.7	-3.1, 4.5	0.7	>0.9
race	3.1	1.2, 5.0	0.002	**0.005**	3.1	1.2, 5.0	0.002	**0.006**	3.1	1.2, 5.0	0.001	**0.005**	3.1	1.2, 5.0	0.002	**0.007**
LifeTob									0	0.00, 0.00	0.7	0.9	0	0.00, 0.00	0.7	>0.9
LifeMJ									0	0.00, 0.01	0.6	0.9	0	0.00, 0.01	0.6	>0.9
age_d * age_m	-0.01	-0.16, 0.15	>0.9	>0.9	-0.01	-0.17, 0.14	0.8	>0.9	-0.01	-0.16, 0.15	>0.9	>0.9	-0.02	-0.17, 0.14	0.8	>0.9
age_d * baseline trauma	-0.07	-0.34, 0.20	0.6	>0.9	-0.02	-0.32, 0.27	0.9	>0.9	-0.06	-0.34, 0.21	0.6	0.9	-0.02	-0.31, 0.27	0.9	>0.9
age_m * baseline trauma	-0.1	-0.74, 0.54	0.8	>0.9	-0.06	-0.71, 0.58	0.8	>0.9	-0.12	-0.77, 0.52	0.7	0.9	-0.07	-0.71, 0.58	0.8	>0.9
age_d * age_m * baseline trauma	-0.08	-0.19, 0.03	0.14	0.3	-0.07	-0.18, 0.03	0.2	0.4	-0.08	-0.19, 0.02	0.13	0.3	-0.07	-0.18, 0.03	0.2	0.4
baseline trauma * DrkClass					-0.23	-0.79, 0.34	0.4	0.8					-0.23	-0.80, 0.33	0.4	0.8
Characteristic	Left AAA															
	Model 1				Model 2				Model 1 (controlling for Drug Use)				Model 2 (controlling for Drug Use)			
	Beta	95% CI[1]	p-value	q-value[2]	Beta	95% CI[1]	p-value	q-value[2]	Beta	95% CI[1]	p-value	q-value[2]	Beta	95% CI[1]	p-value	q-value[2]
age_d	0.03	-0.09, 0.16	0.6	0.7	0.02	-0.11, 0.15	0.8	0.8	0.05	-0.08, 0.17	0.5	0.8	0.03	-0.10, 0.17	0.6	0.8

age_m	-0.07	-0.32, 0.18	0.6	0.7	-0.08	-0.33, 0.17	0.5	0.8	-0.07	-0.32, 0.18	0.6	0.8	-0.08	-0.33, 0.17	0.5	0.8
baseline trauma	0.05	-0.40, 0.50	0.8	0.8	0.09	-0.38, 0.55	0.7	0.8	0.06	-0.39, 0.51	0.8	0.8	0.09	-0.37, 0.55	0.7	0.8
DrkClass	0.05	-0.13, 0.22	0.6	0.7	0.1	-0.14, 0.34	0.4	0.8	0.05	-0.12, 0.23	0.5	0.8	0.11	-0.14, 0.35	0.4	0.8
wholeHippo	0.01	0.01, 0.01	<0.001	**<0.001**	0.01	0.01, 0.01	<0.001	**<0.001**	0.01	0.01, 0.01	<0.001	**<0.001**	0.01	0.01, 0.01	<0.001	**<0.001**
sex																
F	—	—			—	—			—	—			—	—		
M	2.3	1.3, 3.3	<0.001	**<0.001**	2.3	1.3, 3.3	<0.001	**<0.001**	2.3	1.3, 3.3	<0.001	**<0.001**	2.3	1.3, 3.3	<0.001	**<0.001**
ses	0.2	0.02, 0.39	0.034	0.11	0.2	0.02, 0.39	0.033	0.12	0.2	0.01, 0.38	0.038	0.14	0.2	0.01, 0.39	0.037	0.15
family alcohol density	0.24	-0.75, 1.2	0.6	0.7	0.24	-0.75, 1.2	0.6	0.8	0.24	-0.75, 1.2	0.6	0.8	0.24	-0.75, 1.2	0.6	0.8
race	0.6	0.11, 1.1	0.016	0.07	0.6	0.11, 1.1	0.016	0.075	0.61	0.12, 1.1	0.015	0.077	0.61	0.12, 1.1	0.015	0.082
LifeTob									0	0.00, 0.00	0.8	0.8	0	0.00, 0.00	0.8	0.8
LifeMJ									0	0.00, 0.00	0.2	0.6	0	0.00, 0.00	0.3	0.7
age_d * age_m	0.02	-0.03, 0.06	0.5	0.7	0.01	-0.03, 0.06	0.6	0.8	0.02	-0.03, 0.06	0.5	0.8	0.02	-0.03, 0.06	0.5	0.8
age_d * baseline trauma	0.03	-0.06, 0.11	0.5	0.7	0.04	-0.05, 0.13	0.4	0.8	0.03	-0.06, 0.11	0.5	0.8	0.04	-0.05, 0.13	0.4	0.8
age_m * baseline trauma	0.11	-0.05, 0.28	0.2	0.5	0.12	-0.05, 0.29	0.2	0.4	0.12	-0.05, 0.28	0.2	0.5	0.12	-0.05, 0.29	0.2	0.5
age_d * age_m * baseline trauma	-0.01	-0.04, 0.02	0.6	0.7	-0.01	-0.04, 0.03	0.7	0.8	-0.01	-0.04, 0.03	0.7	0.8	-0.01	-0.04, 0.03	0.8	0.8
baseline trauma * DrkClass					-0.06	-0.23, 0.11	0.5	0.8					-0.05	-0.22, 0.12	0.5	0.8

Characteristic	Right AAA															
	Model 1				Model 2				Model 1 (controlling for Drug Use)				Model 2 (controlling for Drug Use)			
	Beta	95% CI[1]	p-value	q-value[2]	Beta	95% CI[1]	p-value	q-value[2]	Beta	95% CI[1]	p-value	q-value[2]	Beta	95% CI[1]	p-value	q-value[2]
age_d	0.15	0.01, 0.29	0.03	0.1	0.13	-0.01, 0.27	0.074	0.2	0.16	0.02, 0.30	0.024	0.11	0.14	-0.01, 0.28	0.062	0.2
age_m	0.26	0.00, 0.52	0.047	0.12	0.24	-0.01, 0.50	0.064	0.2	0.26	0.01, 0.52	0.045	0.14	0.25	-0.01, 0.50	0.061	0.2
baseline trauma	-0.28	-0.75, 0.19	0.2	0.4	-0.22	-0.70, 0.26	0.4	0.5	-0.28	-0.74, 0.19	0.2	0.5	-0.22	-0.70, 0.26	0.4	0.6
DrkClass	0.03	-0.15, 0.21	0.7	0.8	0.13	-0.13, 0.39	0.3	0.5	0.04	-0.15, 0.22	0.7	0.8	0.13	-0.13, 0.39	0.3	0.6
wholeHippo	0.01	0.01, 0.01	<0.001	**<0.001**	0.01	0.01, 0.01	<0.001	**<0.001**	0.01	0.01, 0.01	<0.001	**<0.001**	0.01	0.01, 0.01	<0.001	**<0.001**
sex																
F	—	—			—	—			—	—			—	—		
M	3.7	2.7, 4.8	<0.001	**<0.001**	3.7	2.7, 4.8	<0.001	**<0.001**	3.7	2.7, 4.8	<0.001	**<0.001**	3.7	2.7, 4.8	<0.001	**<0.001**
ses	0.22	0.02, 0.41	0.027	0.1	0.22	0.03, 0.41	0.026	0.12	0.21	0.02, 0.41	0.03	0.11	0.22	0.02, 0.41	0.028	0.15
family alcohol density	0.84	-0.19, 1.9	0.11	0.2	0.84	-0.19, 1.9	0.11	0.2	0.84	-0.18, 1.9	0.11	0.3	0.84	-0.18, 1.9	0.11	0.3
race	0.4	-0.10, 0.91	0.12	0.2	0.41	-0.10, 0.91	0.12	0.2	0.41	-0.10, 0.92	0.12	0.3	0.41	-0.10, 0.92	0.12	0.3
LifeTob									0	0.00, 0.00	0.8	0.8	0	0.00, 0.00	0.8	0.8
LifeMJ									0	0.00, 0.00	0.5	0.8	0	0.00, 0.00	0.6	0.7
age_d * age_m	0.01	-0.04, 0.06	0.8	0.8	0	-0.05, 0.05	>0.9	>0.9	0.01	-0.04, 0.06	0.8	0.8	0	-0.05, 0.05	0.9	0.9

age_d * baseline trauma	0.02	-0.07, 0.11	0.7	0.8	0.04	-0.06, 0.14	0.4	0.5	0.02	-0.07, 0.11	0.7	0.8	0.04	-0.06, 0.14	0.4	0.6
age_m * baseline trauma	-0.05	-0.22, 0.12	0.6	0.7	-0.04	-0.21, 0.14	0.7	0.7	-0.05	-0.22, 0.12	0.6	0.8	-0.03	-0.21, 0.14	0.7	0.8
age_d * age_m * baseline trauma	-0.02	-0.05, 0.02	0.3	0.4	-0.02	-0.05, 0.02	0.4	0.5	-0.02	-0.05, 0.02	0.3	0.5	-0.01	-0.05, 0.02	0.4	0.6
baseline trauma * DrkClass					-0.1	-0.28, 0.08	0.3	0.5					-0.1	-0.28, 0.09	0.3	0.6
Characteristic	Left Central															
	Model 1				Model 2				Model 1 (controlling for Drug Use)				Model 2 (controlling for Drug Use)			
	Beta	95% CI[1]	p-value	q-value[2]	Beta	95% CI[1]	p-value	q-value[2]	Beta	95% CI[1]	p-value	q-value[2]	Beta	95% CI[1]	p-value	q-value[2]
age_d	0.12	-0.02, 0.25	0.088	0.2	0.13	-0.01, 0.27	0.066	0.2	0.11	-0.03, 0.24	0.11	0.3	0.12	-0.02, 0.26	0.085	0.2
age_m	0.1	-0.10, 0.31	0.3	0.5	0.12	-0.09, 0.32	0.3	0.5	0.1	-0.10, 0.31	0.3	0.6	0.11	-0.09, 0.32	0.3	0.6
baseline trauma	0.18	-0.19, 0.55	0.3	0.5	0.14	-0.24, 0.53	0.5	0.6	0.18	-0.19, 0.55	0.3	0.6	0.14	-0.24, 0.53	0.5	0.6
DrkClass	0.04	-0.14, 0.21	0.7	0.7	-0.03	-0.28, 0.22	0.8	0.9	0.03	-0.14, 0.21	0.7	0.8	-0.03	-0.28, 0.22	0.8	0.9
wholeHippo	0.01	0.01, 0.01	<0.001	**<0.001**	0.01	0.01, 0.01	<0.001	**<0.001**	0.01	0.01, 0.01	<0.001	**<0.001**	0.01	0.01, 0.01	<0.001	**<0.001**
sex																
F	—	—			—	—			—	—			—	—		
M	2.6	1.8, 3.5	<0.001	**<0.001**	2.6	1.8, 3.5	<0.001	**<0.001**	2.6	1.8, 3.5	<0.001	**<0.001**	2.6	1.8, 3.5	<0.001	**<0.001**
ses	0.24	0.09, 0.39	0.002	**0.01**	0.24	0.08, 0.39	0.002	**0.012**	0.24	0.09, 0.39	0.002	**0.011**	0.24	0.09, 0.39	0.002	**0.013**
family alcohol density	0.46	-0.35, 1.3	0.3	0.5	0.46	-0.35, 1.3	0.3	0.5	0.46	-0.35, 1.3	0.3	0.6	0.46	-0.35, 1.3	0.3	0.6
race	0.47	0.08, 0.87	0.019	0.063	0.47	0.08, 0.87	0.02	0.069	0.47	0.07, 0.87	0.02	0.069	0.47	0.07, 0.87	0.02	0.082
LifeTob									0	0.00, 0.00	0.4	0.6	0	0.00, 0.00	0.4	0.6
LifeMJ									0	0.00, 0.00	0.4	0.6	0	0.00, 0.00	0.5	0.6
age_d * age_m	-0.06	-0.11, -0.01	0.026	0.068	-0.05	-0.10, 0.00	0.032	0.091	-0.06	-0.11, -0.01	0.023	0.069	-0.06	-0.11, -0.01	0.029	0.092
age_d * baseline trauma	0.01	-0.08, 0.09	0.9	0.9	-0.01	-0.10, 0.09	0.9	0.9	0.01	-0.08, 0.09	0.9	0.9	-0.01	-0.10, 0.09	0.9	0.9
age_m * baseline trauma	0.05	-0.09, 0.18	0.5	0.6	0.04	-0.10, 0.18	0.6	0.7	0.05	-0.09, 0.18	0.5	0.6	0.04	-0.10, 0.18	0.6	0.7
age_d * age_m * baseline trauma	0.01	-0.02, 0.05	0.4	0.5	0.01	-0.02, 0.05	0.5	0.6	0.01	-0.02, 0.05	0.4	0.6	0.01	-0.02, 0.05	0.5	0.6
baseline trauma * DrkClass					0.07	-0.11, 0.24	0.5	0.6					0.06	-0.11, 0.24	0.5	0.6
Characteristic	Right Central															
	Model 1				Model 2				Model 1 (controlling for Drug Use)				Model 2 (controlling for Drug Use)			
	Beta	95% CI[1]	p-value	q-value[2]	Beta	95% CI[1]	p-value	q-value[2]	Beta	95% CI[1]	p-value	q-value[2]	Beta	95% CI[1]	p-value	q-value[2]
age_d	0.16	0.02, 0.31	0.03	0.13	0.15	0.00, 0.30	0.05	0.2	0.16	0.01, 0.30	0.036	0.2	0.15	-0.01, 0.30	0.061	0.3
age_m	0.17	-0.05, 0.39	0.12	0.3	0.16	-0.06, 0.38	0.15	0.4	0.17	-0.05, 0.39	0.13	0.4	0.16	-0.06, 0.38	0.2	0.5
baseline trauma	0.14	-0.25, 0.53	0.5	0.7	0.17	-0.24, 0.58	0.4	0.7	0.14	-0.25, 0.53	0.5	0.8	0.17	-0.24, 0.58	0.4	0.7
DrkClass	0.02	-0.18, 0.21	0.9	0.9	0.06	-0.21, 0.33	0.7	0.8	0.01	-0.18, 0.21	0.9	0.9	0.06	-0.21, 0.33	0.7	0.7
wholeHippo	0.01	0.01, 0.01	<0.001	**<0.001**	0.01	0.01, 0.01	<0.001	**<0.001**	0.01	0.01, 0.01	<0.001	**<0.001**	0.01	0.01, 0.01	<0.001	**<0.001**
sex																

F	—	—			—	—			—	—			—	—		
M	2.4	1.6, 3.3	<0.001	**<0.001**	2.4	1.6, 3.3	<0.001	**<0.001**	2.4	1.5, 3.3	<0.001	**<0.001**	2.4	1.5, 3.3	<0.001	**<0.001**
ses	0.07	-0.09, 0.23	0.4	0.6	0.07	-0.09, 0.24	0.4	0.7	0.07	-0.09, 0.24	0.4	0.7	0.07	-0.09, 0.24	0.4	0.7
family alcohol density	0.15	-0.71, 1.0	0.7	0.8	0.15	-0.71, 1.0	0.7	0.8	0.16	-0.70, 1.0	0.7	0.8	0.15	-0.71, 1.0	0.7	0.8
race	0.22	-0.20, 0.64	0.3	0.6	0.22	-0.20, 0.64	0.3	0.6	0.22	-0.21, 0.64	0.3	0.7	0.22	-0.21, 0.64	0.3	0.7
LifeTob									0	0.00, 0.00	0.6	0.8	0	0.00, 0.00	0.6	0.7
LifeMJ									0	0.00, 0.00	0.7	0.8	0	0.00, 0.00	0.6	0.7
age_d * age_m	-0.03	-0.08, 0.02	0.3	0.6	-0.03	-0.08, 0.02	0.3	0.6	-0.03	-0.08, 0.02	0.3	0.7	-0.03	-0.09, 0.02	0.3	0.7
age_d * baseline trauma	0.03	-0.07, 0.12	0.6	0.8	0.03	-0.07, 0.14	0.5	0.7	0.03	-0.07, 0.12	0.6	0.8	0.04	-0.07, 0.14	0.5	0.7
age_m * baseline trauma	-0.03	-0.17, 0.12	0.7	0.8	-0.02	-0.17, 0.13	0.8	0.8	-0.03	-0.17, 0.12	0.7	0.8	-0.02	-0.17, 0.13	0.8	0.8
age_d * age_m * baseline trauma	-0.03	-0.07, 0.01	0.13	0.3	-0.03	-0.06, 0.01	0.2	0.4	-0.03	-0.07, 0.01	0.13	0.4	-0.03	-0.07, 0.01	0.15	0.5
baseline trauma * DrkClass					-0.05	-0.23, 0.14	0.6	0.8					-0.05	-0.24, 0.14	0.6	0.7

Characteristic	Left Cortical															
	Model 1				Model 2				Model 1 (controlling for Drug Use)				Model 2 (controlling for Drug Use)			
	Beta	95% CI[1]	p-value	q-value[2]	Beta	95% CI[1]	p-value	q-value[2]	Beta	95% CI[1]	p-value	q-value[2]	Beta	95% CI[1]	p-value	q-value[2]
age_d	-0.07	-0.14, 0.00	0.048	0.13	-0.06	-0.13, 0.01	0.12	0.3	-0.08	-0.15, -0.01	0.023	0.069	-0.07	-0.14, 0.00	0.061	0.2
age_m	-0.02	-0.15, 0.12	0.8	0.9	-0.01	-0.14, 0.13	>0.9	>0.9	-0.02	-0.15, 0.12	0.8	0.8	-0.01	-0.15, 0.12	0.9	>0.9
baseline trauma	0.12	-0.12, 0.37	0.3	0.5	0.09	-0.16, 0.34	0.5	0.8	0.12	-0.13, 0.36	0.3	0.5	0.09	-0.16, 0.34	0.5	0.7
DrkClass	0.06	-0.03, 0.15	0.2	0.4	0.01	-0.12, 0.14	>0.9	>0.9	0.05	-0.04, 0.15	0.3	0.5	0	-0.13, 0.13	>0.9	>0.9
wholeHippo	0	0.00, 0.00	<0.001	**<0.001**	0	0.00, 0.00	<0.001	**<0.001**	0	0.00, 0.00	<0.001	**<0.001**	0	0.00, 0.00	<0.001	**<0.001**
sex																
F	—	—			—	—			—	—			—	—		
M	1.3	0.79, 1.9	<0.001	**<0.001**	1.3	0.79, 1.9	<0.001	**<0.001**	1.3	0.76, 1.9	<0.001	**<0.001**	1.3	0.76, 1.9	<0.001	**<0.001**
ses	0.08	-0.02, 0.18	0.12	0.2	0.08	-0.02, 0.18	0.12	0.3	0.08	-0.02, 0.19	0.1	0.2	0.08	-0.02, 0.18	0.11	0.2
family alcohol density	0.22	-0.31, 0.76	0.4	0.6	0.23	-0.31, 0.76	0.4	0.7	0.22	-0.31, 0.76	0.4	0.6	0.22	-0.31, 0.76	0.4	0.7
race	0.06	-0.20, 0.33	0.6	0.8	0.06	-0.20, 0.33	0.6	0.9	0.06	-0.21, 0.32	0.7	0.8	0.06	-0.21, 0.32	0.7	0.9
LifeTob									0	0.00, 0.00	0.3	0.5	0	0.00, 0.00	0.3	0.5
LifeMJ									0	0.00, 0.00	0.034	0.084	0	0.00, 0.00	0.039	0.2
age_d * age_m	-0.03	-0.05, 0.00	0.027	0.086	-0.03	-0.05, 0.00	0.037	0.2	-0.03	-0.06, -0.01	0.018	0.068	-0.03	-0.05, 0.00	0.026	0.14
age_d * baseline trauma	0.06	0.01, 0.10	0.014	0.061	0.05	0.00, 0.09	0.066	0.2	0.06	0.01, 0.10	0.014	0.068	0.05	0.00, 0.09	0.06	0.2
age_m * baseline trauma	0	-0.09, 0.09	>0.9	>0.9	-0.01	-0.10, 0.08	0.9	>0.9	0	-0.09, 0.09	>0.9	>0.9	-0.01	-0.10, 0.08	0.8	>0.9
age_d * age_m * baseline trauma	0	-0.01, 0.02	0.7	0.8	0	-0.02, 0.02	0.8	>0.9	0	-0.01, 0.02	0.7	0.8	0	-0.02, 0.02	0.9	>0.9
baseline trauma * DrkClass					0.05	-0.04, 0.15	0.2	0.5					0.05	-0.04, 0.14	0.3	0.5

Characteristic	Right Cortical

	Model 1				Model 2				Model 1 (controlling for Drug Use)				Model 2 (controlling for Drug Use)			
	Beta	95% CI[1]	p-value	q-value[2]	Beta	95% CI[1]	p-value	q-value[2]	Beta	95% CI[1]	p-value	q-value[2]	Beta	95% CI[1]	p-value	q-value[2]
age_d	0.1	0.03, 0.17	0.007	**0.032**	0.11	0.03, 0.18	0.005	**0.025**	0.1	0.03, 0.17	0.008	**0.038**	0.11	0.03, 0.18	0.006	**0.03**
age_m	0.13	0.01, 0.25	0.035	0.11	0.14	0.01, 0.26	0.029	0.1	0.13	0.01, 0.25	0.034	0.13	0.14	0.01, 0.26	0.028	0.11
baseline trauma	-0.03	-0.25, 0.19	0.8	0.8	-0.06	-0.28, 0.17	0.6	0.7	-0.03	-0.25, 0.19	0.8	0.9	-0.06	-0.28, 0.17	0.6	0.7
DrkClass	-0.04	-0.14, 0.06	0.4	0.7	-0.08	-0.21, 0.06	0.3	0.5	-0.04	-0.14, 0.06	0.4	0.8	-0.08	-0.21, 0.06	0.3	0.6
wholeHippo	0	0.00, 0.00	<0.001	**<0.001**	0	0.00, 0.00	<0.001	**<0.001**	0	0.00, 0.00	<0.001	**<0.001**	0	0.00, 0.00	<0.001	**<0.001**
sex																
F	—	—			—	—			—	—			—	—		
M	2	1.5, 2.5	<0.001	**<0.001**	2	1.5, 2.5	<0.001	**<0.001**	2	1.5, 2.5	<0.001	**<0.001**	2	1.5, 2.5	<0.001	**<0.001**
ses	0.03	-0.06, 0.12	0.5	0.7	0.03	-0.06, 0.12	0.5	0.7	0.03	-0.06, 0.12	0.5	0.8	0.03	-0.06, 0.12	0.5	0.7
family alcohol density	0.18	-0.31, 0.66	0.5	0.7	0.18	-0.31, 0.66	0.5	0.7	0.18	-0.31, 0.66	0.5	0.8	0.18	-0.31, 0.66	0.5	0.7
race	0.13	-0.11, 0.37	0.3	0.5	0.13	-0.11, 0.37	0.3	0.5	0.13	-0.11, 0.37	0.3	0.6	0.13	-0.11, 0.37	0.3	0.6
LifeTob									0	0.00, 0.00	0.6	0.8	0	0.00, 0.00	0.6	0.7
LifeMJ									0	0.00, 0.00	0.9	>0.9	0	0.00, 0.00	0.8	0.8
age_d * age_m	-0.02	-0.04, 0.01	0.2	0.5	-0.01	-0.04, 0.01	0.3	0.5	-0.02	-0.04, 0.01	0.2	0.6	-0.01	-0.04, 0.01	0.3	0.6
age_d * baseline trauma	0	-0.05, 0.05	>0.9	>0.9	-0.01	-0.06, 0.04	0.8	0.8	0	-0.05, 0.05	>0.9	>0.9	-0.01	-0.06, 0.04	0.8	0.8
age_m * baseline trauma	-0.07	-0.15, 0.01	0.1	0.3	-0.07	-0.16, 0.01	0.076	0.2	-0.07	-0.15, 0.01	0.1	0.3	-0.07	-0.16, 0.01	0.077	0.2
age_d * age_m * baseline trauma	0	-0.02, 0.01	0.7	0.8	-0.01	-0.02, 0.01	0.6	0.7	0	-0.02, 0.01	0.7	0.8	-0.01	-0.02, 0.01	0.6	0.7
baseline trauma * DrkClass					0.04	-0.06, 0.13	0.5	0.7					0.04	-0.06, 0.13	0.5	0.7
Characteristic	Left Corticoamygdaloid Transition Area															
	Model 1				Model 2				Model 1 (controlling for Drug Use)				Model 2 (controlling for Drug Use)			
	Beta	95% CI[1]	p-value	q-value[2]	Beta	95% CI[1]	p-value	q-value[2]	Beta	95% CI[1]	p-value	q-value[2]	Beta	95% CI[1]	p-value	q-value[2]
age_d	-0.05	-0.25, 0.14	0.6	0.9	-0.05	-0.26, 0.15	0.6	>0.9	-0.04	-0.24, 0.16	0.7	0.9	-0.04	-0.25, 0.17	0.7	>0.9
age_m	0.18	-0.42, 0.77	0.6	0.9	0.17	-0.42, 0.77	0.6	>0.9	0.18	-0.41, 0.77	0.6	0.8	0.18	-0.41, 0.77	0.6	0.9
baseline trauma	0.03	-1.1, 1.1	>0.9	>0.9	0.04	-1.1, 1.1	>0.9	>0.9	0.04	-1.0, 1.1	>0.9	>0.9	0.04	-1.1, 1.1	>0.9	>0.9
DrkClass	0.05	-0.23, 0.32	0.7	>0.9	0.06	-0.32, 0.44	0.8	>0.9	0.06	-0.22, 0.33	0.7	0.9	0.07	-0.32, 0.45	0.7	>0.9
wholeHippo	0.01	0.01, 0.02	<0.001	**<0.001**	0.01	0.01, 0.02	<0.001	**<0.001**	0.01	0.01, 0.02	<0.001	**<0.001**	0.01	0.01, 0.02	<0.001	**<0.001**
sex																
F	—	—			—	—			—	—			—	—		
M	14	12, 16	<0.001	**<0.001**	14	12, 16	<0.001	**<0.001**	14	12, 16	<0.001	**<0.001**	14	12, 16	<0.001	**<0.001**
ses	0.5	0.06, 0.95	0.026	0.085	0.5	0.06, 0.95	0.026	0.092	0.5	0.05, 0.94	0.028	0.11	0.5	0.05, 0.94	0.028	0.11
family alcohol density	1.3	-1.1, 3.7	0.3	0.6	1.3	-1.1, 3.7	0.3	0.7	1.3	-1.1, 3.7	0.3	0.7	1.3	-1.1, 3.7	0.3	0.8
race	3	1.8, 4.1	<0.001	**<0.001**	3	1.8, 4.1	<0.001	**<0.001**	3	1.8, 4.1	<0.001	**<0.001**	3	1.8, 4.1	<0.001	**<0.001**

LifeTob									0	0.00, 0.00	0.5	0.8	0	0.00, 0.00	0.5	0.9
LifeMJ									0	0.00, 0.00	0.5	0.8	0	0.00, 0.00	0.5	0.9
age_d * age_m	-0.05	-0.13, 0.02	0.15	0.4	-0.05	-0.13, 0.02	0.15	0.4	-0.05	-0.12, 0.02	0.2	0.5	-0.05	-0.13, 0.02	0.2	0.5
age_d * baseline trauma	0.02	-0.11, 0.15	0.8	>0.9	0.02	-0.12, 0.16	0.8	>0.9	0.02	-0.11, 0.15	0.8	0.9	0.02	-0.12, 0.16	0.8	>0.9
age_m * baseline trauma	0.16	-0.24, 0.56	0.4	0.8	0.16	-0.24, 0.57	0.4	0.9	0.16	-0.24, 0.56	0.4	0.8	0.16	-0.24, 0.57	0.4	0.9
age_d * age_m * baseline trauma	0	-0.05, 0.05	0.9	>0.9	0.01	-0.05, 0.06	0.8	>0.9	0.01	-0.04, 0.06	0.8	0.9	0.01	-0.04, 0.06	0.8	>0.9
baseline trauma * DrkClass					-0.01	-0.29, 0.26	>0.9	>0.9					-0.01	-0.28, 0.26	>0.9	>0.9
Characteristic	Right Corticoamygdaloid Transition Area															
	Model 1				Model 2				Model 1 (controlling for Drug Use)				Model 2 (controlling for Drug Use)			
	Beta	95% CI[1]	p-value	q-value[2]	Beta	95% CI[1]	p-value	q-value[2]	Beta	95% CI[1]	p-value	q-value[2]	Beta	95% CI[1]	p-value	q-value[2]
age_d	0.18	-0.03, 0.40	0.1	0.2	0.16	-0.06, 0.39	0.2	0.4	0.22	0.01, 0.44	0.045	0.11	0.21	-0.02, 0.44	0.077	0.2
age_m	0.56	0.01, 1.1	0.045	0.15	0.55	0.00, 1.1	0.052	0.2	0.58	0.02, 1.1	0.041	0.11	0.56	0.01, 1.1	0.047	0.2
baseline trauma	-0.04	-1.0, 0.96	>0.9	>0.9	0.02	-1.0, 1.0	>0.9	>0.9	-0.03	-1.0, 0.98	>0.9	>0.9	0.02	-1.00, 1.0	>0.9	>0.9
DrkClass	-0.14	-0.44, 0.16	0.4	0.6	-0.05	-0.47, 0.37	0.8	>0.9	-0.12	-0.42, 0.18	0.4	0.7	-0.04	-0.46, 0.38	0.9	>0.9
wholeHippo	0.02	0.02, 0.02	<0.001	**<0.001**	0.02	0.02, 0.02	<0.001	**<0.001**	0.02	0.02, 0.02	<0.001	**<0.001**	0.02	0.02, 0.02	<0.001	**<0.001**
sex																
F	—	—			—	—			—	—			—	—		
M	14	12, 16	<0.001	**<0.001**	14	12, 16	<0.001	**<0.001**	14	12, 16	<0.001	**<0.001**	14	12, 16	<0.001	**<0.001**
ses	0.12	-0.29, 0.53	0.6	0.7	0.12	-0.29, 0.53	0.6	0.8	0.11	-0.31, 0.52	0.6	0.8	0.11	-0.30, 0.52	0.6	0.9
family alcohol density	0.76	-1.4, 3.0	0.5	0.7	0.76	-1.4, 3.0	0.5	0.8	0.77	-1.4, 3.0	0.5	0.7	0.77	-1.4, 3.0	0.5	0.9
race	2.2	1.1, 3.3	<0.001	**<0.001**	2.2	1.1, 3.3	<0.001	**<0.001**	2.2	1.1, 3.3	<0.001	**<0.001**	2.2	1.1, 3.3	<0.001	**<0.001**
LifeTob									0	0.00, 0.00	>0.9	>0.9	0	0.00, 0.00	>0.9	>0.9
LifeMJ									0	-0.01, 0.00	0.04	0.11	0	-0.01, 0.00	0.043	0.2
age_d * age_m	-0.04	-0.12, 0.04	0.3	0.6	-0.05	-0.13, 0.03	0.3	0.6	-0.04	-0.12, 0.04	0.3	0.7	-0.04	-0.12, 0.04	0.3	0.7
age_d * baseline trauma	-0.03	-0.17, 0.12	0.7	0.8	-0.01	-0.16, 0.15	>0.9	>0.9	-0.03	-0.17, 0.12	0.7	0.8	-0.01	-0.16, 0.14	>0.9	>0.9
age_m * baseline trauma	-0.19	-0.56, 0.18	0.3	0.6	-0.18	-0.55, 0.20	0.4	0.7	-0.18	-0.55, 0.19	0.3	0.7	-0.17	-0.54, 0.21	0.4	0.8
age_d * age_m * baseline trauma	-0.02	-0.07, 0.04	0.5	0.7	-0.01	-0.07, 0.04	0.6	0.8	-0.01	-0.07, 0.04	0.6	0.8	-0.01	-0.07, 0.04	0.7	0.9
baseline trauma * DrkClass					-0.1	-0.39, 0.20	0.5	0.8					-0.08	-0.38, 0.22	0.6	0.9
Characteristic	Left Medial															
	Model 1				Model 2				Model 1 (controlling for Drug Use)				Model 2 (controlling for Drug Use)			
	Beta	95% CI[1]	p-value	q-value[2]	Beta	95% CI[1]	p-value	q-value[2]	Beta	95% CI[1]	p-value	q-value[2]	Beta	95% CI[1]	p-value	q-value[2]
age_d	-0.06	-0.17, 0.05	0.3	0.5	-0.03	-0.15, 0.09	0.6	0.7	-0.07	-0.18, 0.05	0.3	0.5	-0.03	-0.15, 0.09	0.6	0.6
age_m	0.11	-0.07, 0.28	0.2	0.5	0.13	-0.05, 0.31	0.2	0.4	0.1	-0.07, 0.28	0.2	0.5	0.13	-0.05, 0.31	0.2	0.4
baseline trauma	-0.05	-0.37, 0.27	0.8	0.8	-0.13	-0.47, 0.20	0.4	0.7	-0.05	-0.37, 0.27	0.8	0.8	-0.14	-0.47, 0.20	0.4	0.6

DrkClass	0.07	-0.08, 0.22	0.4	0.5	-0.08	-0.29, 0.14	0.5	0.7	0.07	-0.08, 0.22	0.4	0.6	-0.07	-0.29, 0.14	0.5	0.6
wholeHippo	0	0.00, 0.00	<0.001	**<0.001**	0	0.00, 0.00	<0.001	**<0.001**	0	0.00, 0.00	<0.001	**<0.001**	0	0.00, 0.00	<0.001	**<0.001**
sex																
F	—	—			—	—			—	—			—	—		
M	1.4	0.73, 2.2	<0.001	**<0.001**	1.4	0.73, 2.2	<0.001	**<0.001**	1.4	0.71, 2.2	<0.001	**<0.001**	1.4	0.71, 2.2	<0.001	**<0.001**
ses	0.11	-0.02, 0.24	0.1	0.3	0.11	-0.02, 0.24	0.11	0.3	0.11	-0.02, 0.24	0.1	0.4	0.11	-0.02, 0.24	0.11	0.3
family alcohol density	0.35	-0.35, 1.0	0.3	0.5	0.35	-0.35, 1.1	0.3	0.7	0.35	-0.35, 1.1	0.3	0.5	0.35	-0.35, 1.1	0.3	0.6
race	0.06	-0.28, 0.41	0.7	0.8	0.06	-0.28, 0.41	0.7	0.7	0.06	-0.29, 0.41	0.7	0.8	0.06	-0.29, 0.40	0.7	0.7
LifeTob									0	0.00, 0.00	0.2	0.5	0	0.00, 0.00	0.2	0.5
LifeMJ									0	0.00, 0.00	0.4	0.6	0	0.00, 0.00	0.5	0.6
age_d * age_m	-0.06	-0.10, -0.01	0.009	**0.039**	-0.05	-0.09, -0.01	0.016	0.074	-0.06	-0.10, -0.02	0.008	**0.038**	-0.05	-0.10, -0.01	0.014	0.073
age_d * baseline trauma	0.05	-0.02, 0.13	0.2	0.5	0.02	-0.06, 0.10	0.6	0.7	0.05	-0.02, 0.13	0.2	0.5	0.02	-0.06, 0.10	0.6	0.6
age_m * baseline trauma	-0.03	-0.14, 0.09	0.6	0.8	-0.05	-0.17, 0.07	0.4	0.7	-0.03	-0.15, 0.09	0.6	0.8	-0.05	-0.17, 0.07	0.4	0.6
age_d * age_m * baseline trauma	0	-0.03, 0.02	0.8	0.8	-0.01	-0.04, 0.02	0.6	0.7	0	-0.03, 0.02	0.8	0.8	-0.01	-0.04, 0.02	0.6	0.6
baseline trauma * DrkClass					0.14	-0.01, 0.29	0.06	0.2					0.14	-0.01, 0.29	0.064	0.3
Characteristic	Right Medial															
	Model 1				Model 2				Model 1 (controlling for Drug Use)				Model 2 (controlling for Drug Use)			
	Beta	95% CI[1]	p-value	q-value[2]	Beta	95% CI[1]	p-value	q-value[2]	Beta	95% CI[1]	p-value	q-value[2]	Beta	95% CI[1]	p-value	q-value[2]
age_d	0.09	-0.03, 0.21	0.15	0.4	0.08	-0.04, 0.21	0.2	0.4	0.08	-0.04, 0.21	0.2	0.4	0.08	-0.05, 0.20	0.2	0.5
age_m	0.22	0.05, 0.40	0.014	0.06	0.22	0.04, 0.39	0.016	0.077	0.22	0.04, 0.40	0.014	0.072	0.22	0.04, 0.39	0.017	0.092
baseline trauma	-0.03	-0.35, 0.29	0.8	0.8	-0.02	-0.34, 0.31	>0.9	>0.9	-0.03	-0.35, 0.28	0.8	>0.9	-0.02	-0.35, 0.31	>0.9	>0.9
DrkClass	-0.02	-0.17, 0.14	0.8	0.8	0.01	-0.21, 0.23	>0.9	>0.9	-0.02	-0.18, 0.14	0.8	>0.9	0.01	-0.22, 0.23	>0.9	>0.9
wholeHippo	0	0.00, 0.00	<0.001	**<0.001**	0	0.00, 0.00	<0.001	**<0.001**	0	0.00, 0.00	<0.001	**<0.001**	0	0.00, 0.00	<0.001	**<0.001**
sex																
F	—	—			—	—			—	—			—	—		
M	1.5	0.82, 2.3	<0.001	**<0.001**	1.5	0.82, 2.3	<0.001	**<0.001**	1.5	0.81, 2.2	<0.001	**<0.001**	1.5	0.81, 2.2	<0.001	**<0.001**
ses	0.04	-0.09, 0.17	0.6	0.7	0.04	-0.09, 0.17	0.6	0.8	0.04	-0.09, 0.17	0.6	0.8	0.04	-0.09, 0.17	0.5	0.9
family alcohol density	-0.42	-1.1, 0.28	0.2	0.4	-0.42	-1.1, 0.28	0.2	0.4	-0.42	-1.1, 0.28	0.2	0.4	-0.42	-1.1, 0.28	0.2	0.5
race	0.33	-0.01, 0.68	0.056	0.2	0.33	-0.01, 0.68	0.056	0.2	0.33	-0.01, 0.68	0.057	0.2	0.33	-0.01, 0.68	0.057	0.2
LifeTob									0	0.00, 0.00	>0.9	>0.9	0	0.00, 0.00	>0.9	>0.9
LifeMJ									0	0.00, 0.00	0.7	>0.9	0	0.00, 0.00	0.7	>0.9
age_d * age_m	-0.02	-0.06, 0.02	0.4	0.5	-0.02	-0.07, 0.02	0.4	0.6	-0.02	-0.06, 0.02	0.4	0.6	-0.02	-0.07, 0.02	0.4	0.6
age_d * baseline trauma	0.05	-0.03, 0.13	0.2	0.4	0.06	-0.03, 0.14	0.2	0.4	0.05	-0.03, 0.13	0.2	0.4	0.06	-0.03, 0.14	0.2	0.5
age_m * baseline trauma	-0.08	-0.19, 0.04	0.2	0.4	-0.07	-0.19, 0.05	0.2	0.4	-0.08	-0.19, 0.04	0.2	0.4	-0.07	-0.19, 0.05	0.2	0.5

age_d * age_m * baseline trauma	0	-0.03, 0.03	0.8	0.8	0	-0.03, 0.03	0.9	>0.9	0	-0.03, 0.03	0.8	>0.9	0	-0.03, 0.03	0.9	>0.9
baseline trauma * DrkClass					-0.02	-0.18, 0.13	0.8	>0.9					-0.03	-0.18, 0.13	0.7	>0.9
Characteristic	Left Paralaminar															
	Model 1				Model 2				Model 1 (controlling for Drug Use)				Model 2 (controlling for Drug Use)			
	Beta	95% CI[1]	p-value	q-value[2]	Beta	95% CI[1]	p-value	q-value[2]	Beta	95% CI[1]	p-value	q-value[2]	Beta	95% CI[1]	p-value	q-value[2]
age_d	-0.05	-0.11, 0.01	0.1	0.2	-0.07	-0.13, -0.01	0.033	0.091	-0.04	-0.10, 0.02	0.2	0.4	-0.06	-0.12, 0.01	0.076	0.2
age_m	-0.02	-0.19, 0.16	0.8	>0.9	-0.03	-0.21, 0.15	0.7	0.8	-0.01	-0.19, 0.16	0.9	>0.9	-0.03	-0.20, 0.15	0.8	0.8
baseline trauma	-0.13	-0.45, 0.19	0.4	0.7	-0.09	-0.41, 0.24	0.6	0.8	-0.13	-0.45, 0.19	0.4	0.6	-0.08	-0.41, 0.24	0.6	0.7
DrkClass	0.02	-0.06, 0.10	0.6	0.8	0.1	-0.02, 0.21	0.092	0.2	0.03	-0.05, 0.11	0.5	0.6	0.1	-0.01, 0.22	0.082	0.2
wholeHippo	0	0.00, 0.00	<0.001	**<0.001**	0	0.00, 0.00	<0.001	**<0.001**	0	0.00, 0.00	<0.001	**<0.001**	0	0.00, 0.00	<0.001	**<0.001**
sex																
F	—	—			—	—			—	—			—	—		
M	4.6	3.9, 5.3	<0.001	**<0.001**	4.6	3.9, 5.3	<0.001	**<0.001**	4.6	3.9, 5.3	<0.001	**<0.001**	4.6	3.9, 5.3	<0.001	**<0.001**
ses	0.22	0.09, 0.35	<0.001	**0.003**	0.22	0.09, 0.35	<0.001	**0.003**	0.22	0.09, 0.35	0.001	**0.004**	0.22	0.09, 0.35	0.001	**0.004**
family alcohol density	0.26	-0.44, 0.96	0.5	0.7	0.26	-0.44, 0.96	0.5	0.6	0.27	-0.43, 0.97	0.5	0.6	0.27	-0.43, 0.97	0.5	0.6
race	0.75	0.41, 1.1	<0.001	**<0.001**	0.75	0.41, 1.1	<0.001	**<0.001**	0.75	0.41, 1.1	<0.001	**<0.001**	0.75	0.41, 1.1	<0.001	**<0.001**
LifeTob									0	0.00, 0.00	0.4	0.6	0	0.00, 0.00	0.4	0.6
LifeMJ									0	0.00, 0.00	0.084	0.3	0	0.00, 0.00	0.1	0.2
age_d * age_m	0	-0.02, 0.02	>0.9	>0.9	0	-0.03, 0.02	0.8	0.8	0	-0.02, 0.02	>0.9	>0.9	0	-0.02, 0.02	0.9	0.9
age_d * baseline trauma	0.02	-0.02, 0.06	0.4	0.7	0.03	-0.01, 0.08	0.12	0.2	0.02	-0.02, 0.06	0.4	0.6	0.03	-0.01, 0.08	0.12	0.2
age_m * baseline trauma	0.08	-0.04, 0.20	0.2	0.4	0.09	-0.03, 0.21	0.13	0.2	0.08	-0.04, 0.20	0.2	0.4	0.09	-0.03, 0.21	0.12	0.2
age_d * age_m * baseline trauma	0	-0.01, 0.02	>0.9	>0.9	0	-0.01, 0.02	0.7	0.8	0	-0.01, 0.02	0.9	>0.9	0	-0.01, 0.02	0.7	0.8
baseline trauma * DrkClass					-0.08	-0.16, 0.00	0.058	0.13					-0.07	-0.16, 0.01	0.071	0.2
Characteristic	Right Paralaminar															
	Model 1				Model 2				Model 1 (controlling for Drug Use)				Model 2 (controlling for Drug Use)			
	Beta	95% CI[1]	p-value	q-value[2]	Beta	95% CI[1]	p-value	q-value[2]	Beta	95% CI[1]	p-value	q-value[2]	Beta	95% CI[1]	p-value	q-value[2]
age_d	-0.04	-0.09, 0.02	0.2	0.5	-0.05	-0.11, 0.01	0.087	0.2	-0.03	-0.09, 0.03	0.3	0.6	-0.05	-0.11, 0.01	0.13	0.3
age_m	0.03	-0.15, 0.20	0.8	0.9	0.02	-0.16, 0.19	0.9	0.9	0.03	-0.15, 0.20	0.7	0.8	0.02	-0.16, 0.19	0.9	0.9
baseline trauma	0.17	-0.15, 0.49	0.3	0.5	0.22	-0.10, 0.54	0.2	0.3	0.17	-0.14, 0.49	0.3	0.6	0.22	-0.10, 0.54	0.2	0.3
DrkClass	0.01	-0.07, 0.09	0.8	>0.9	0.09	-0.02, 0.20	0.13	0.2	0.01	-0.07, 0.09	0.7	0.8	0.09	-0.02, 0.21	0.11	0.3
wholeHippo	0	0.00, 0.00	<0.001	**<0.001**	0	0.00, 0.00	<0.001	**<0.001**	0	0.00, 0.00	<0.001	**<0.001**	0	0.00, 0.00	<0.001	**<0.001**
sex																
F	—	—			—	—			—	—			—	—		
M	3.5	2.9, 4.2	<0.001	**<0.001**	3.5	2.9, 4.2	<0.001	**<0.001**	3.6	2.9, 4.2	<0.001	**<0.001**	3.6	2.9, 4.2	<0.001	**<0.001**

ses	0.25	0.12, 0.38	<0.001	**<0.001**	0.25	0.12, 0.38	<0.001	**<0.001**	0.25	0.12, 0.38	<0.001	**<0.001**	0.25	0.12, 0.38	<0.001	**<0.001**
family alcohol density	0.2	-0.49, 0.90	0.6	0.8	0.2	-0.49, 0.90	0.6	0.7	0.21	-0.49, 0.90	0.6	0.8	0.21	-0.49, 0.90	0.6	0.6
race	0.45	0.11, 0.79	0.011	**0.034**	0.45	0.11, 0.79	0.01	**0.036**	0.45	0.11, 0.79	0.01	**0.039**	0.45	0.11, 0.79	0.01	**0.041**
LifeTob									0	0.00, 0.00	0.3	0.6	0	0.00, 0.00	0.3	0.4
LifeMJ									0	0.00, 0.00	0.5	0.8	0	0.00, 0.00	0.5	0.6
age_d * age_m	-0.01	-0.03, 0.02	0.6	0.8	-0.01	-0.03, 0.01	0.4	0.6	-0.01	-0.03, 0.02	0.6	0.8	-0.01	-0.03, 0.01	0.5	0.6
age_d * baseline trauma	0.03	0.00, 0.07	0.074	0.2	0.05	0.01, 0.09	0.016	**0.045**	0.04	0.00, 0.07	0.07	0.2	0.05	0.01, 0.09	0.016	**0.05**
age_m * baseline trauma	0.03	-0.09, 0.14	0.7	0.9	0.04	-0.08, 0.16	0.5	0.7	0.03	-0.09, 0.14	0.6	0.8	0.04	-0.08, 0.16	0.5	0.6
age_d * age_m * baseline trauma	0	-0.01, 0.01	>0.9	>0.9	0	-0.01, 0.02	0.7	0.8	0	-0.01, 0.02	>0.9	>0.9	0	-0.01, 0.02	0.7	0.8
baseline trauma * DrkClass					-0.08	-0.16, 0.00	0.049	0.11					-0.08	-0.16, 0.00	0.054	0.15
Characteristic	Left Whole Amygdala															
	Model 1				Model 2				Model 1 (controlling for Drug Use)				Model 2 (controlling for Drug Use)			
	Beta	95% CI[1]	p-value	q-value[2]	Beta	95% CI[1]	p-value	q-value[2]	Beta	95% CI[1]	p-value	q-value[2]	Beta	95% CI[1]	p-value	q-value[2]
age_d	3.4	1.7, 5.1	<0.001	**<0.001**	3.3	1.5, 5.0	<0.001	**0.001**	3.5	1.8, 5.2	<0.001	**<0.001**	3.4	1.6, 5.2	<0.001	**0.001**
age_m	-3	-8.3, 2.3	0.3	0.4	-3.1	-8.5, 2.2	0.2	0.4	-3	-8.3, 2.3	0.3	0.4	-3.1	-8.4, 2.2	0.3	0.5
baseline trauma	-5.3	-15, 4.4	0.3	0.4	-4.9	-15, 4.8	0.3	0.5	-5.2	-15, 4.4	0.3	0.4	-4.9	-15, 4.9	0.3	0.5
DrkClass	0.24	-2.1, 2.6	0.8	>0.9	0.86	-2.4, 4.1	0.6	0.7	0.31	-2.0, 2.7	0.8	>0.9	0.89	-2.4, 4.2	0.6	0.7
wholeHippo	0	0.00, 0.00	<0.001	**<0.001**	0	0.00, 0.00	<0.001	**<0.001**	0	0.00, 0.00	<0.001	**<0.001**	0	0.00, 0.00	<0.001	**<0.001**
sex																
F	—	—			—	—			—	—			—	—		
M	93	71, 116	<0.001	**<0.001**	93	71, 116	<0.001	**<0.001**	94	71, 116	<0.001	**<0.001**	94	71, 116	<0.001	**<0.001**
ses	2.4	-1.5, 6.4	0.2	0.4	2.4	-1.5, 6.4	0.2	0.4	2.4	-1.6, 6.3	0.2	0.4	2.4	-1.6, 6.4	0.2	0.5
family alcohol density	20	-1.5, 40	0.069	0.2	20	-1.5, 40	0.069	0.2	20	-1.4, 41	0.068	0.2	20	-1.4, 41	0.068	0.2
race	13	2.6, 24	0.015	**0.049**	13	2.6, 24	0.015	0.052	13	2.6, 24	0.015	0.055	13	2.6, 24	0.015	0.058
LifeTob									0	-0.01, 0.01	>0.9	>0.9	0	-0.01, 0.01	>0.9	>0.9
LifeMJ									-0.01	-0.03, 0.02	0.5	0.7	-0.01	-0.03, 0.02	0.5	0.7
age_d * age_m	-0.54	-1.2, 0.08	0.087	0.2	-0.56	-1.2, 0.06	0.078	0.2	-0.53	-1.2, 0.10	0.1	0.2	-0.55	-1.2, 0.08	0.088	0.2
age_d * baseline trauma	0.06	-1.0, 1.2	>0.9	>0.9	0.19	-1.0, 1.4	0.8	0.8	0.07	-1.0, 1.2	>0.9	>0.9	0.18	-1.0, 1.4	0.8	0.8
age_m * baseline trauma	2.1	-1.5, 5.6	0.2	0.4	2.2	-1.4, 5.8	0.2	0.4	2.1	-1.4, 5.7	0.2	0.4	2.2	-1.4, 5.8	0.2	0.5
age_d * age_m * baseline trauma	0.1	-0.33, 0.52	0.7	0.8	0.12	-0.32, 0.55	0.6	0.7	0.1	-0.32, 0.53	0.6	0.8	0.12	-0.31, 0.56	0.6	0.7
baseline trauma * DrkClass					-0.62	-2.9, 1.7	0.6	0.7					-0.58	-2.9, 1.7	0.6	0.7
Characteristic	Right Whole Amygdala															
	Model 1				Model 2				Model 1 (controlling for Drug Use)				Model 2 (controlling for Drug Use)			
	Beta	95% CI[1]	p-value	q-value[2]	Beta	95% CI[1]	p-value	q-value[2]	Beta	95% CI[1]	p-value	q-value[2]	Beta	95% CI[1]	p-value	q-value[2]

age_d	4.9	3.2, 6.7	<0.001	**<0.001**	4.7	2.8, 6.5	<0.001	**<0.001**	5.1	3.2, 6.9	<0.001	**<0.001**	4.8	2.9, 6.7	<0.001	**<0.001**
age_m	0.03	-4.9, 5.0	>0.9	>0.9	-0.16	-5.1, 4.8	>0.9	>0.9	0.07	-4.9, 5.0	>0.9	>0.9	-0.12	-5.1, 4.8	>0.9	>0.9
baseline trauma	-3.6	-12, 5.4	0.4	0.7	-2.8	-12, 6.3	0.5	0.7	-3.5	-12, 5.4	0.4	0.8	-2.8	-12, 6.3	0.5	0.7
DrkClass	0.32	-2.2, 2.8	0.8	>0.9	1.6	-1.9, 5.1	0.4	0.7	0.4	-2.1, 2.9	0.8	>0.9	1.6	-1.9, 5.1	0.4	0.7
wholeHippo	0	0.00, 0.00	<0.001	**<0.001**	0	0.00, 0.00	<0.001	**<0.001**	0	0.00, 0.00	<0.001	**<0.001**	0	0.00, 0.00	<0.001	**<0.001**
sex																
F	—	—			—	—			—	—			—	—		
M	91	70, 112	<0.001	**<0.001**	91	70, 113	<0.001	**<0.001**	92	71, 113	<0.001	**<0.001**	92	71, 113	<0.001	**<0.001**
ses	-0.09	-3.8, 3.6	>0.9	>0.9	-0.05	-3.8, 3.7	>0.9	>0.9	-0.13	-3.9, 3.6	>0.9	>0.9	-0.09	-3.8, 3.6	>0.9	>0.9
family alcohol density	15	-5.1, 34	0.14	0.4	15	-5.1, 34	0.15	0.4	15	-5.0, 35	0.14	0.4	15	-5.0, 35	0.14	0.5
race	8.9	-1.1, 19	0.083	0.3	8.9	-1.1, 19	0.082	0.3	9	-1.1, 19	0.081	0.3	9	-1.1, 19	0.081	0.3
LifeTob									0	-0.01, 0.01	0.8	>0.9	0	-0.01, 0.01	0.8	>0.9
LifeMJ									-0.01	-0.03, 0.02	0.6	>0.9	-0.01	-0.03, 0.02	0.6	0.7
age_d * age_m	-0.16	-0.83, 0.51	0.6	0.9	-0.2	-0.87, 0.47	0.6	0.7	-0.15	-0.81, 0.52	0.7	>0.9	-0.18	-0.86, 0.49	0.6	0.7
age_d * baseline trauma	-0.71	-1.9, 0.47	0.2	0.5	-0.45	-1.7, 0.83	0.5	0.7	-0.7	-1.9, 0.48	0.2	0.6	-0.45	-1.7, 0.82	0.5	0.7
age_m * baseline trauma	0.69	-2.6, 4.0	0.7	0.9	0.89	-2.5, 4.2	0.6	0.7	0.7	-2.6, 4.0	0.7	>0.9	0.9	-2.5, 4.3	0.6	0.7
age_d * age_m * baseline trauma	-0.25	-0.71, 0.21	0.3	0.5	-0.21	-0.68, 0.25	0.4	0.7	-0.25	-0.71, 0.21	0.3	0.6	-0.21	-0.67, 0.26	0.4	0.7
baseline trauma * DrkClass					-1.3	-3.8, 1.2	0.3	0.7					-1.2	-3.7, 1.2	0.3	0.7

Supplementary Table 4 . Effect sizes for model predictors on hippocampal subfield and amygdala nuclei volumes. Generalized additive mixed models (GAMM) output from gamm4 package in R. [1] CI = Confidence Interval; [2] False discovery rate correction for multiple testing
